# Diversity and signature of small RNA in different bodily fluids using next generation sequencing

**DOI:** 10.1186/s12864-018-4785-8

**Published:** 2018-05-29

**Authors:** Mohamed El-Mogy, Bernard Lam, Taha A. Haj-Ahmad, Shannon McGowan, Darrick Yu, Lucas Nosal, Nezar Rghei, Pam Roberts, Yousef Haj-Ahmad

**Affiliations:** 1 Norgen Biotek Corp, Thorold, ON L2V 4Y6 Canada; 2 Molecular Biology Department, National Research Centre, Dokki, Giza, Egypt; 3 Department of Biological Sciences, Brock University, St. Catharines, ON L2S 3A1 Canada

**Keywords:** miRNA, tRNA, piRNA, Next generation sequencing, Blood, Plasma, Serum, Saliva, Urine

## Abstract

**Background:**

Small RNAs are critical components in regulating various cellular pathways. These molecules may be tissue-associated or circulating in bodily fluids and have been shown to associate with different tumors. Next generation sequencing (NGS) on small RNAs is a powerful tool for profiling and discovery of microRNAs (miRNAs).

**Results:**

In this study, we isolated total RNA from various bodily fluids: blood, leukocytes, serum, plasma, saliva, cell-free saliva, urine and cell-free urine. Next, we used Illumina’s NGS platform and intensive bioinformatics analysis to investigate the distribution and signature of small RNAs in the various fluids. Successful NGS was accomplished despite the variations in RNA concentrations among the different fluids. Among the fluids studied, blood and plasma were found to be the most promising fluids for small RNA profiling as well as novel miRNA prediction. Saliva and urine yielded lower numbers of identifiable molecules and therefore were less reliable in small RNA profiling and less useful in predicting novel molecules. In addition, all fluids shared many molecules, including 139 miRNAs, the most abundant tRNAs, and the most abundant piwi-interacting RNAs (piRNAs). Fluids of similar origin (blood, urine or saliva) displayed closer clustering, while each fluid still retains its own characteristic signature based on its unique molecules and its levels of the common molecules. Donor urine samples showed sex-dependent differential clustering, which may prove useful for future studies.

**Conclusions:**

This study shows the successful clustering and unique signatures of bodily fluids based on their miRNA, tRNA and piRNA content. With this information, cohorts may be differentiated based on multiple molecules from each small RNA class by a multidimensional assessment of the overall molecular signature.

**Electronic supplementary material:**

The online version of this article (10.1186/s12864-018-4785-8) contains supplementary material, which is available to authorized users.

## Background

Small RNAs are a class of mainly non-coding RNAs (ncRNAs) characterized by their small nucleotide length of less than 200 nt [1]. Within this class there are key RNA types with a size range of 14–35 nt that are highly important for diagnostic biomarker discovery and the development of therapeutic agents [2–4]. These include microRNAs (miRNAs), transfer RNA-derived RNAs (tDRs) and Piwi-interacting RNAs (piRNAs). MicroRNAs are non-coding molecules of about 19–23 nt that bind to and downregulate messenger RNAs (mRNAs) [5]. They down regulate gene expression, playing a major role in essential biological pathways, such as differentiation, proliferation, metastasis and apoptosis. [6–11]. MicroRNAs represent an entire layer of gene expression regulation, regulating more than 50% of protein coding mRNAs in mammalian cells [12]. To date, 2588 human mature miRNAs have been identified and are currently included in miRBase 21 [13]. Aside from being found in tissues and cells, miRNAs are found in bodily fluids in extracellular vesicles or in complexes with argonaute or lipoproteins [14–17]. They have been reported in bodily fluids such as blood, plasma, serum, urine, tears, saliva, breast milk, amniotic fluid, seminal fluid and colostrum [18–23]. MicroRNAs have been linked to many diseases and are highly promising molecular biomarkers [24–27].

Mature tRNAs and nascent pre-tRNA transcripts are processed enzymatically to produce well defined tDRs in a regulated process, suggesting that they are not random degradation products [28, 29]. Sizes of tDRs range from 30 to 35 nt for tRNA halves and 14 to 26 nt for the shorter fragments [4]. Various studies suggest that tDRs are involved in different functions including stress responses in human diseases, where they act as inhibitors of global translation and transcription regulation [30–36]. Like miRNAs, they can conduct specific gene silencing and have potential as cancer biomarkers [7, 37–47]. Finally, piRNAs have a nucleotide size range between 26 to 31. They modulate different gene expression pathways by interacting with Piwi proteins [48, 49]. Currently there are 23,439 piRNA molecules in the piRNABank [50]. They are abundant in gonads and mediate transposon repression to conserve genome integrity [51–53].

The use of next generation sequencing (NGS) technology in small RNA detection has advanced research in the field at unprecedented speed. NGS shines light on the key role of ncRNAs in transcriptome regulation in healthy and disease conditions and accelerates the profiling and discovery of molecules [54]. The technology enables the analysis of multiple samples in parallel and provides precise quantification of each molecule, making it superior to previous genomic technologies. The demonstrated capacities of NGS have led to advances in biological and medical genomics and transcriptomics [55–57].

Solid tumor miRNAs are well represented in bodily fluids, indicating their importance as cancer biomarkers [58–60]. Almost all bodily fluids from healthy individuals contain miRNAs. Therefore, bodily fluids represent an excellent candidate for non-invasive detection of miRNAs and have been used in applications such as cancer biomarker discovery [8]. Transfer RNA-derived small RNAs are thought to have a dual function, as they act as suppressors and oncogenes [42]. In addition, altered piRNAs levels were found to be associated with lung, breast, gastric and colon cancers [61–64]. However, no comprehensive study has been reported on tDRs or piRNAs in different bodily fluids.

The field of small RNA is expanding with the profiling and discovery of molecules in various disease conditions and treatments. Therefore, it is important to explore the small RNA content in normal individuals to better understand the small RNA profile in each fluid as well as their relative distribution among the different fluids. To gain insights into the distribution and signature of small RNA in bodily fluids, we carried out a comparative study on RNA from different fluids collected from the same donors and used NGS to explore and describe their small RNA content.

## Methods

### Sample collection, preparation and RNA purification

Blood, saliva and urine were collected from 4 healthy donors, 2 females and 2 males between the ages of 20 to 30. The various bodily fluids were collected from each individual within a 2-h period. Collection and sample de-identification was performed under an IRB approved protocol (16198–16:02:416–11-2017). Three 10 mL blood samples were collected from each donor. Two of these samples were collected in Vacutainer® plastic EDTA tubes (BD, USA) and used for RNA isolation from whole blood, leukocytes and plasma. RNA was isolated directly from 0.2 mL of blood using the Total RNA Purification Plus Kit (Norgen Biotek Corp., Canada). Leukocytes were prepared from 0.5 mL of blood by using the Leukocyte RNA Purification Kit (Norgen Biotek Corp., Canada). One entire tube was used to prepare plasma and was centrifuged at 200 RCF for 10 min at room temperature. Plasma was collected and stored at − 70 °C until isolation. The last blood sample was collected in a Vacutainer® glass serum tube with silicon coated interior (BD, USA) and used for RNA isolation from serum. The tube was left to stand at room temperature for 45 min, and then it was centrifuged at 1300 RCF for 15 min. Serum was collected and stored at − 70 °C until isolation. Both plasma and serum RNA were isolated from 0.2 mL using the Plasma/Serum RNA Isolation Mini Kit (Norgen Biotek Corp., Canada). All kits were used according to the manufacturer’s instructions.

Two milliliters of saliva was collected from each donor into Falcon 50 mL centrifuge tubes (BD, USA) and 0.3 mL was used directly for saliva RNA isolation using the Total RNA Purification Kit (Norgen Biotek Corp., Canada), according to the manufacturer’s instructions. Another 0.3 mL of saliva was transferred into a 1.5 mL tube (Eppendorf, Germany) and spun down at 200 RCF for 10 min to remove cells, and then the supernatant was used for cell-free saliva RNA purification by the same kit. A similar approach was used for urine sample preparation, where 100 mL of urine was collected from each donor into disposable cups (Sarstedt, Germany). RNA was isolated from 30 mL of urine using the Urine Cell-Free Circulating RNA Purification Maxi Kit (Norgen Biotek Corp., Canada). The kit’s procedure was modified by skipping the initial centrifugation steps to purify RNA from total urine. Another urine sample was processed by the same kit without any modifications to the manufacturer’s procedures to isolate RNA from 30 mL of cell-free urine. Purified RNA from all samples were tested for positive amplification by miR-21 stem-loop RT-PCR [65]. RNA concentration was then estimated by the Agilent 2100 Bioanalyzer System (Agilent Technologies, USA) using the RNA 6000 Nano Total RNA chip.

### Small RNA library construction and high-throughput sequencing

The small RNA libraries were prepared from the RNA isolated from each sample using the Small RNA Library Prep Kit for Illumina (Norgen Biotek Corp., Canada) according to the manufacturer’s instructions. Briefly, 6 μL of purified RNA was mixed with the 3′ adapter and incubated at 70 °C for 2 min before being used in a ligation step by adding T4 RNA ligase 2 (truncated), buffer and RNase inhibitor. The reaction was incubated at 28 °C for 1 h then heat inactivated at 70 °C for 10 min. The excess 3′ adapters were removed by the addition of the reverse primer and incubating the reaction at 75 °C for 5 min, 37 °C for 15 min and 25 °C for 5 min. The 5′ adapter was denatured at 70 °C for 2 min and then added together with 10 mM ATP and T4 RNA ligase 1 to the reaction and incubated at 28 °C for 1 h followed by heat inactivation at 70 °C for 10 min. The two adapters were diluted 1:1 before being added to the reactions and all the incubation steps were performed in a thermocycler with cooling on ice between the different steps. Reverse transcription was performed on the ligation reaction product by adding a mixture containing 10 mM dNTPs, first strand buffer and TruScript reverse transcriptase, and incubating the reaction at 50 °C for 1 h before heat inactivation at 70 °C for 15 min. Finally, the reverse transcription reaction product was amplified and indexed in a 15 cycle PCR reaction by adding the NGS PCR master mix, PCR reverse primer and the unique index primer for each sample.

The PCR reaction product was cleaned and separated on a 6% Novex® TBE PAGE gel (Life Technologies, USA). The gel was stained with SYBR® Gold Nucleic Acid Gel Stain (Life Technologies, USA) and a library size range from 125 bp to 170 bp was excised from the gel and placed in a Gel Breaker Tube (IST Engineering, USA), then centrifuged at 14000 RCF for 2 min. The prepared libraries were then eluted overnight in nuclease-free water (Ambion, USA) and cleaned. The library was quantified by the High Sensitivity DNA Analysis Kit on the Agilent 2100 Bioanalyzer System (Agilent Technologies, USA). Libraries were diluted to 4 nM, pooled, and sequenced on the Illumina HiSeq 4000 at The McGill University and Génome Québec Innovation Centre (Montreal, Canada), using the HiSeq 3000/4000 SBS Kit (50 cycles).

### Read mapping and small RNA annotation

The sequence raw data from the Illumina HiSeq 4000 were converted to fastq format. Files were then used in the Genboree Workbench’s exceRpt small RNA-seq pipeline (version 4.6.2) for read mapping to the hg38 human genome version [66]. This allowed for a single mismatched base down to 18 nucleotides. After adapter trimming, read quality was assessed by FASTQC to filter out reads with a quality score lower than 30 on the PHRED scale. Reads were first mapped to the UniVec and human ribosomal RNA (rRNA) sequences to exclude them before mapping to databases of miRBase version 21, gtRNAdb and piRNABank to assign reads to miRNAs, tRNAs and piRNAs, respectively. Identified tRNAs are tRNA-derived RNA fragments due to the fact that the library insert size is below 50 nt. Remaining sequences were then annotated to gencode version 24 (hg38) which includes protein coding transcripts (protein_coding), mitochondrial rRNA (Mt_rRNA), mitochondrial tRNA (Mt_tRNA), small nuclear RNA (snRNA), small nucleolar RNA (snoRNA), long intergenic noncoding RNA (lincRNA) and miscellaneous RNA (misc_RNA).

### Data analysis

Raw read counts obtained from the Genboree Workbench’s exceRpt small RNA-seq pipeline were further analyzed using R (version 3.4.0) and R studio (version 1.0.143). The following R packages were used in the analysis: RnaSeqGeneEdgeRQL (version 1.0.0) for counts per million (CPM) filtration and normalization by using trimmed mean of M-values (TMM) [67], ggfortify (version 0.4.1) and ComplexHeatmap (version 1.14.0) for principal component analysis (PCA) plot and heatmaps based on the filtered and normalized data, respectively. VennDiagram (version 1.6.17) was used to illustrate Venn diagrams. miRDeep2 (version 2.0.0.8) was used to predict novel miRNA candidates and tDRmapper was used to identify tDRs.

## Results

Small RNA profiles in the various bodily fluids used in this study provide an atlas of miRNAs, tRNAs and piRNAs relative distribution. They also provide in depth molecular analysis and a guide for NGS-based small RNA expression studies that employ one or more of these bodily fluids as a source of biological data. It is important to look at the normal characteristics of small RNA molecules in each fluid in terms of abundance and representation. The origin and nature of these fluids can pose a significant effect on their use in certain studies that might require specific handling during preparation and sequencing to ensure the validity of results.

### RNA concentration variations in the different bodily fluids

Concentration of RNA from each bodily fluid tested was measured using an Agilent 2100 Bioanalyzer. The average range of RNA content in 1 L of bodily fluids was as low as 0.01 mg in urine to as high as 11.2 mg in saliva. Bodily fluids can be categorized based on their RNA content; significantly higher amounts of RNA can be recovered from saliva, blood and cell-free saliva (4.2 to 11.2 mg/L). Leukocytes, serum and plasma had moderate yields of 0.8 to 1.8 mg/L, while urine and cell-free urine had significantly lower RNA content of 0.01 mg/L. Blood, leukocytes, saliva and cell-free saliva had lower standard deviations in their RNA content (< 50% of average), whereas plasma, serum, urine and cell-free urine had higher deviations between their samples (70–85% of average). The average concentration of the isolated RNA from all bodily fluids ranged from 67.2 ng/μL to 3.4 ng/μL. They can be classified into high (> 20 ng/μL) from blood, leukocytes, saliva and cell-free saliva and low (< 10 ng/μL) from plasma, serum, urine and cell-free urine. The RNA integrity number (RIN) was more than 7 for RNA from leukocytes and lower for RNA from blood, saliva and cell-free saliva (about 2–3). RNA from serum, urine and cell-free urine had a low RIN of 1 or less, while RNA from plasma had no measurable RIN from any sample (Table 1).

**Table 1:** Variations in RNA concentration, RIN value, and yield among the different bodily fluids

Bodily fluid	Concentration (ng/uL)		RIN Value		RNA amount in 1 L of fluid (mg)	
	Ave	STDEV	Ave	STDEV	Ave	STDEV
Blood	21.200	4.764	2.740	2.243	5.300	1.191
Leukocytes	18.200	5.848	7.600	0.474	1.820	0.585
Plasma	3.400	2.881	N/A	N/A	0.850	0.720
Serum	6.600	5.320	1.000	N/A	1.650	1.330
Saliva	67.200	33.056	2.360	0.602	11.200	5.509
Cell-Free Saliva	25.333	10.970	2.133	1.002	4.222	1.828
Urine	5.750	4.113	1.000	N/A	0.010	0.007
Cell-Free Urine	6.600	5.683	1.000	N/A	0.011	0.009

RNA concentration and RIN value were determined by the Agilent 2100 Bioanalyzer System

### Input read alignment

Reads obtained from sequencing were used for alignment and mapping to the human genome after adapter clipping and quality filtering. The range of average input reads from the various bodily fluids was between 9.5 million reads (serum) to 15.7 million reads (blood). The average input reads from all the bodily fluid samples tested was 12.57 ± 3.54 million reads. The descending order of samples based on their number of input reads was: blood, cell-free urine, urine, leukocyte, plasma, cell-free saliva, saliva and serum. The percentage of successfully clipped reads was more than 60% from all sample types, with a minimum percentage of reads failing quality filters. Reads were mapped to human rRNA to exclude rRNA sequences before mapping to human genome. The average percentage of reads aligned to human rRNA was less than 12% in all bodily fluids except saliva, cell-free saliva and leukocytes, which had average percentages of 16.6 ± 7.5, 13.0 ± 6.2 and 36.0 ± 1.5, respectively. This is indirectly proportional to reads used for alignment to the human genome. More than 50% of reads used for alignment were mapped to the human genome in blood, plasma, serum, urine and cell-free urine. The percentage was lower in the leukocytes as well as total and cell-free samples of saliva.

In saliva and cell-free saliva, the average percentage of unmapped reads was about 50% of reads used for alignment (48.9 ± 19.7% and 50.3 ± 10.1% of input reads, respectively). Conversely, urine and cell-free urine had an average percentage of unmapped reads relative to reads used in alignment of 21.2 ± 17.8% and 25.5 ± 23.9%, respectively. The percentage of input reads alignment from each bodily fluid can be found in Table 2.

**Table 2:** Percentage of input reads alignment from each bodily fluid

Bodily Fluid	Blood		Leukocytes		Plasma		Serum		Saliva		Cell-Free Saliva		Urine		Cell-Free Urine	
	Ave	SDEV	Ave	SDEV	Ave	SDEV	Ave	SDEV	Ave	SDEV	Ave	SDEV	Ave	SDEV	Ave	SDEV
^a^Input (million reads)	15.7	2.3	12.5	1.0	12.4	6.7	9.5	1.5	10.4	1.1	10.9	1.4	14.1	2.6	15.1	4.2
Successfully clipped	94.4	0.7	63.4	4.6	78.7	20.7	80.7	5.3	82.1	12.2	78.3	2.1	83.2	9.4	82.1	8.8
Failed quality filter	0.2	0.0	0.1	0.0	0.1	0.0	0.1	0.0	0.2	0.0	0.2	0.0	0.1	0.0	0.1	0.0
Failed homopolymer filter	0.0	0.0	0.2	0.0	0.0	0.0	0.0	0.0	0.1	0.0	0.1	0.0	0.1	0.0	0.0	0.0
UniVec contaminants	0.4	0.0	0.7	0.3	0.8	0.9	0.5	0.2	0.6	0.4	0.6	0.2	0.5	0.3	0.6	0.4
rRNA	9.1	1.4	36.0	1.5	2.0	0.9	7.0	3.4	16.6	7.5	13.0	6.2	5.3	4.4	2.1	1.3
Reads used for alignment	84.7	2.2	26.4	2.8	75.8	21.0	73.1	7.6	64.6	19.5	64.5	7.2	77.2	14.0	79.3	9.8
Genome	82.2	1.9	22.7	2.3	69.0	26.7	58.0	9.5	15.7	5.3	14.2	3.3	56.1	31.7	53.7	33.6
miRNA sense	70.5	3.1	6.1	0.9	11.6	7.7	10.2	3.4	1.4	0.9	2.7	0.7	3.2	1.9	4.4	2.8
miRNAprecursor sense	0.2	0.0	0.1	0.0	0.0	0.0	0.0	0.0	0.0	0.0	0.0	0.0	0.0	0.0	0.0	0.0
tRNA sense	1.7	0.3	3.8	1.0	8.9	11.1	22.0	6.1	5.4	4.1	4.6	1.3	47.4	37.6	44.5	40.0
piRNA sense	1.5	0.5	1.2	0.2	4.9	3.3	1.1	0.4	0.1	0.0	0.1	0.0	0.1	0.1	0.1	0.1
Gencode sense	7.9	0.7	9.6	1.1	42.7	28.1	23.0	3.7	5.1	2.7	4.1	2.5	1.9	1.5	1.1	1.0
Not mapped to genome or transcriptome	2.5	0.2	3.8	0.5	6.7	5.8	15.1	5.3	48.9	19.7	50.3	10.1	21.2	17.8	25.6	23.9

^a^Number of million input reads obtained from sequencing of each sample and used in the alignment

### Small RNA biotype mapping

**Fig. 1:**
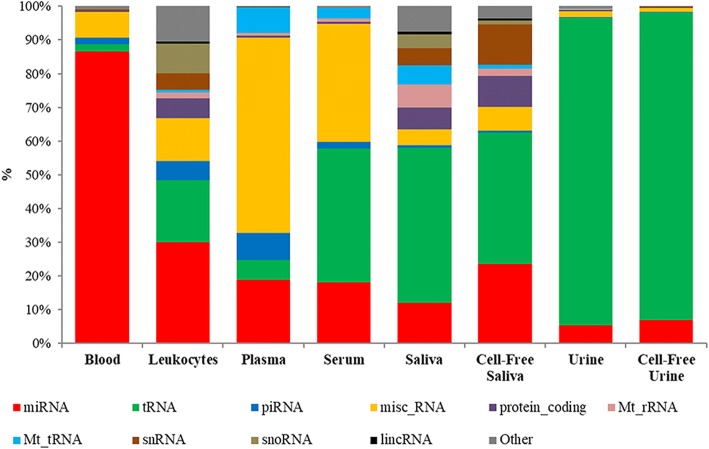
Relative biotype distribution among the various bodily fluids. The graph was generated using average percentage of biotype counts of each fluid. MicroRNAs are the largest biotype of blood, while tRNAs are the major biotype of urine. Saliva has the highest biotype diversity among all fluids

Reads that were mapped to the human genome were then mapped and classified to the various small RNA biotypes. The average total reads mapped to small RNA biotypes within each bodily fluid ranged from 1.3 to 12.8 million reads. Blood, plasma, cell-free urine and urine had more than 8 million reads mapped to biotypes (12.8, 9.7, 8.7 and 8.0 million reads, respectively). Serum, leukocytes, cell-free saliva and saliva had 5.4 million reads or less (5.4, 2.6, 1.3 and 1.3 million reads, respectively). The distribution of biotypes within each bodily fluid showed distinct patterns. Plasma had a high percentage of miscellaneous RNA (misc_RNA; 58.0 ± 39.4), while urine and cell-free urine had high amounts of tRNAs (91.3 ± 77.5% and 91.3 ± 90.3%, respectively). The other bodily fluids had a more diverse pattern with no single biotype exceeding 50% of the content. MicroRNAs represented more than 85% of blood biotypes, 25% of leukocytes, and 15–25% of plasma, serum and cell-free saliva. Saliva, cell-free urine and urine contained the lowest miRNA content (5.3–12.0%). Transfer RNA was the predominant biotype in urine and cell-free urine (> 90%), while serum, saliva and cell-free saliva contained moderate tRNA content (20–50%). Leukocytes had 18.4 ± 4.0 tRNA while plasma and blood contained the lowest tRNA fractions of 5.8 ± 2.5 and 2.1 ± 0.7, respectively. Finally, piRNAs represented less than 2% of the reads in blood, serum, saliva, cell-free saliva, urine and cell-free urine, while more than 5% of the reads were piRNAs in leukocytes and plasma. The percentages of the various biotypes in each bodily fluid are listed in Table 3 and illustrated in Fig. 1. The biotype distribution in each donor is illustrated in an additional file (Additional file 1: Figure S2) and shows a relatively similar pattern for each bodily fluid between the donors. However, in urine samples there was a difference in patterns between male and female donors.

**Table 3:** Percentage of biotype counts in the various bodily fluids

Biotype	Blood		Leukocytes		Plasma		Serum		Saliva		Cell-Free Saliva		Urine		Cell-Free Urine	
	Ave	SDEV	Ave	SDEV	Ave	SDEV	Ave	SDEV	Ave	SDEV	Ave	SDEV	Ave	SDEV	Ave	SDEV
^a^Million reads mapped to biotypes	12.8	1.9	2.6	0.1	9.7	6.2	5.4	1.6	1.3	0.8	1.3	0.5	8.0	5.9	8.7	7.6
miRNA	86.6	12.3	29.9	3.3	18.8	14.1	18.1	6.4	12.0	8.3	23.6	7.6	5.3	2.2	6.8	2.9
tRNA	2.1	0.7	18.4	4.0	5.8	2.5	39.7	16.9	46.0	39.2	39.0	11.6	91.3	77.5	91.3	90.3
piRNA	1.9	0.8	5.8	0.9	8.0	5.8	1.9	0.5	0.8	0.4	0.5	0.3	0.3	0.2	0.2	0.2
misc_RNA	7.7	1.5	12.7	2.6	58.0	39.4	35.1	10.3	4.6	2.6	7.1	2.0	1.7	1.7	1.1	0.8
protein_coding	0.4	0.1	5.8	0.6	0.5	0.4	0.6	0.2	6.6	3.9	9.2	7.0	0.4	0.3	0.2	0.1
Mt_rRNA	0.0	0.0	1.9	0.4	0.9	0.7	0.9	0.4	6.8	4.3	2.1	1.8	0.3	0.1	0.0	0.0
Mt_tRNA	0.1	0.0	0.7	0.1	7.7	5.1	3.1	1.3	5.5	2.4	1.2	1.1	0.2	0.1	0.1	0.0
snRNA	0.2	0.0	4.9	0.4	0.0	0.0	0.1	0.1	5.2	5.4	11.9	11.0	0.1	0.1	0.0	0.0
snoRNA	0.3	0.0	8.7	0.8	0.0	0.0	0.1	0.1	4.1	2.6	1.3	1.4	0.1	0.1	0.0	0.0
lincRNA	0.0	0.0	0.7	0.1	0.0	0.0	0.0	0.0	0.7	0.4	0.5	0.3	0.0	0.0	0.0	0.0
Others	0.6	0.1	10.5	1.0	0.2	0.1	0.3	0.1	7.6	4.8	3.7	2.5	0.3	0.2	0.1	0.1

^a^Number of million reads mapped to biotypes

### miRNA

**Fig. 2:**
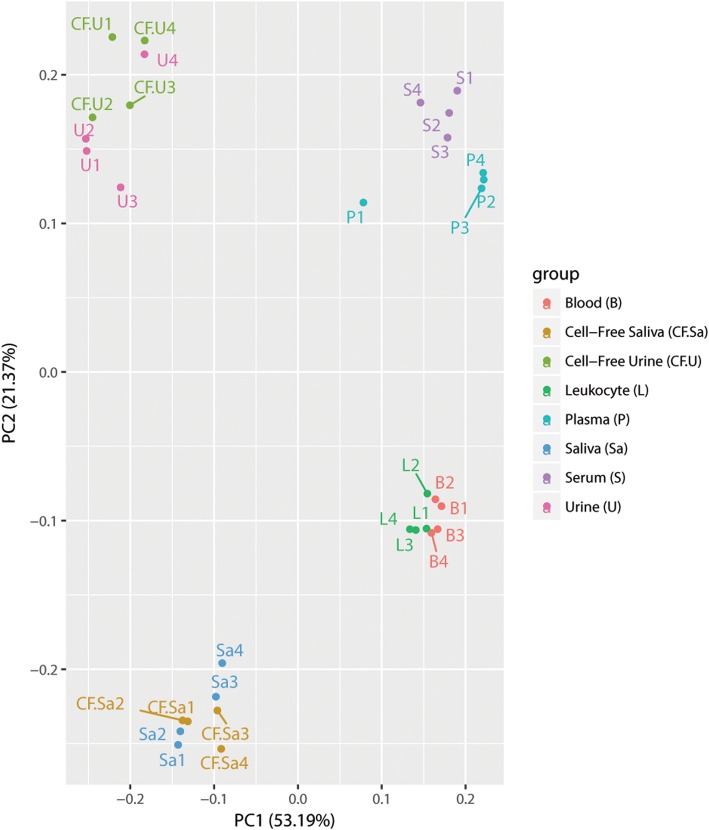
Principal component analysis of the most variable miRNAs in each bodily fluid. Analysis of the most variable 50 miRNAs was calculated based on TMM-normalized miRNA counts. Four pairs of fluids show close clustering: blood/leukocyte, plasma/serum, saliva/cell-free saliva and urine/cell-free urine

**Fig. 3:**
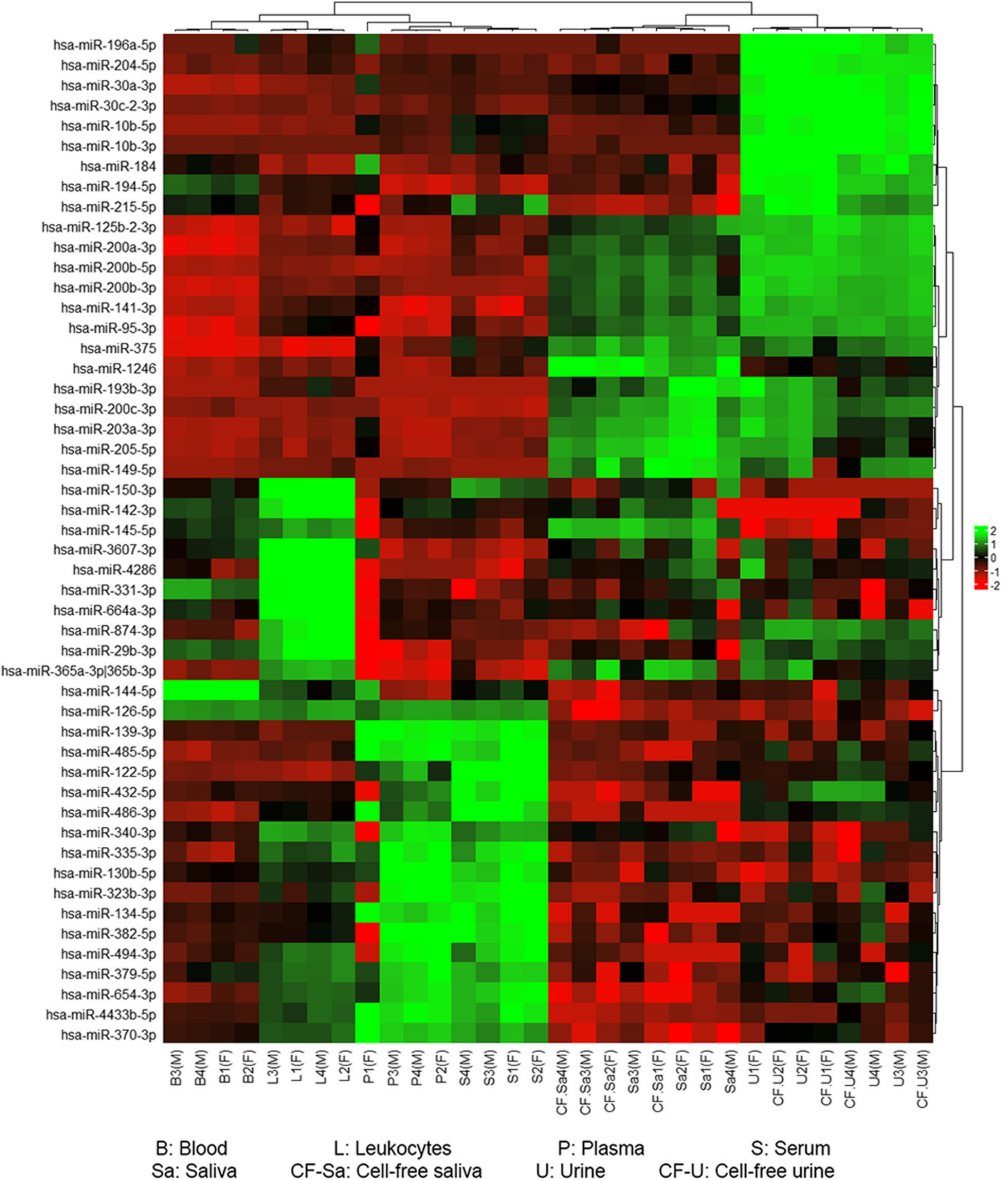
Heatmap clustering of the most variable miRNAs in each of the bodily fluids. The sex of the sample donor is indicated as (F) for female donors or (M) for male donors. The analysis was generated using Z scores of the most variable 50 miRNAs. The dendrogram shows distinct clustering of invasive fluids (blood, leukocyte, plasma and serum) and non-invasive fluids (saliva, cell-free saliva, urine and cell-free urine)

The most variable 50 miRNAs were calculated based on TMM-normalized miRNA counts at a CPM corresponding to a minimum of 5 counts in a library to achieve a high confidence level. These miRNAs were then used for PCA which showed the separation of various bodily fluids based on their miRNA expression (Fig. 2). The analysis revealed closeness between saliva and cell-free saliva samples and between urine and cell-free urine samples. Close clustering was seen between blood and leukocytes, and between plasma and serum. The data disclosed consistent biological origin and miRNA expression-based separation of bodily fluid profiles. The Z scores of the most variable 50 miRNAs were used to generate a heatmap that illustrated the pattern of expression as well as the relationship between samples (Fig. 3). It clustered bodily fluids based on their biology. The dendrogram showed that invasive bodily fluids (blood, leukocytes, plasma and serum) branched apart from non-invasive fluids (saliva, cell-free saliva, urine and cell-free urine). Furthermore, it showed that cell-depletion from saliva or urine did not have a major effect on their clustering. A large set of miRNAs appeared to be highly abundant or severely depleted in the various bodily fluids. Particularly, urine and cell-free urine as well as plasma and serum had different sets of upregulated miRNAs.

**Fig. 4:**
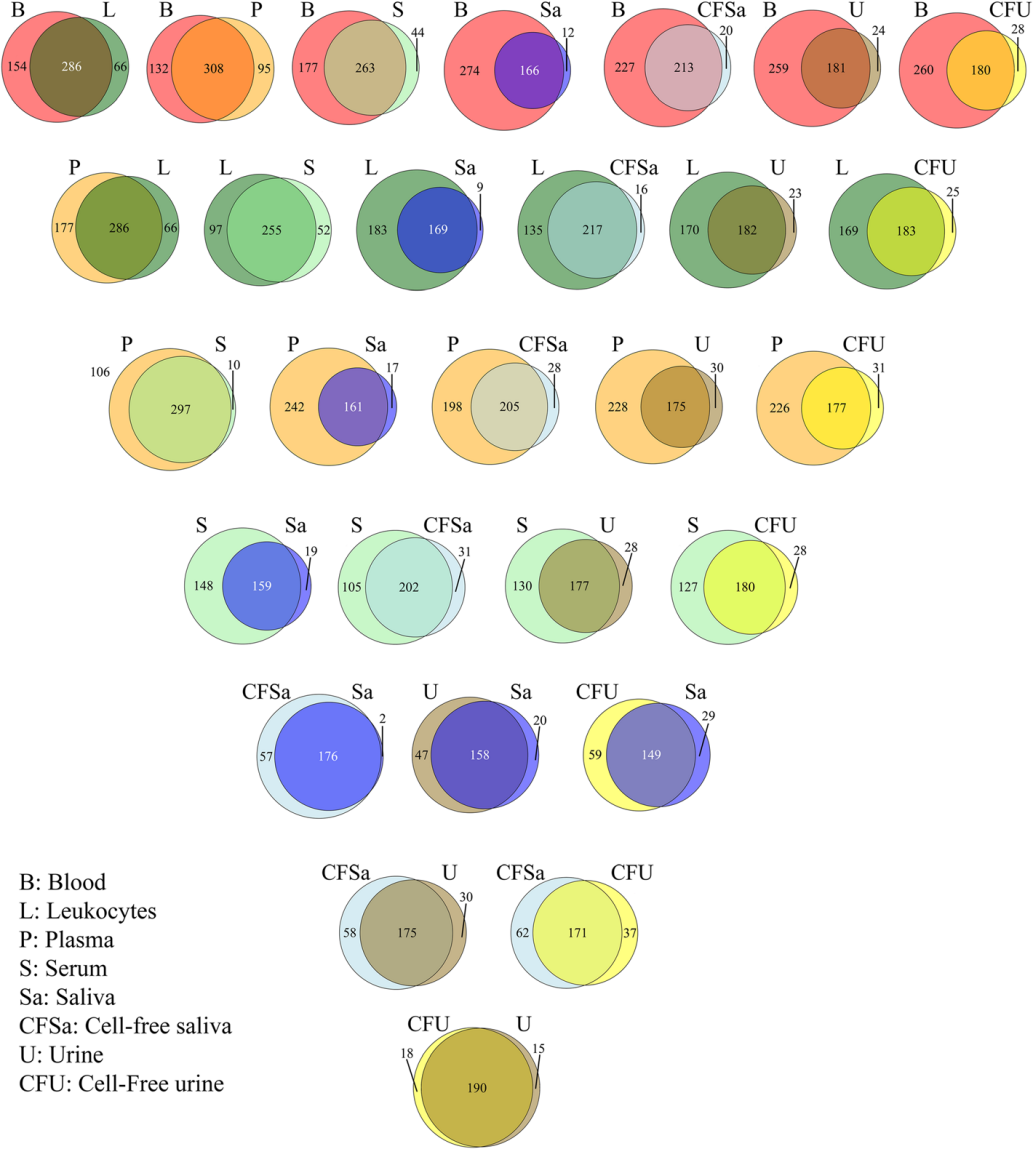
Overlap of miRNA content between various bodily fluids. MicroRNAs of each fluid were filtered to keep molecules that have a minimum of 5 counts in 3 or more individuals. The highest overlap is seen between fluids within the same category: invasive fluids (blood, leukocyte, plasma and serum) or non-invasive fluids (saliva, cell-free saliva, urine and cell-free urine)

**Fig. 5:**
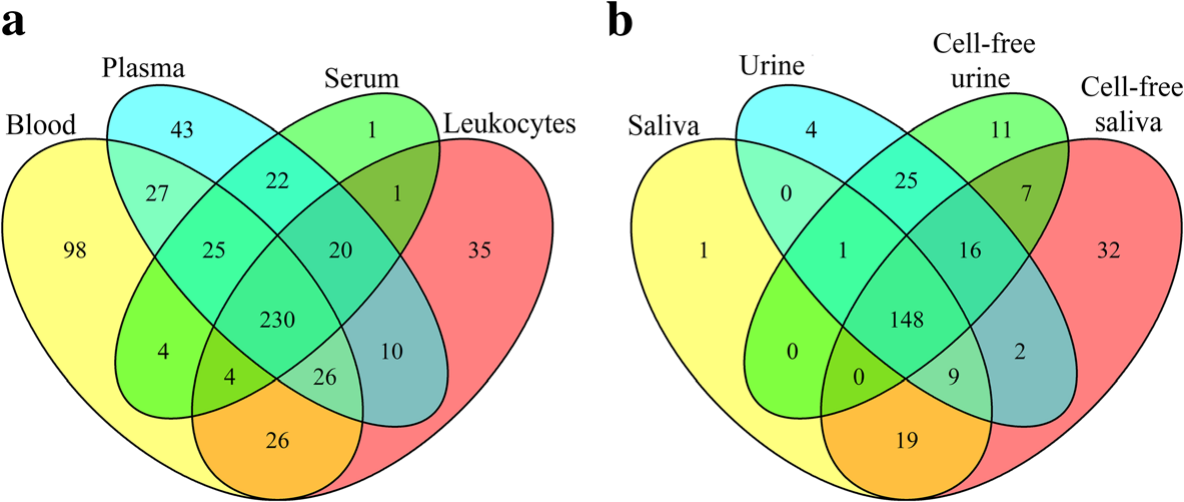
Venn diagram showing the overlap between (**a**) invasive and (**b**) non-invasive bodily fluids. Only miRNAs that are present at a minimum of 5 counts in 3 or more individuals were included in the comparison. Invasive fluids have higher number of shared and unique miRNAs compared to non-invasive fluids

Bodily fluids can be classified into two groups based on their collection procedures: invasive (blood, leukocytes, plasma and serum) and non-invasive (saliva and urine). Variations can be seen in the detectable number of miRNAs, at a minimum of 5 counts in 3 or more individuals, between the two groups. The range of detected miRNAs from invasive fluids was 307–440 while the range from the non-invasive fluids was 178–233. Blood had the largest number of detected miRNAs and saliva had the lowest. Plasma contained more miRNAs compared to serum. Similarly, more miRNAs were detected from the cell-free preparation of saliva than saliva. Almost no differences could be seen between urine and cell-free urine (Table 4). The overlap between detected miRNAs in the different bodily fluids was illustrated in Venn diagrams (Fig. 4). About 97% of serum miRNAs were shared with plasma. More than 90% of miRNAs in saliva and cell-free saliva were shared with blood, leukocytes and plasma. Saliva had 98.9% of its miRNA identical to cell-free saliva, while the latter had only 75.5% of its miRNAs overlapped with saliva. About 85–91% of urine and cell-free urine miRNAs were overlapped with blood, leukocytes, plasma and serum. Cell-free urine had 91.3% similarity with urine, while urine had 92.7% similarity to cell-free urine. Saliva and urine shared more than 77% of their miRNAs. In addition, Venn diagrams were used to demonstrate the overlap between invasive and non-invasive bodily fluids (Fig. 5). The invasive fluids had 230 common miRNAs (Additional file 2: Table S1). Blood had 98 unique miRNAs, which was 2 to 3-fold higher than plasma and leukocytes. In contrast, non-invasive fluids had 148 common miRNAs (Additional file 3: Table S2), and had lower numbers of unique miRNAs. The non-invasive fluids shared 144 common miRNAs with blood (Additional file 4: Table S3), while the latter had 209 unique miRNAs that were absent from all non-invasive fluids (Additional file 5: Figure S1). Common miRNAs between all fluids were 139 (Additional file 6: Table S4).

**Table 4:** Detected number of miRNAs in the various bodily fluids

Bodily fluid	Blood	Leukocytes	Plasma	Serum	Saliva	Cell-Free Saliva	Urine	Cell-Free Urine
Number of miRNAs	440	352	403	307	178	233	205	208

Only miRNAs that are present at a minimum of 5 counts in 3 or more individuals were considered detectable

**Fig. 6:**
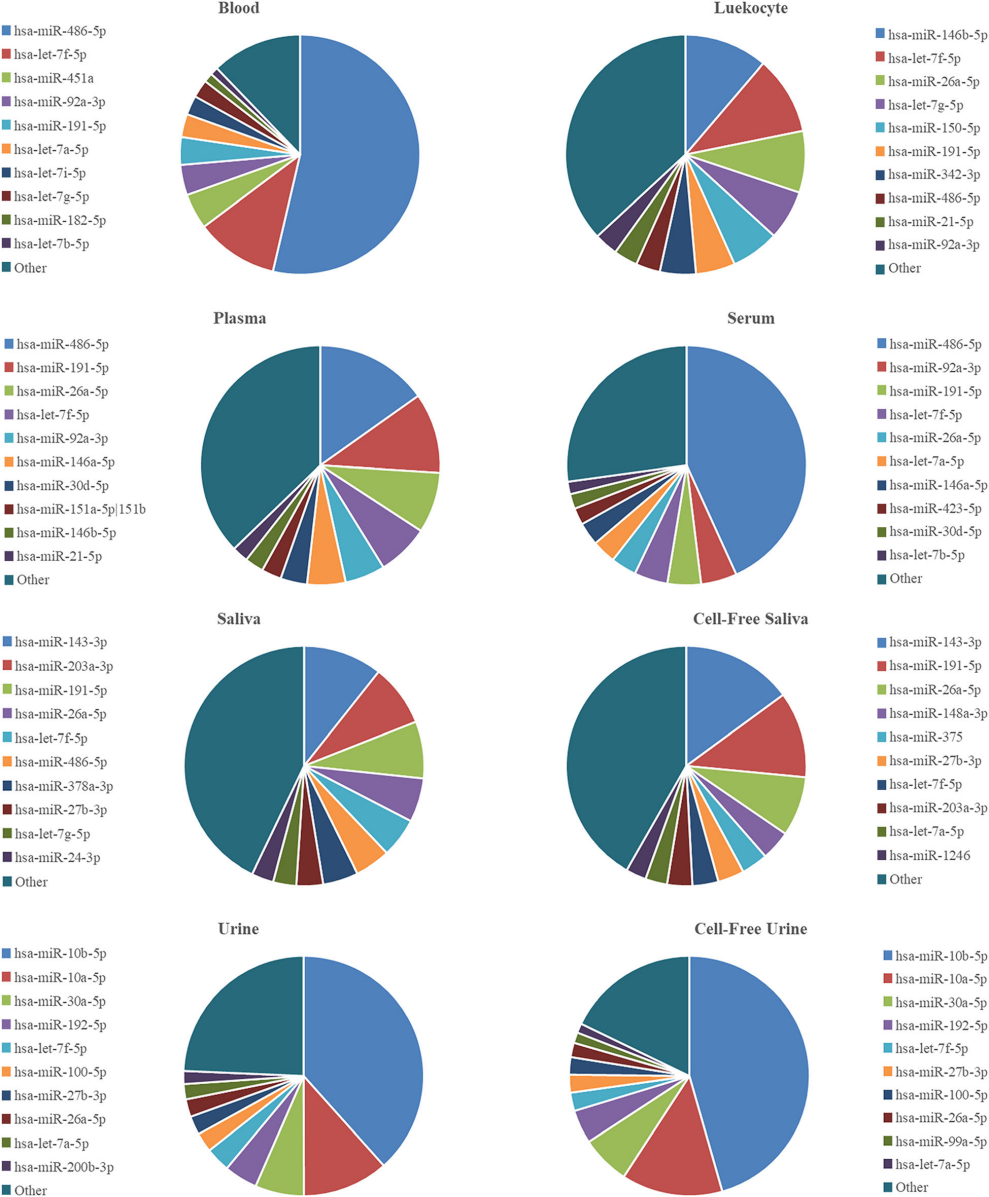
Top 10 most abundant miRNAs relative to all miRNA counts in each fluid. The 10 miRNAs that have the highest read counts in each fluid were illustrated relative to the total miRNA read counts of the fluid. Counts of the remaining miRNAs were summed up and illustrated as “other”

**Fig. 7:**
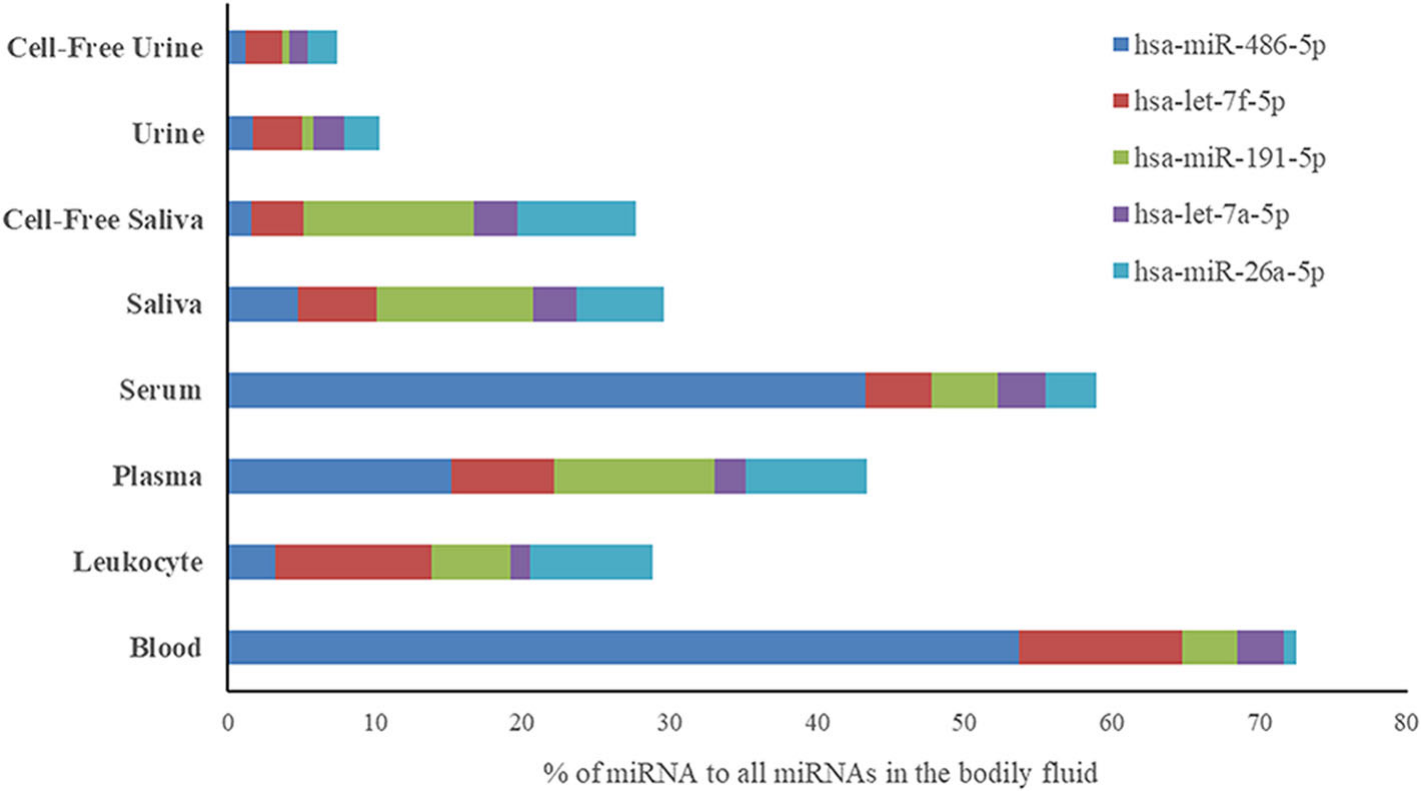
Relative abundance of the top 5 common miRNAs between the different fluids. Counts of the 5 common miRNAs are presented relative to the total miRNA counts of each fluid. The five common miRNAs represent a large fraction of the invasive fluids with the highest percentage in blood. They represent lower fractions in the non-invasive fluids

We ran a detailed analysis on the miRNA composition of each bodily fluid. We looked at the 20 most abundant miRNAs and calculated their fractions of the total miRNA content of each sample (Table 5). These 20 most abundant miRNAs covered about 74 to 94% of all miRNA counts. They represented 94 and 91% of blood and cell-free urine, respectively. They were only 75% of all miRNA counts in saliva and cell-free saliva, while representing 80–88% of the rest of the other bodily fluids. The deep analysis of miRNAs revealed that 2 specific miRNAs were dominating the counts of 4 bodily fluids. Hsa-miR-486-5p made up 53.6 ± 1.9% and 43.2 ± 13.2% of miRNA counts of blood and serum, respectively, while hsa-miR-10b-5p represented 38.4 ± 8.3% and 45.6 ± 5.6% of miRNA counts of urine and cell-free urine, respectively. Other miRNAs that represented 10–20% of total miRNA counts included let-7f-5p (11.11 ± 1.12%) in blood, miR-146b-5p (11.23 ± 1.85) and let-7f-5p (10.58 ± 1.33) in leukocytes, miR-486-5p (15.16 ± 1.63) and miR-191-5p (10.84 ± 0.25) in plasma, miR-143-3p (10.65 ± 4.16) in saliva, miR-143-3p (14.92 ± 4.47) and miR-191-5p (11.59 ± 1.64) in cell-free saliva, and miR-10a-5p in urine (11.56 ± 2.67) and cell-free urine (13.6 ± 0.97). The proportions of the top 10 most abundant miRNAs in each fluid are illustrated in Fig. 6. Among the top 20 most abundant miRNAs of each fluid, 5 miRNAs were found common to all fluids. These include: hsa-let-7a-5p, hsa-let-7f-5p, hsa-miR-191-5p, hsa-miR-26a-5p and hsa-miR-486-5p. These five miRNAs represent more than 50% of blood and serum miRNA counts, 25 to 45% of plasma and saliva (total and cell-free) and less than 11% of urine and cell-free urine miRNA counts (Fig. 7).

**Table 5:** Twenty most abundant miRNAs detected in each bodily fluid

#	Blood	Leukocytes	Plasma	Serum	Saliva	Cell-Free Saliva	Urine	Cell-Free Urine
1	hsa-miR-486-5p (53.64 ± 1.89)	hsa-miR-146b-5p (11.23 ± 1.85)	hsa-miR-486-5p (15.16 ± 1.63)	hsa-miR-486-5p (43.22 ± 13.23)	hsa-miR-143-3p (10.65 ± 4.16)	hsa-miR-143-3p (14.92 ± 4.47)	hsa-miR-10b-5p (38.4 ± 8.33)	hsa-miR-10b-5p (45.58 ± 5.62)
2	hsa-let-7f-5p (11.11 ± 1.12)	hsa-let-7f-5p (10.58 ± 1.33)	hsa-miR-191-5p (10.84 ± 0.25)	hsa-miR-92a-3p (4.85 ± 0.48)	hsa-miR-203a-3p (8.36 ± 5.89)	hsa-miR-191-5p (11.59 ± 1.64)	hsa-miR-10a-5p (11.56 ± 2.67)	hsa-miR-10a-5p (13.6 ± 0.97)
3	hsa-miR-451a (4.79 ± 1.08)	hsa-miR-26a-5p (8.32 ± 0.33)	hsa-miR-26a-5p (8.21 ± 0.77)	hsa-miR-191-5p (4.51 ± 1.77)	hsa-miR-191-5p (7.66 ± 4.8)	hsa-miR-26a-5p (8.06 ± 0.69)	hsa-miR-30a-5p (6.58 ± 1.5)	hsa-miR-30a-5p (6.59 ± 1.95)
4	hsa-miR-92a-3p (4.09 ± 0.55)	hsa-let-7 g-5p (6.68 ± 0.97)	hsa-let-7f-5p (6.99 ± 0.7)	hsa-let-7f-5p (4.5 ± 1.5)	hsa-miR-26a-5p (5.94 ± 4.11)	hsa-miR-148a-3p (4.01 ± 0.63)	hsa-miR-192-5p (4.5 ± 2.12)	hsa-miR-192-5p (4.53 ± 2.42)
5	hsa-miR-191-5p (3.74 ± 0.64)	hsa-miR-150-5p (6.48 ± 2.23)	hsa-miR-92a-3p (5.41 ± 0.09)	hsa-miR-26a-5p (3.45 ± 1.74)	hsa-let-7f-5p (5.32 ± 2)	hsa-miR-375 (3.56 ± 1.92)	hsa-let-7f-5p (3.32 ± 0.77)	hsa-let-7f-5p (2.47 ± 0.51)
6	hsa-let-7a-5p (3.12 ± 0.86)	hsa-miR-191-5p (5.3 ± 0.68)	hsa-miR-146a-5p (5.17 ± 0.13)	hsa-let-7a-5p (3.21 ± 0.87)	hsa-miR-486-5p (4.77 ± 3.31)	hsa-miR-27b-3p (3.55 ± 1.66)	hsa-miR-100-5p (2.61 ± 0.64)	hsa-miR-27b-3p (2.41 ± 0.76)
7	hsa-let-7i-5p (2.6 ± 0.09)	hsa-miR-342-3p (4.84 ± 2.52)	hsa-miR-30d-5p (3.58 ± 0.43)	hsa-miR-146a-5p (3.11 ± 1.21)	hsa-miR-378a-3p (4.75 ± 7.51)	hsa-let-7f-5p (3.49 ± 0.57)	hsa-miR-27b-3p (2.52 ± 1.13)	hsa-miR-100-5p (2.36 ± 0.57)
8	hsa-let-7 g-5p (2.42 ± 0.12)	hsa-miR-486-5p (3.27 ± 1.37)	hsa-miR-151a-5p|hsa-miR-151b (2.67 ± 0.09)	hsa-miR-423-5p (2.27 ± 0.45)	hsa-miR-27b-3p (3.59 ± 1.13)	hsa-miR-203a-3p (3.39 ± 1.96)	hsa-miR-26a-5p (2.38 ± 0.41)	hsa-miR-26a-5p (1.94 ± 0.34)
9	hsa-miR-182-5p (1.25 ± 0.25)	hsa-miR-21-5p (3.21 ± 0.52)	hsa-miR-146b-5p (2.54 ± 0.16)	hsa-miR-30d-5p (2.01 ± 0.51)	hsa-let-7 g-5p (3.11 ± 1.36)	hsa-let-7a-5p (2.94 ± 0.25)	hsa-let-7a-5p (2.06 ± 0.91)	hsa-miR-99a-5p (1.43 ± 0.36)
10	hsa-let-7b-5p (1.07 ± 0.26)	hsa-miR-92a-3p (3.19 ± 0.23)	hsa-miR-21-5p (2.18 ± 0.17)	hsa-let-7b-5p (1.69 ± 0.25)	hsa-miR-24-3p (2.97 ± 1.86)	hsa-miR-1246 (2.74 ± 2.33)	hsa-miR-200b-3p (1.75 ± 0.77)	hsa-let-7a-5p (1.27 ± 0.16)
11	hsa-miR-185-5p (1.01 ± 0.09)	hsa-miR-146a-5p (3.13 ± 0.77)	hsa-let-7a-5p (2.12 ± 0.26)	hsa-miR-122-5p (1.32 ± 1.46)	hsa-let-7a-5p (2.93 ± 1.31)	hsa-miR-92a-3p (2.45 ± 0.28)	hsa-miR-486-5p (1.71 ± 1.05)	hsa-miR-486-5p (1.26 ± 0.95)
12	hsa-miR-16-5p (0.95 ± 0.21)	hsa-miR-143-3p (2.49 ± 1.42)	hsa-miR-423-5p (2.11 ± 0.19)	hsa-miR-151a-5p|hsa-miR-151b (1.29 ± 0.38)	hsa-miR-375 (2.31 ± 1.17)	hsa-miR-423-5p (1.74 ± 1.49)	hsa-miR-99a-5p (1.65 ± 0.43)	hsa-let-7b-5p (1.21 ± 0.34)
13	hsa-miR-26a-5p (0.86 ± 0.11)	hsa-let-7i-5p (1.86 ± 0.21)	hsa-miR-423-3p (2.03 ± 0.17)	hsa-miR-451a (1.23 ± 0.6)	hsa-miR-148a-3p (2 ± 0.86)	hsa-let-7b-5p (1.72 ± 0.79)	hsa-miR-148a-3p (1.48 ± 0.6)	hsa-miR-200b-3p (1.13 ± 0.25)
14	hsa-miR-25-3p (0.64 ± 0.08)	hsa-miR-30d-5p (1.78 ± 0.3)	hsa-miR-99b-5p (1.98 ± 0.38)	hsa-miR-146b-5p (1.09 ± 0.38)	hsa-miR-21-5p (1.85 ± 1.27)	hsa-miR-30e-5p (1.71 ± 0.26)	hsa-miR-203a-3p (1.36 ± 1.7)	hsa-miR-148a-3p (1.07 ± 0.36)
15	hsa-miR-183-5p (0.62 ± 0.05)	hsa-miR-451a (1.4 ± 0.59)	hsa-miR-151a-3p (1.94 ± 0.33)	hsa-miR-151a-3p (1.05 ± 0.23)	hsa-miR-205-5p (1.62 ± 0.92)	hsa-miR-99a-5p (1.71 ± 0.82)	hsa-miR-21-5p (1.17 ± 0.92)	hsa-miR-30d-5p (0.93 ± 0.32)
16	hsa-miR-181a-5p (0.58 ± 0.15)	hsa-let-7a-5p (1.38 ± 0.4)	hsa-let-7i-5p (1.88 ± 0.2)	hsa-miR-320a (0.92 ± 0.36)	hsa-miR-320a (1.44 ± 1.94)	hsa-miR-24-3p (1.64 ± 0.11)	hsa-let-7b-5p (1.17 ± 0.23)	hsa-miR-99b-5p (0.86 ± 0.44)
17	hsa-miR-151a-5p|hsa-miR-151b (0.53 ± 0.05)	hsa-miR-181a-5p (1.36 ± 0.27)	hsa-miR-127-3p (1.36 ± 0.21)	hsa-miR-10a-5p (0.89 ± 0.42)	hsa-miR-99a-5p (1.4 ± 0.16)	hsa-miR-486-5p (1.63 ± 0.53)	hsa-miR-30d-5p (1.04 ± 0.28)	hsa-miR-423-5p (0.7 ± 0.17)
18	hsa-miR-101-3p (0.42 ± 0.1)	hsa-miR-223-3p (1.23 ± 0.71)	hsa-let-7 g-5p (1.34 ± 0.19)	hsa-let-7i-5p (0.87 ± 0.14)	hsa-let-7i-5p (1.4 ± 0.33)	hsa-miR-23a-3p (1.52 ± 0.38)	hsa-miR-99b-5p (0.88 ± 0.34)	hsa-miR-151a-5p|hsa-miR-151b (0.55 ± 0.07)
19	hsa-miR-30d-5p (0.4 ± 0.09)	hsa-miR-30e-5p (1.16 ± 0.09)	hsa-miR-222-3p (1.2 ± 0.32)	hsa-miR-99b-5p (0.8 ± 0.3)	hsa-miR-92a-3p (1.39 ± 0.41)	hsa-miR-200b-3p (1.26 ± 0.69)	hsa-miR-191-5p (0.8 ± 0.41)	hsa-miR-30a-3p (0.53 ± 0.07)
20	hsa-miR-30e-5p (0.36 ± 0.07)	hsa-miR-101-3p (1.07 ± 0.16)	hsa-miR-10a-5p (1.18 ± 0.23)	hsa-miR-22-3p (0.8 ± 0.19)	hsa-let-7b-5p (1.22 ± 0.33)	hsa-miR-223-3p (1.23 ± 0.19)	hsa-miR-423-5p (0.65 ± 0.22)	hsa-miR-191-5p (0.46 ± 0.09)

MicroRNAs are arranged in a descending order from highest to lowest. Percentage of miRNA to all miRNAs in the bodily fluid is shown between brackets. None of the unique miRNAs of each bodily fluid were found among its top 20 miRNAs

Analysis of unique and novel miRNAs are valuable in evaluating the usefulness of using a specific specimen as a source of information. We ran the analysis of unique miRNAs in our study using 3 comparison groups: all fluids, invasive fluids and non-invasive fluids. The number of unique miRNAs detected in each comparison group are listed in Table 6. Blood, leukocytes and plasma had significantly higher numbers of unique miRNAs compared to the rest of fluids. Blood had the highest number of unique molecules (94 miRNAs), while plasma and leukocytes had 42 and 30 unique miRNAs, respectively. The comparison within the invasive group showed a similar trend. The comparison within the non-invasive group showed that cell-free saliva had the most unique miRNAs (32 miRNAs) followed by cell-free urine (11 miRNAs). Saliva and urine had minimal numbers of unique miRNAs compared to their cell-free preparations. The list of unique miRNAs from the three comparison groups are listed in three additional files (Additional file 7: Table S5, Additional file 8: Table S6, Additional file 9: Table S7).

**Table 6:** Number of unique miRNAs in each bodily fluid at a minimum of 5 counts

Bodily fluid	All	Invasive	Non-invasive
Blood	94	96	Not compared
Leukocytes	30	35	Not compared
Plasma	42	43	Not compared
Serum	1	1	Not compared
Saliva	1	Not compared	1
Cell-Free Saliva	3	Not compared	32
Urine	1	Not compared	4
Cell-Free Urine	3	Not compared	11

Three comparison groups were used: “All” indicating unique miRNA among all fluids, “invasive” for comparison within the invasive fluids (blood, leukocytes, serum and plasma), and “non-invasive” for comparison within the non-invasive fluids (saliva, cell-free saliva, urine and cell-free urine)

All bodily fluids were analyzed for their novel miRNA candidates using miRDeep2. Signal-to-noise ratio of more than 10 was used to select for miRDeep2 score cutoff [68]. In bodily fluids where signal-to-noise ratio was less than 10 (saliva, urine and cell-free urine), we selected the score cutoff that corresponds to the highest signal-to-noise ratio (Table 7). Invasive bodily fluids had higher numbers of novel miRNA candidates than non-invasive fluids. The highest number of novel miRNA candidates was observed in plasma and blood, with 50 and 48 candidates, respectively. Serum had 20 novel candidates while leukocytes had 16 candidates. All the non-invasive fluids had 7 or less novel candidates. Sequences of novel miRNA candidates were matched to the miRCarta database (v1.0) of newly predicted human miRNAs [69]. More than 50% of blood and leukocytes novel miRNA candidates were present in miRCarta (66 and 56%, respectively). Plasma and serum had less miRNA candidates matching miRCarta database (30 and 45%, respectively). No miRCarta matches were found for the novel miRNA candidates of the non-invasive fluids. An additional file containing the list of novel miRNA candidates in each bodily fluid and their sequences, as well as the matching results to the miRCarta database is provided (Additional file 10: Table S8).

**Table 7:** Number of novel miRNA candidates in the different bodily fluids

Bodily fluid	Signal-to-noise	miRDeep2 score	Number of novel miRNA candidates	Number of candidates present in miRCarta database
Blood	15.9	5	48	32
Leukocytes	11.5	5	16	9
Plasma	17	5	50	15
Serum	17.1	5	20	9
Saliva	4.8^a^	5	4^a^	0
Cell-Free Saliva	10.5	5	7	0
Urine	7.6^a^	5	5^a^	0
Cell-Free Urine	7.7^a^	5	5^a^	0

^a^Signal-to-noise ratio is below the minimum accepted cutoff (10). The highest signal-to-noise ratio was used. However, predicted novel miRNAs at this value might not be real. Predicted novel miRNAs with a non-significant p-value of the RNA minimum free energy of folding randomization test (Randfold) have not been counted

### tRNA

Mapped tRNAs represented tDRs down to 18 nucleotides. The predominant tRNA fragments in all the bodily fluids was tRNA^Gly^. This tRNA composed 86.5 and 87.6% of the total tRNA content in urine and cell-free urine, respectively. For the remaining bodily fluids, it made up 72.0 to 84.1% of the total tRNA content. The second most abundant tRNA was tRNA^Glu^, with a range of 6.7 to 21.4% of the tRNA content. Further analysis of tRNAs by tDRmapper to look at the exact tDRs composition showed that tRNA^Gly-GCC^ and tRNA^Glu-CTC^ were the predominant fragments in all fluids. All samples, regardless of the fluid origin, shared similar tDRs composition. The quantification and coverage of the top 50 tDRs in each fluid are presented in Additional files 11 and 12. None of the remaining tRNAs in any of the fluids exceeded 3.1% of the tRNA content (Table 8). For blood, plasma, saliva and cell-free saliva, there was a higher diversity of tRNAs that represented 1% or more of the total tRNA content of the sample (5 tRNAs or more). However, for leukocytes, serum, urine and cell-free urine, there was a lower diversity (3 to 4 tRNAs).

**Table 8:** List of tRNAs that represents ≥1% of all tRNA counts in each bodily fluid

Blood	Leukocyte	Plasma	Serum	Saliva	Cell-Free Saliva	Urine	Cell-Free Urine
Gly (84.1 ± 1.8)	Gly (77.8 ± 1.9)	Gly (79.9 ± 12.7)	Gly (73.5 ± 8.2)	Gly (83.7 ± 6.6)	Gly (72.0 ± 7.4)	Gly (86.5 ± 6.4)	Gly (87.6 ± 6.0)
Glu (6.7 ± 0.5)	Glu (11.8 ± 2.6)	Glu (9.5 ± 6.0)	Glu (21.4 ± 7.5)	Glu (7.9 ± 4.5)	Glu (15.4 ± 2.6)	Glu (8.0 ± 4.2)	Glu (8.4 ± 3.9)
Lys (2.1 ± 0.4)	Lys (3.1 ± 0.7)	SeC (1.8 ± 1.5)	Val (1.5 ± 0.2)	Val (2.1 ± 0.5)	Lys (2.8 ± 0.9)	Val (1.8 ± 1.9)	Lys (1.5 ± 0.5)
SeC (1.0 ± 0.3)	Val (2.8 ± 0.4)	His (1.5 ± 1.2)	Lys (1.3 ± 0.5)	Lys (1.6 ± 0.5)	Ala (2.6 ± 2.7)	Lys (1.6 ± 0.7)	
His (1.2 ± 0.1)		Arg (1.2 ± 1.0)		His (1.0 ± 0.5)	Val (1.6 ± 0.2)		
Val (1.2 ± 0.2)		Gln (1.2 ± 0.8)			Asp (1.5 ± 0.7)		
		Lys (1.1 ± 0.6)					
		Pro (1.0 ± 0.9)					

Average percentage and standard deviation for each tRNA relative to the total tRNA content of the bodily fluid is included between the brackets

**Fig. 8:**
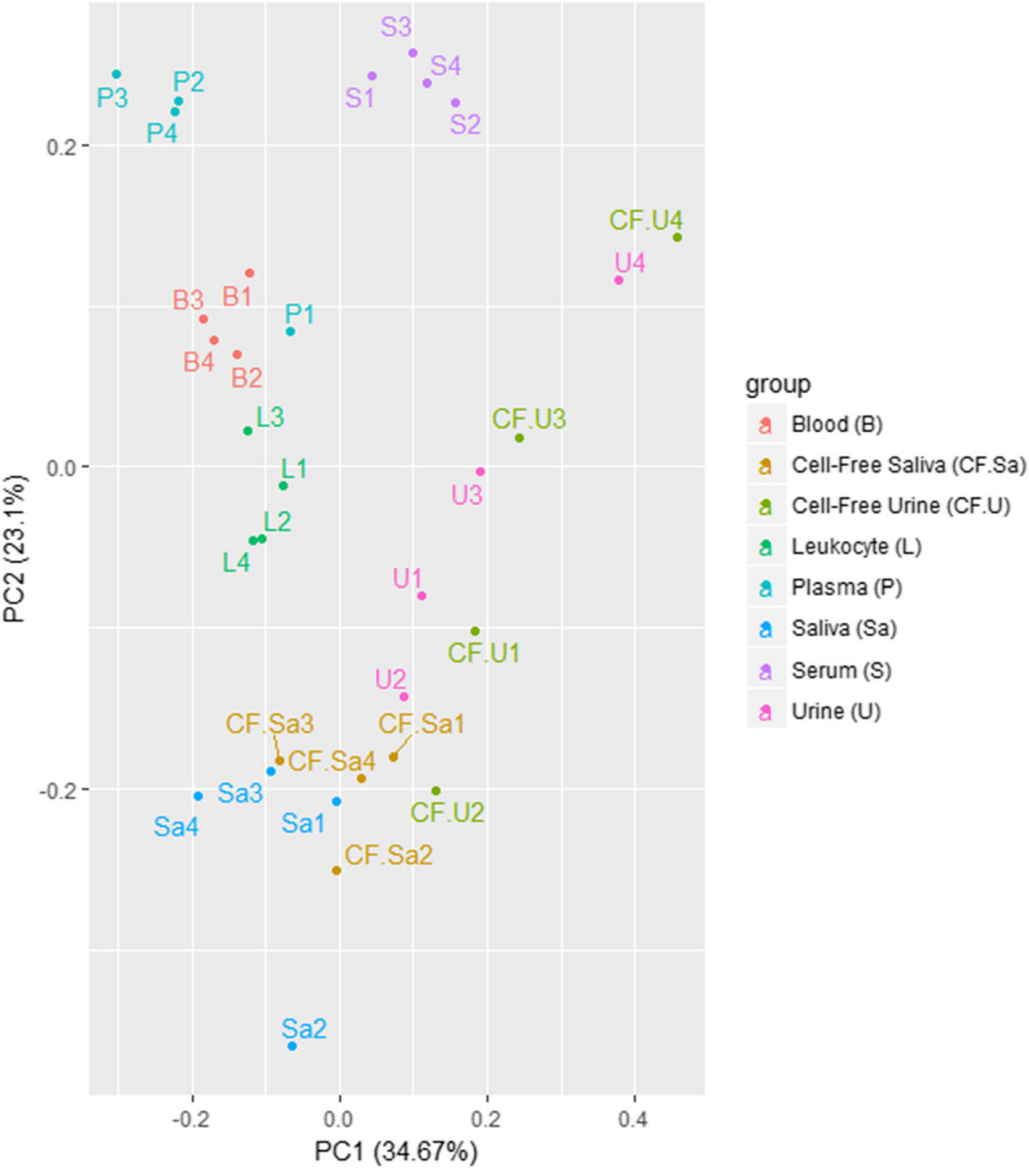
Principal component analysis of tRNAs in each bodily fluid. The plot was generated based on TMM-normalized tRNA counts. Samples of the same origin clustered closer to each other. However, urine samples are more dispersed from each other

**Fig. 9:**
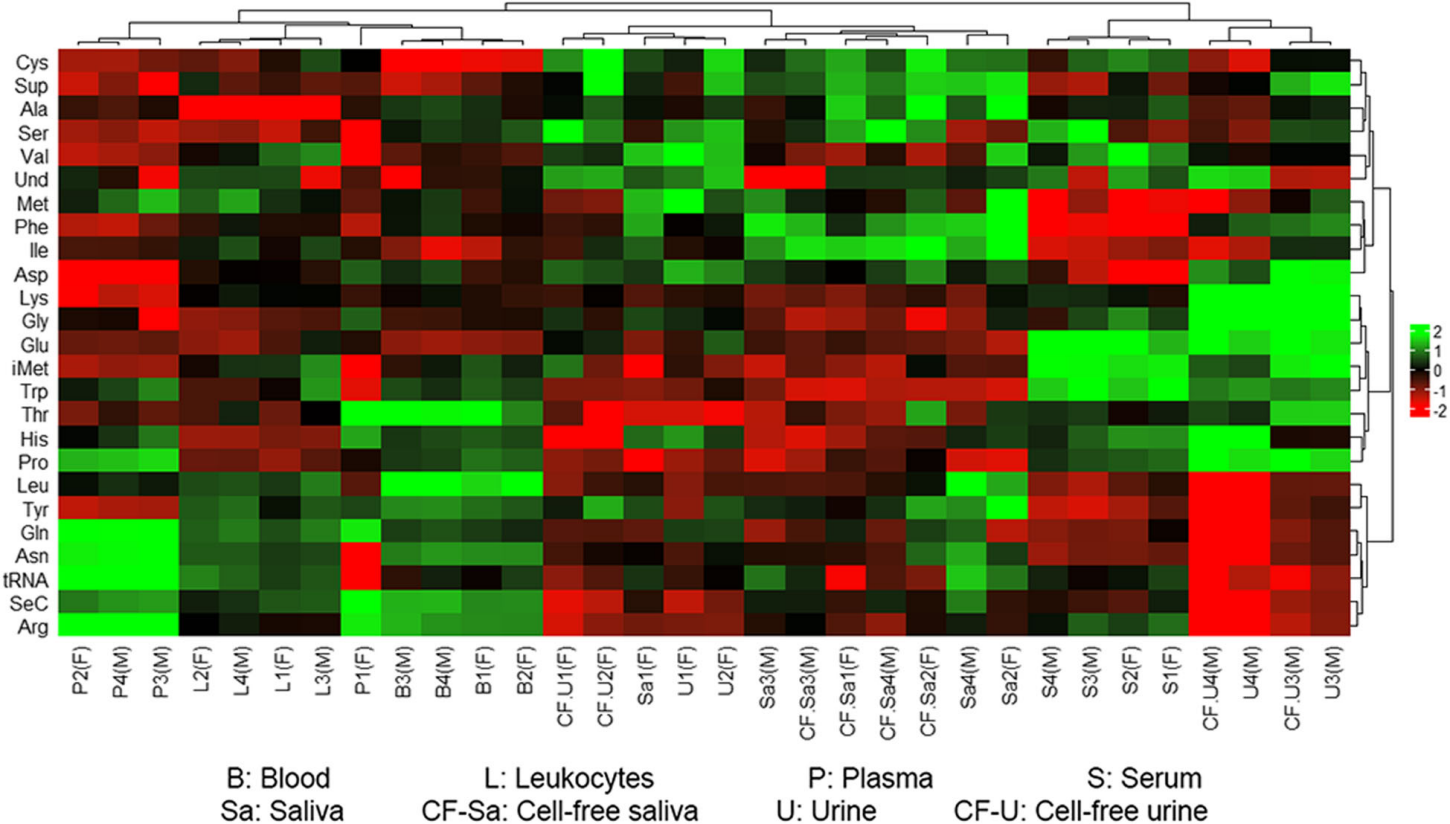
Heatmap clustering of tRNAs in the various bodily fluids. The sex of sample donor is indicated as (F) for female donors or (M) for male donors. The analysis was generated using Z scores of TMM-normalized tRNA counts. The dendrogram shows distant clustering of urine samples (total and cell-free) based on sex. Male urine samples (total and cell-free) clustered close to serum while female urine samples (total and cell-free) are clustered with saliva

Read counts of tRNAs were normalized using TMM at a CPM corresponding to a minimum of 5 counts in a library. The normalized reads were then used to generate principal component analysis which showed the separation of various bodily fluids based on their tRNA levels (Fig. 8). The analysis revealed closeness between saliva and cell-free saliva as well as closeness between the invasive fluids. However, urine and cell-free urine were dispersed between both saliva and serum. The Z scores of these tRNAs were used to generate a heatmap that indicates the levels of various tRNAs in the different samples (Fig. 9). Serum and the male urine/cell-free urine samples showed distant clustering from the rest of samples. The female urine/cell-free urine clustered with saliva/cell-free saliva. Blood, leukocytes and plasma showed similar clustering. The data shows clustering patterns based on sample biology and no difference between cell-depleted and non-depleted conditions.

### piRNA

All bodily fluids had piR-016658 at different levels. The highest levels were seen in blood and serum (92.3 ± 1.8% and 94.0 ± 2.7%, respectively), followed by plasma and leukocytes (81.8 ± 33.1% and 73.5 ± 3.8%, respectively). It was the highest piRNA in cell-free saliva (40.3 ± 17.0%). It had lower concentrations, yet more than 10%, in saliva, urine and urine-cell free (14.9 ± 1.7%, 15.6 ± 12.9% and 14.5 ± 12.5, respectively). Urine and urine-cell free had piR-019825 as the highest piRNA (46.0 ± 40.4% and 58.7 ± 32.1%, respectively). Interestingly, piR-019825 was the second highest piRNA in plasma where it represented 15.2 ± 30.4% of the piRNA content (Table 9). An additional file contains the list of piRNAs at an average of 1% or more of the entire piRNAs counts of each bodily fluid (Additional file 13: Table S9).

**Table 9:** Predominant piRNAs in each bodily fluid

Body fluid	piRNA	GenBank Accession number	Chromosomal position	Percentage
Blood	hsa_piR_016658	DQ592931	Homo_sapiens:6:80508363:80508389:Plus	(92.3 + 1.8)
Leukocyte	hsa_piR_016658	DQ592931	Homo_sapiens:6:80508363:80508389:Plus	(73.5 + 3.8)
	hsa_piR_000552	DQ570687	Homo_sapiens:22:38045003:38045030:Minus	(5.6 + 0.6)
Plasma	hsa_piR_016658	DQ592931	Homo_sapiens:6:80508363:80508389:Plus	(81.8 + 33.1)
	hsa_piR_019825	DQ597218	Homo_sapiens:1:227740227:227740256:Plus	(15.2 + 30.4)
Serum	hsa_piR_016658	DQ592931	Homo_sapiens:6:80508363:80508389:Plus	(94.0 + 2.7)
Saliva	hsa_piR_014620	DQ590013	Homo_sapiens:5:93930930:93930956:Minus	(16.3 + 14.1)
	hsa_piR_016658	DQ592931	Homo_sapiens:6:80508363:80508389:Plus	(14.9 + 1.7)
	hsa_piR_019521	DQ596805	Homo_sapiens:11:10487516:10487542:Minus	(10.5 + 2.7)
	hsa_piR_000552	DQ570687	Homo_sapiens:22:38045003:38045030:Minus	(7.6 + 2.7)
	hsa_piR_018780	DQ595807	Homo_sapiens:17:72068837:72068864:Plus	(5.8 + 3.3)
	hsa_piR_000805	DQ571003	Homo_sapiens:1:212438966:212438997:Plus	(5.5 + 1.5)
Cell-Free Saliva	hsa_piR_016658	DQ592931	Homo_sapiens:6:80508363:80508389:Plus	(40.3 + 17.0)
	hsa_piR_016659	DQ592932	Homo_sapiens:14:22388242:22388267:Plus	(7.4 + 6.7)
	hsa_piR_019521	DQ596805	Homo_sapiens:11:10487516:10487542:Minus	(5.2 + 2.0)
	hsa_piR_000552	DQ570687	Homo_sapiens:22:38045003:38045030:Minus	(5.1 + 3.3)
	hsa_piR_020450	DQ598104	Homo_sapiens:9:133350930:133350959:Plus	(5.1 + 0.9)
Urine	hsa_piR_019825	DQ597218	Homo_sapiens:1:227740227:227740256:Plus	(46.0 + 40.4)
	hsa_piR_016658	DQ592931	Homo_sapiens:6:80508363:80508389:Plus	(15.6 + 12.9)
	hsa_piR_014620	DQ590013	Homo_sapiens:5:93930930:93930956:Minus	(5.6 + 5.0)
Cell-Free Urine	hsa_piR_019825	DQ597218	Homo_sapiens:1:227740227:227740256:Plus	(58.7 + 32.1)
	hsa_piR_016658	DQ592931	Homo_sapiens:6:80508363:80508389:Plus	(14.5 + 12.5)
	hsa_piR_004153	DQ575660	Homo_sapiens:3:156861576:156861607:Plus	(6.0 + 3.3)

Molecules that represent an average of 5% or more of the entire piRNAs count of each bodily fluid are listed

**Fig. 10:**
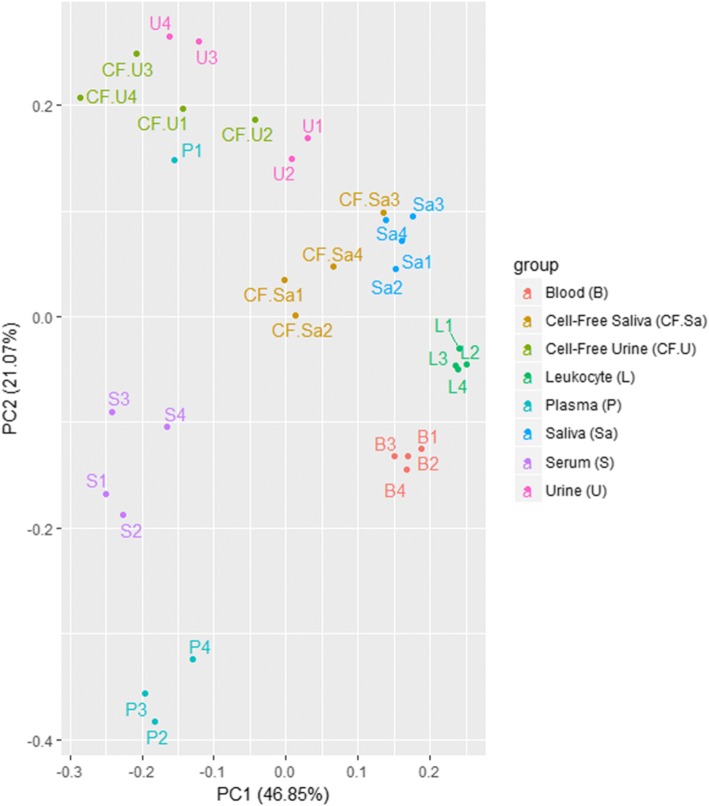
Principal component analysis of piRNAs in each bodily fluid. Analysis was generated based on TMM-normalized piRNA counts. Samples are clustered based on biology and fluids that share similar origin have close clustering. Close clustering is seen between the following fluid pairs: blood/leukocytes, saliva/cell-free saliva and urine/cell-free urine. Serum and plasma show distant clustering from the other fluids

**Fig. 11:**
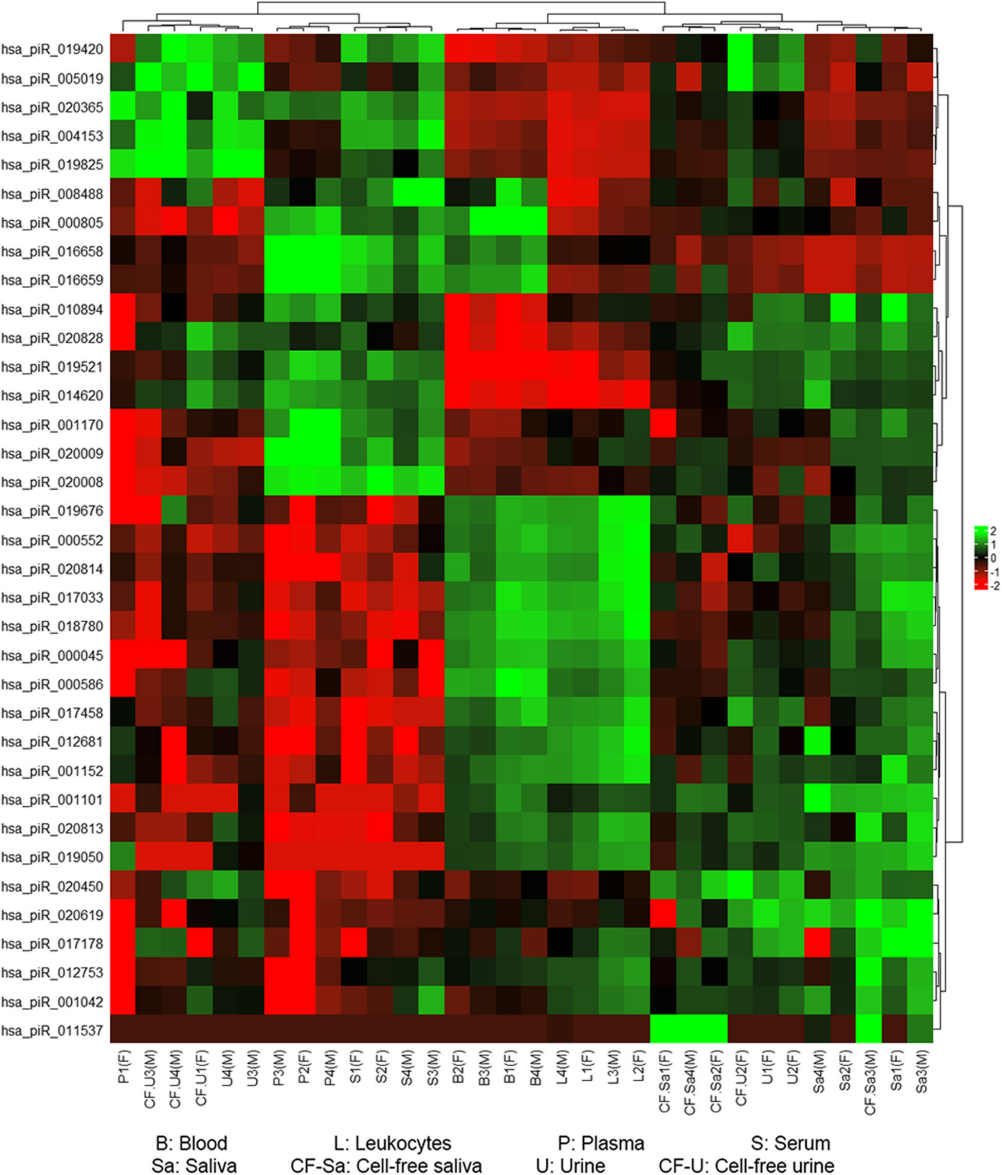
Heatmap clustering of piRNAs in each bodily fluid. The sex of sample donor is indicated as (F) for female donors or (M) for male donors. Heatmap was generated using Z scores of TMM-normalized tRNA counts. The dendrogram shows distant clustering of urine samples (total and cell-free) based on sex. Male urine and cell-free urine samples clustered close to serum and plasma while female urine and most of cell-free urine samples are clustered with saliva

Read counts of piRNAs were TMM-normalized at a CPM corresponding to a minimum of 5 counts in a library. The normalized counts were used to generate a PCA plot (Fig. 10). Close clustering was obtained between blood and leukocytes, saliva and cell-free saliva, and urine and cell-free urine. Plasma and serum showed relatively distant clustering from each other and from the other fluids. A similar clustering pattern was obtained from the heatmap. However, urine samples and most of the cell-free urine samples showed sex-dependent clustering (Fig. 11). Female urine samples clustered close to saliva samples, while male urine samples clustered close to plasma and serum samples. While clustering reflects the close biology of the samples, it has a distinct trend when compared to that of miRNA as it showed more overlap between invasive and non-invasive fluids.

## Discussion

In this study, we investigated small RNA profiles in various bodily fluids using NGS in order to understand the distribution of the various biotypes between fluids as well as the molecular signature of each fluid. Purified total RNA from each fluid showed large variations in the RNA content and integrity. Saliva, cell-free saliva and blood have the highest RNA content. These elevated levels in both blood and saliva are due to their high number of cells. However, the high RNA levels in the cell-free saliva preparation were most likely due to the high bacterial content, as the cell-free preparation steps utilized in this study were aimed at removing mammalian but not bacterial cells. The lowest RNA yields were found in both total and cell-free urine preparations. This indicated very low cell content as well as minimal cell-free RNA content of urine samples collected from healthy individuals. Intra-fluid RNA yields are more consistent from fluids that have high cell content (blood, saliva and leukocytes). RNA integrity as measured by RIN value was also dependent on the cellular content of the fluid. This severely affected the RNA integrity of urine, cell-free urine, plasma and serum where they almost have no measurable integrity. In fact, while many small RNA-Seq library preparation pipelines require RNA with a minimum RIN value, this study showed that many bodily fluids of low cell content may not meet such requirements. However, in this study libraries were successfully constructed from RNA samples with low or no RIN value, suggesting that using RIN value as a sole determination of RNA quality may not be universally applicable.

For better representation of the actual library preparation workflow, we used a standard NGS preparation protocol based on equal input volumes from each bodily fluid preparation. An average of 12.57 ± 3.54 million reads were obtained from all samples, despite the large inter-fluid and intra-fluid variations in RNA yield and integrity. This indicates that regardless of sample type, a clean purification and robust library preparation can yield similar sequencing read outcomes. The critical parameter that would then define suitability of a sample to be used in small RNA profiling and discovery would be its actual biotype content. Read alignment to human genome varied between the different fluids based on their molecular composition. The lower percentage of leukocytes reads successfully aligned to the human genome is a result of their higher rRNA content. The lower percentage of saliva and cell-free saliva reads successfully aligned to the human genome is due to the high percentage of unmapped reads. We conducted exogenous mapping analysis on the saliva and cell-free saliva samples and 85–90% of the unmapped reads were mapped to bacteria (Additional file 14: Figure S3 and Additional file 15: Figure S4). This agrees with results of a recent study by Yeri et al. [70]. This in turn reduces the amount of valuable human RNA molecules that can be used in profiling or discovery. Efficient removal of salivary bacteria can be achieved by centrifugation [71]. However, removal of bacteria in this manner would reduce the number of reads directed towards bacterial sequences, thereby hindering the study of bacterial communities and/or pathogens that might be contained within these fluids.

While the profile of leukocytes showed a fairly even distribution of biotypes, all the other bodily fluids showed predominant reads from one or more biotypes. This can be of a significant value if these predominant biotypes are of known biological importance such as miRNAs, tRNAs or piRNAs. Blood has the highest levels of miRNAs (86.6 ± 12.3), which were about 3-fold the levels of leukocytes miRNAs (29.9 ± 3.3). A large portion of these blood miRNAs are lost from plasma and serum, as the separation and coagulation processes might be of the factors that affect miRNA distribution and recovery. Variations between plasma and serum miRNA content results from the stress during coagulation [18]. Non-invasive fluids had lower miRNA fractions, keeping in mind that they had a large percentage of unmapped reads (about 50% of saliva and 20% of urine reads). The relatively high bacterial content of saliva as well as the filtered and diluted nature of urine were key factors in this result. Recent analysis of urine, saliva and plasma miRNAs from NGS data showed lower miRNA counts from urine and saliva [70]. Profiling of miRNA in bodily fluids by RT-PCR in an earlier study showed similar low urine RNA concentrations and low numbers of detected miRNAs, while saliva had the highest number of miRNAs among the studied fluids [72]. The information obtained in our results could be used to guide methods for targeting specific biotypes in bodily fluids (via enrichment, separation, or depletion, for example).

The various bodily fluids have unique miRNA, tRNA and piRNA profiles that characterize the type and origin of the fluid as seen from the PCA plots and heatmaps. The different samples are well clustered based on miRNAs according to sample type and their biology. In addition, invasive and non-invasive fluids have distinct profiles and less variations between the fluids within the same group. PCA plots and heatmaps generated for tRNAs and piRNAs show a biology-related clustering, with overlap between invasive and non-invasive fluids. An interesting observation was the differential clustering of tRNAs and piRNAs from urine and cell-free urine samples based on the sex of the donor, indicating sex-related expression of these molecules. In addition, urine showed close clustering to serum, indicating that the latter might be the true liquid part of blood. The differentiation of the three biotypes (miRNA, tRNA and piRNA) in the different bodily fluids might be a result of fluid origin and biological functions. The higher impact of origin of fluid on miRNA distribution may refer to more specialized functions of miRNA in comparison to piRNAs and tRNAs, which might be involved in more general biological roles.

Non-invasive fluids had almost half the number of identified miRNAs. For urine, this may result from the filtering process by the kidneys. For saliva, the lower circulating nucleic acid content, relative to blood-related fluids may be the cause [73–76]. The larger miRNA fraction and lower numbers of unmapped reads for the invasive fluids explained their higher number of identified miRNAs. Almost every fluid had unique miRNAs that are specific only to that fluid, providing a specific signature for each fluid. The number of common and unique miRNAs between two fluids varied depending on biological relatedness. The invasive fluids collected in this study were more similar to the other invasive fluids, and the non-invasive fluids were more similar to the other non-invasive fluids collected. This may be due to the fact that the invasive fluids collected were all blood derived. The fluid-specific unique miRNAs can result from different cells secreting the different fluids. They may also result from the natural filtration process of some fluids, where some molecules are enriched, while others are depleted. None of the unique miRNAs were found among the top 20 most abundant molecules of each fluid. This indicated the need for higher read depth to detect miRNAs that might have specific functions. Five miRNAs: hsa-let-7a-5p, hsa-let-7f-5p, hsa-miR-191-5p, hsa-miR-26a-5p and hsa-miR-486-5p were common among the top 20 most abundant molecules in all fluids, indicating shared origin or function. These five abundant common miRNAs represented a large portion of the miRNA counts of invasive fluids (more than 40%), while they were relatively lower in non-invasive fluids (less than 30%). There was a set of 139 core miRNAs that are common among the different fluids and a set of 144 miRNAs that were shared between non-invasive fluids and blood. While the levels of these molecules may vary between fluid types, they might be promising biomarker candidates that can be detected from multiple sources, including non-invasive fluids.

An interesting observation was the variation between the most expressed miRNAs in the different fluids. In blood, plasma and serum, hsa-miR-486-5p was the most expressed, while hsa-miR-143-3p was the most expressed in saliva and cell-free saliva and hsa-miR-10b-5p was the predominant miRNA in urine and cell-free urine. We searched these 3 miRNAs on the human miRNA tissue atlas [77] to identify the tissue of origin. High quantile normalized expression levels of hsa-miR-486-5p were found in vein and muscle specimens, while hsa-miR-143-3p was highly elevated in esophagus and relatively high in colon, bladder and prostate specimens. The expression levels of hsa-miR-10b-5p were very high in the epididymis and elevated in kidney, colon and muscle specimens which explains the relatively higher expression levels of this miRNA in male urine samples. Recent studies indicate the importance of hsa-miR-486-5P as a cancer biomarker in non-small cell lung cancer [78], gastric cancer [79] and oral tongue squamous cell carcinoma [80]. It may act as a tumor suppressor miRNA [81] and may also be used to predict the efficacy of cancer vaccine treatment for colorectal cancer [82]. However, in many other cancer studies, this miRNA was not deregulated.

The large breadth of unique miRNAs found in blood, combined with an abundance of predicted novel miRNA candidates demonstrates the superiority of blood for miRNA profiling and discovery. However, blood has high levels of hsa-miR-486-5p, representing over 50% of its miRNA content. Other bodily fluids that had a relatively high miRNA content are plasma and, to some extent, serum. Plasma did not suffer from the presence of a predominant molecule as did blood and serum. This resulted in a high number of predicted novel miRNA candidates compared to all the other bodily fluids, making plasma a good alternative to blood. However, depletion of hsa-miR-486-5p from blood and serum could be a useful tool to direct a greater proportion of reads to other miRNA sequences.

Both saliva and urine did not offer the same advantage as the invasive fluids. They had lower miRNA content and this affected their molecular diversity. The most expressed miRNA in saliva and cell-free saliva was hsa-miR-143-3p (10–15%). It is also the second most expressed miRNA in saliva exosomes [83]. It is differentially expressed in senescence [84] and as a tumor suppressor in gliomas [85]. MicroRNA hsa-miR-10b-5p represented about 38–45% of urine and cell-free urine miRNAs. It has been recently reported to be the most expressed miRNA in urine samples [86]. hsa-miR-10b-5p plays a role in carcinoma metastasis and is overexpressed in colorectal cancer [87–90]. Due to its high expression, a lower proportion of reads will map to other miRNA sequences and its depletion should be considered as a priority for improving the diversity of miRNAs within urine specimens.

Novel miRNA prediction was not as efficient when dealing with non-invasive fluids. Their low signal-to-noise ratio made it hard to obtain accurate prediction. The only exception was the cell-free saliva, where a fair signal-to-noise ratio was achieved, and 7 novel miRNA candidates had been identified. It also had double the number of unique miRNAs compared to the other saliva and urine samples. Due to the removal of mammalian cells by centrifugation, cell-free saliva usually captures more circulating miRNAs than total saliva or the cellular fraction of saliva [20, 91]. This made the cell free saliva sample superior in terms of discovering unique and novel miRNAs candidates compared to the other non-invasive fluids. Matches were found between the miRCarta database of newly predicted human miRNAs and the predicted novel miRNA candidates of the invasive fluids (30 to 66%), while no matches were found between miRCarta and the non-invasive fluids. This might be due to the large number of miRNA studies from the invasive fluids as well as the higher counts and diversity of miRNAs from these fluids compared to the non-invasive fluids. This further indicates the higher potential of the invasive fluids in novel miRNA prediction. Although our findings are based on prediction of candidate miRNA and have not been validated by another technique, they showed the potential of these various fluids in novel miRNA discovery.

While tRNA fragments are a minor portion of blood and plasma small RNAs, they were well represented in serum and saliva preparations (39–46%) and were the major small RNA species of urine and cell-free urine (> 90%). The main component of these tRNAs in all the fluids was tRNA^Gly^ (72.0 to 87.6%), followed by tRNA^Glu^ (6.7 to 21.4%). Urine samples, unlike the other fluids, had high sample-to-sample variations, with tRNAs ranging from 47 to 98% of small RNAs. Similar variations have been reported in a recent study on urine from ovarian cancer patients [86]. However, these variations may be correlated to the sex of the individual, where male urine has over 90% and female urine has about 70% or less. A larger study is needed to validate these findings. It is also interesting that the specific tRNA molecular composition of these tRNA fractions is consistent. Despite the overall fluctuations in urine tRNA fractions, changes in the molecular signature of tRNA molecules might still be valid for potential biomarker discovery. However, it may be limited by the lower urine tRNA molecular diversity, compared to blood, plasma and saliva. These variations in diversity were also observed between plasma, saliva and urine in a recent study [70]. However, the percentage abundance of molecules was different.

Plasma and leukocytes contain relatively high amounts of piRNA (8 and 5.8%, respectively). All the other bodily fluids contain less than 2%, which is related to the small fraction of piRNAs that are consistently being expressed in normal and cancer cells [92]. It is interesting that a single piRNA molecule, hsa-piR-016658, was the most expressed in all bodily fluids except in saliva, urine and cell-free urine, where it was the second most abundant. This molecule is associated with patients with prostate cancer [93]. The most abundant piRNA molecule in urine and cell-free urine was hsa-piR-019825, which is deregulated in colorectal cancer patients [93]. Given the high number of human piRNAs, they may play a role as an important small RNA species with functional targets that are yet to be elucidated and correlated with various disease conditions. Recent studies have identified differentially expressed piRNA molecules as potential biomarkers of various cancers [94–97]. The relatively high levels of these molecules in plasma might prove important as potential biomarkers. The low piRNAs levels in the other fluids can be overcome by size selection methods, albeit not easily as they overlap with other small RNA species.

Plasma and serum had a large fraction of reads mapping to miscellaneous RNA (misc_RNA) (58 and 35%, respectively). Only 4 YRNA-derived small RNAs (s-RNYs) sequences were elevated within the misc_RNA fractions of these two fluids: RNY4, RNY4P10, RNY4P7 and YRNA.295. It has been previously reported that s-RNYs are abundant in human serum and plasma [98]. They are potential cancer biomarkers and regulators of inflammation and cell death [99, 100].

## Conclusions

Our study showed that it is possible to successfully accomplish NGS of the different bodily fluids (blood, plasma, serum, saliva, cell-free saliva, urine and cell-free urine), even with the high variations in the volumes used for RNA purification as well as the high variations in concentrations of the isolated RNA. Despite the ease of collection and handling of non-invasive fluids, they did not provide the same small RNA diversity and sample consistency as invasive fluids. However, this study showed that these samples can still be routinely profiled. Furthermore, the signatures of these non-invasive fluids are very likely linked to their origin. For example, urine may be a good candidate for studying diseases related to organs such as kidney and bladder, although careful result interpretation should be considered when investigating male and female urine, as their biotypes may be sex-dependent. This observation is limited by the sample size of our study and is yet to be investigated on a large sample size study. An organ and fluid small RNA index might be needed to track and correlate origins and functions of the various molecules. Processing of larger volumes of urine, and bacterial removal from saliva preparations might improve their NGS mapping to human targets. In addition, depletion of specific molecules or selection/enrichment of target molecules from almost every bodily fluid may significantly increase flow cell capacity for target molecules and in turn provide a meaningful read depth. Successful clustering of bodily fluids based on their miRNA distribution can be expanded to cohorts that can be differentiated according to their miRNA, and possibly in combination with other small RNAs. Therefore, a biomarker within these fluids would be the overall biotype distribution and the molecular signature within these biotypes, rather than a single molecule.

## Additional files

Additional file 1: **Figure S2**. Relative biotype distribution among the various bodily fluids of each donor. (TIF 566 kb)

Additional file 2: **Table S1**. Common miRNAs between invasive fluids. (DOCX 14 kb)

Additional file 3: **Table S2**. Common miRNAs between non-invasive fluids. (DOCX 13 kb)

Additional file 4: **Table S3**. Common miRNAs between non-invasive fluids and blood. (DOCX 13 kb)

Additional file 5: **Figure S1**. Venn diagram showing the overlap between blood and the non-invasive bodily fluids. (TIF 536 kb)

Additional file 6: **Table S4**. Common miRNAs between all fluids. (DOCX 13 kb)

Additional file 7: **Table S5**. Unique miRNAs detected in each bodily fluid. (DOCX 14 kb)

Additional file 8: **Table S6**. Unique miRNAs detected in the invasive bodily fluids. (DOCX 14 kb)

Additional file 9: **Table S7**. Unique miRNAs detected in the non-invasive bodily fluids. (DOCX 13 kb)

Additional file 10: **Table S8**. Candidate novel miRNAs detected by miRDeep2 in the different bodily fluids and their matching result to the miRCarta database. (XLSX 43 kb)

Additional file 11: Top 50 tDRs in each fluid with their quantification and coverage percentage. (XLSX 4259 kb)

Additional file 12: Profiles of the top 50 mature tDR in each fluid. (PDF 4737 kb)

Additional file 13: **Table S9**. piRNAs that represent an average of 1% or more of the entire piRNA counts of each bodily fluid. (DOCX 18 kb)

Additional file 14: **Figure S3**. Exogenous mapping of unmapped saliva reads. (PDF 24 kb)

Additional file 15: **Figure S4**. Exogenous mapping of unmapped cell-free saliva reads. (PDF 24 kb)

## Abbreviations

CPM: Counts per million
lincRNA: Long intergenic noncoding RNA
miRNA: MicroRNA
misc_RNA: Miscellaneous RNA
mRNA: Messenger RNA
Mt_rRNA: Mitochondrial rRNA
Mt_tRNA: Mitochondrial tRNA
ncRNA: Non-coding RNA
NGS: Next generation sequencing
PCA: Principal component analysis
piRNA: Piwi-interacting RNA
RIN: RNA integrity number
rRNA: Ribosomal RNA
snoRNA: Small nucleolar RNA
snRNA: Small nuclear RNA
s-RNYs: Y RNA-derived small RNAs
TMM: Trimmed mean of M-values
