# Supplementary figures and images for: Diversity and signature of small RNA in different bodily fluids using next generation sequencing
Source: BMC Genomics. 2018 May 29;19:408. doi: 10.1186/s12864-018-4785-8 (PMC5975555; doi:10.1186/s12864-018-4785-8)

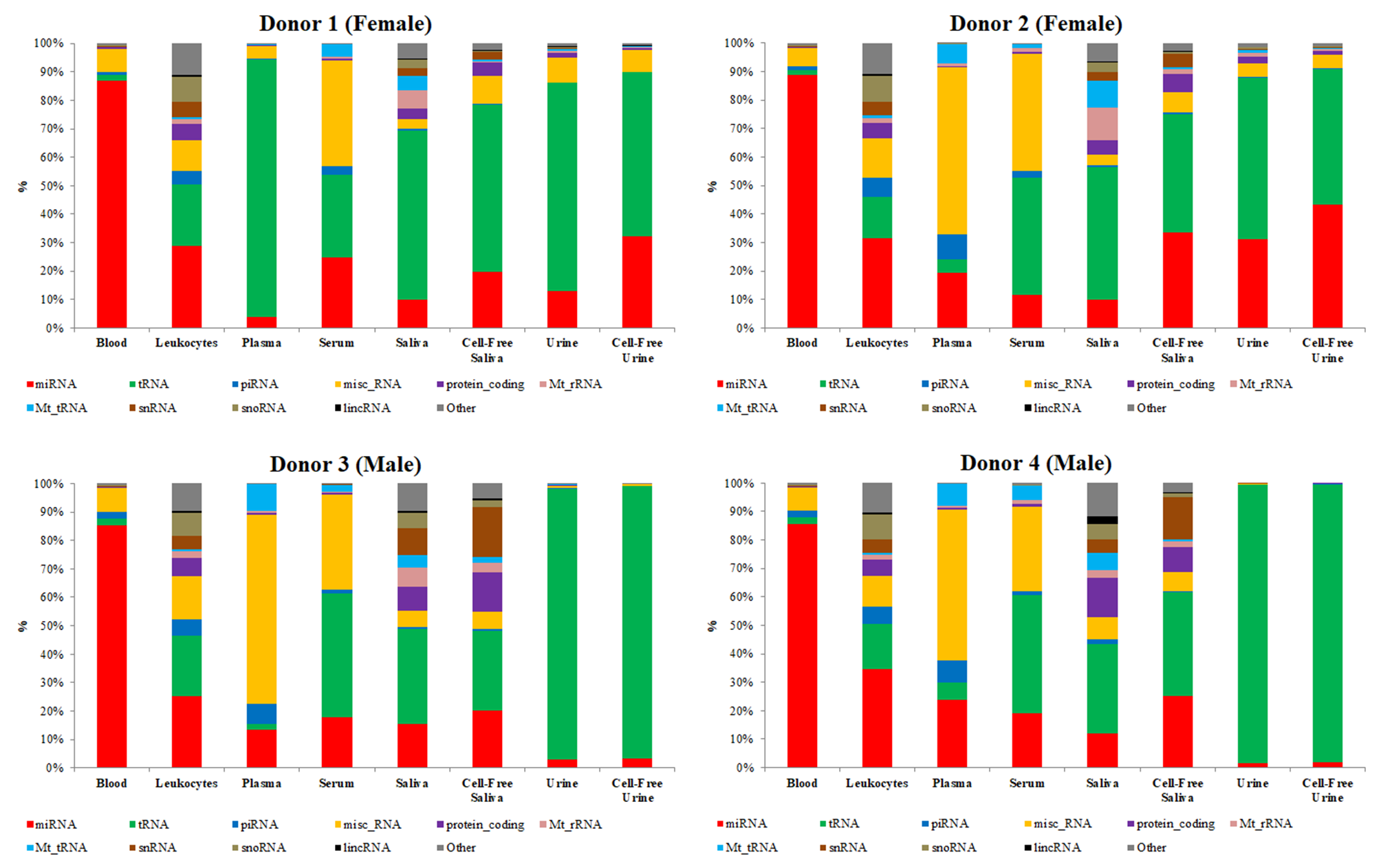

Supplement: Supplementary file 1 — Figure S2. Relative biotype distribution among the various bodily fluids of each donor. (TIF 566 kb) [file 12864_2018_4785_MOESM1_ESM.tif]

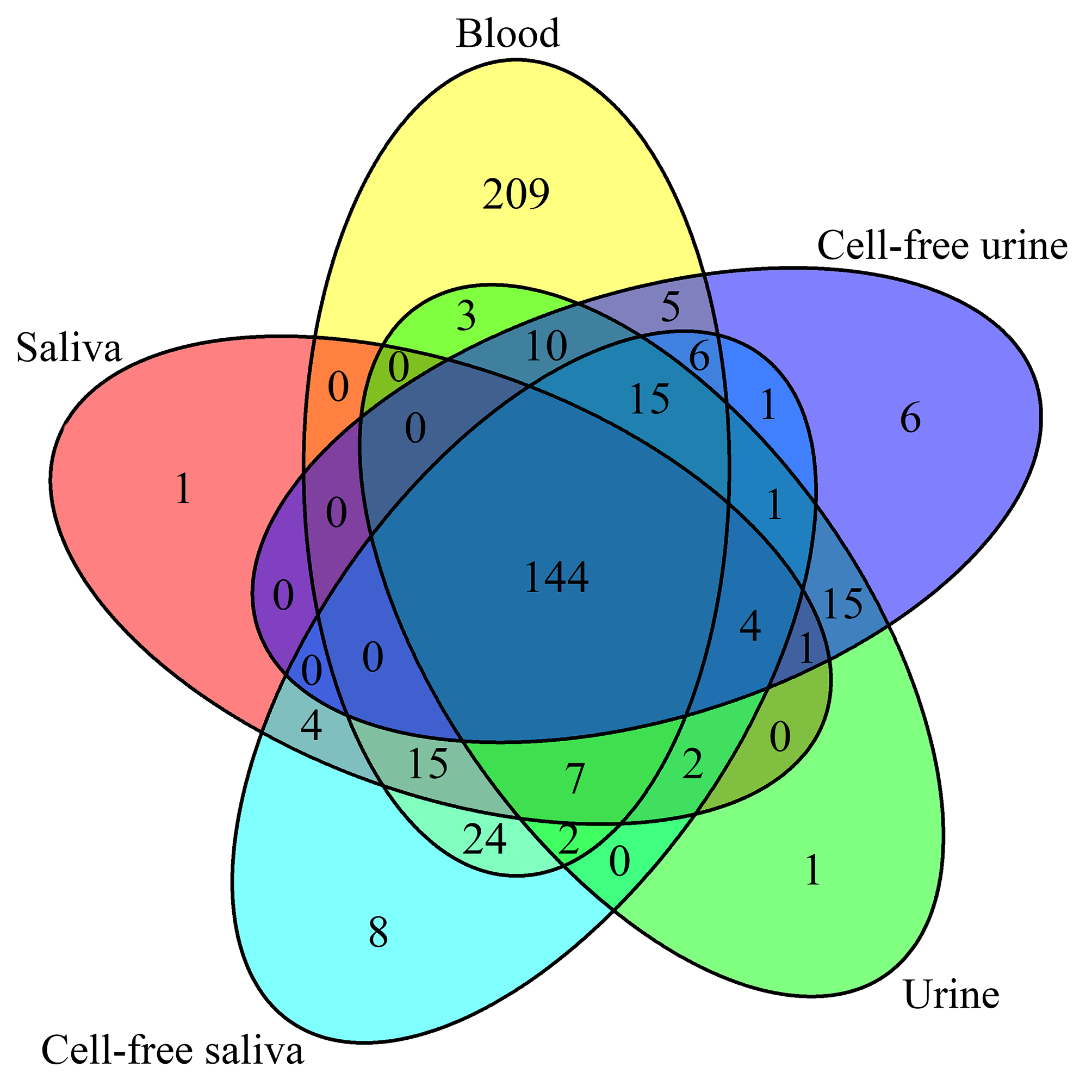

Supplement: Supplementary file 5 — Figure S1. Venn diagram showing the overlap between blood and the non-invasive bodily fluids. (TIF 536 kb) [file 12864_2018_4785_MOESM5_ESM.tif]

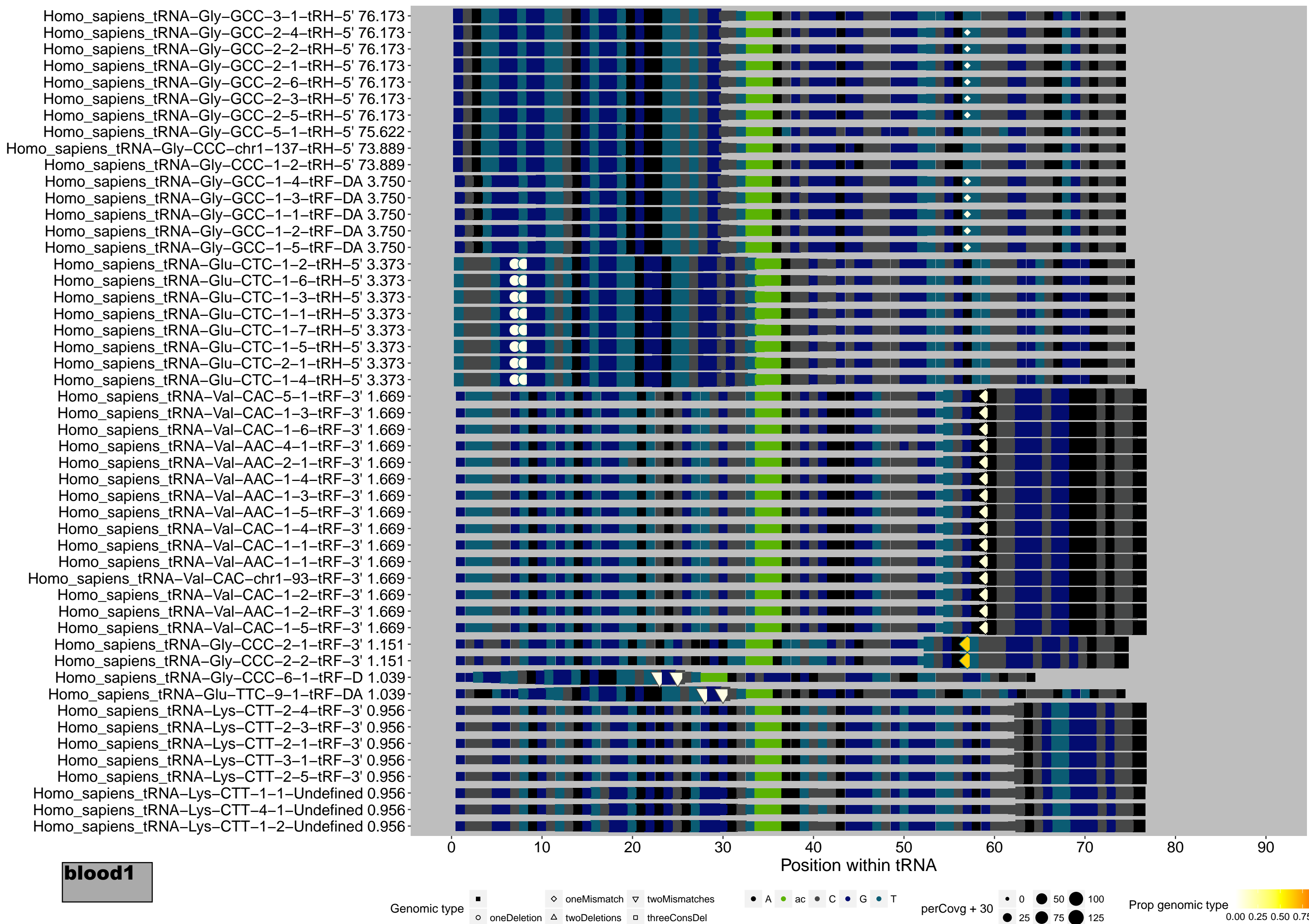

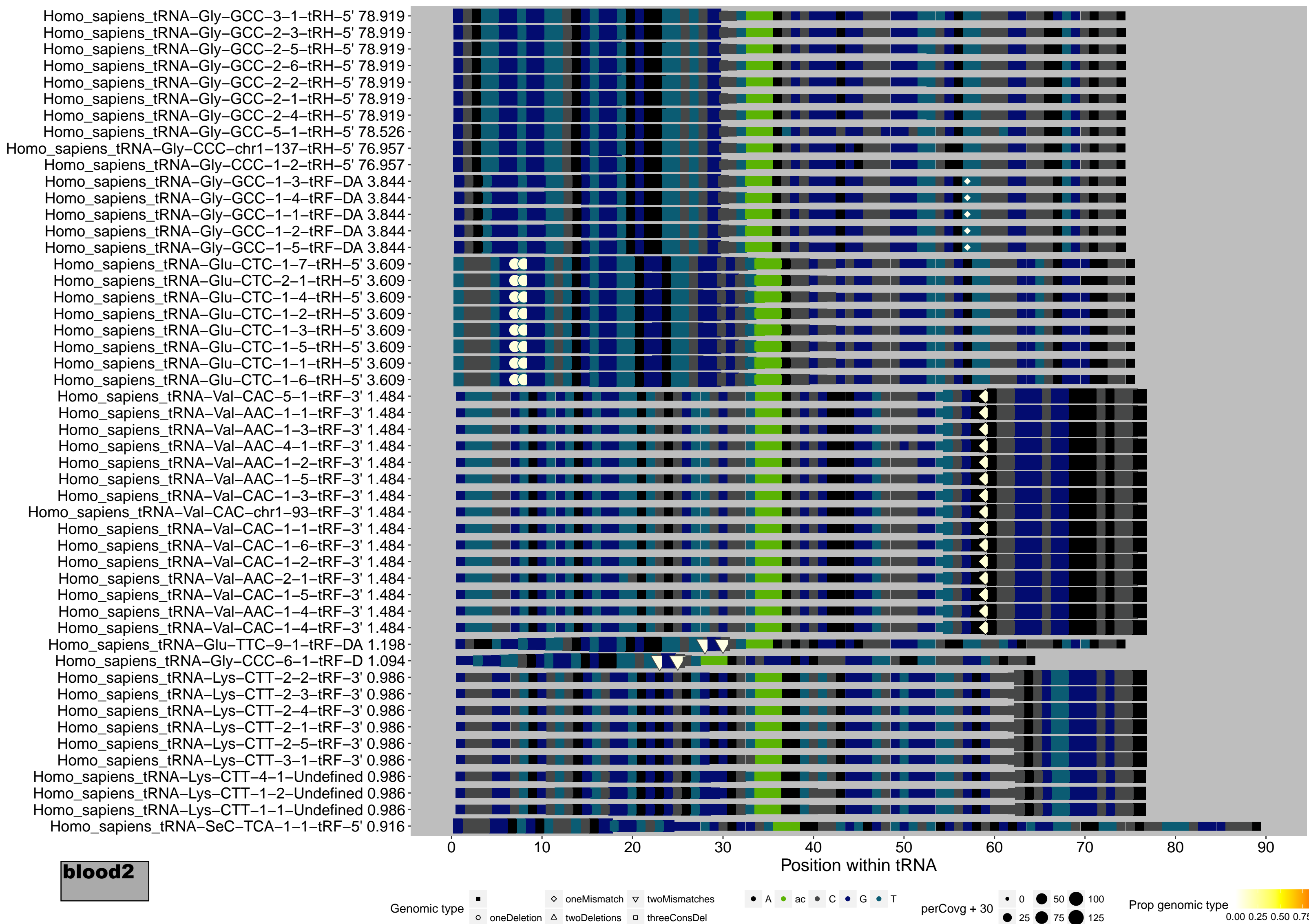

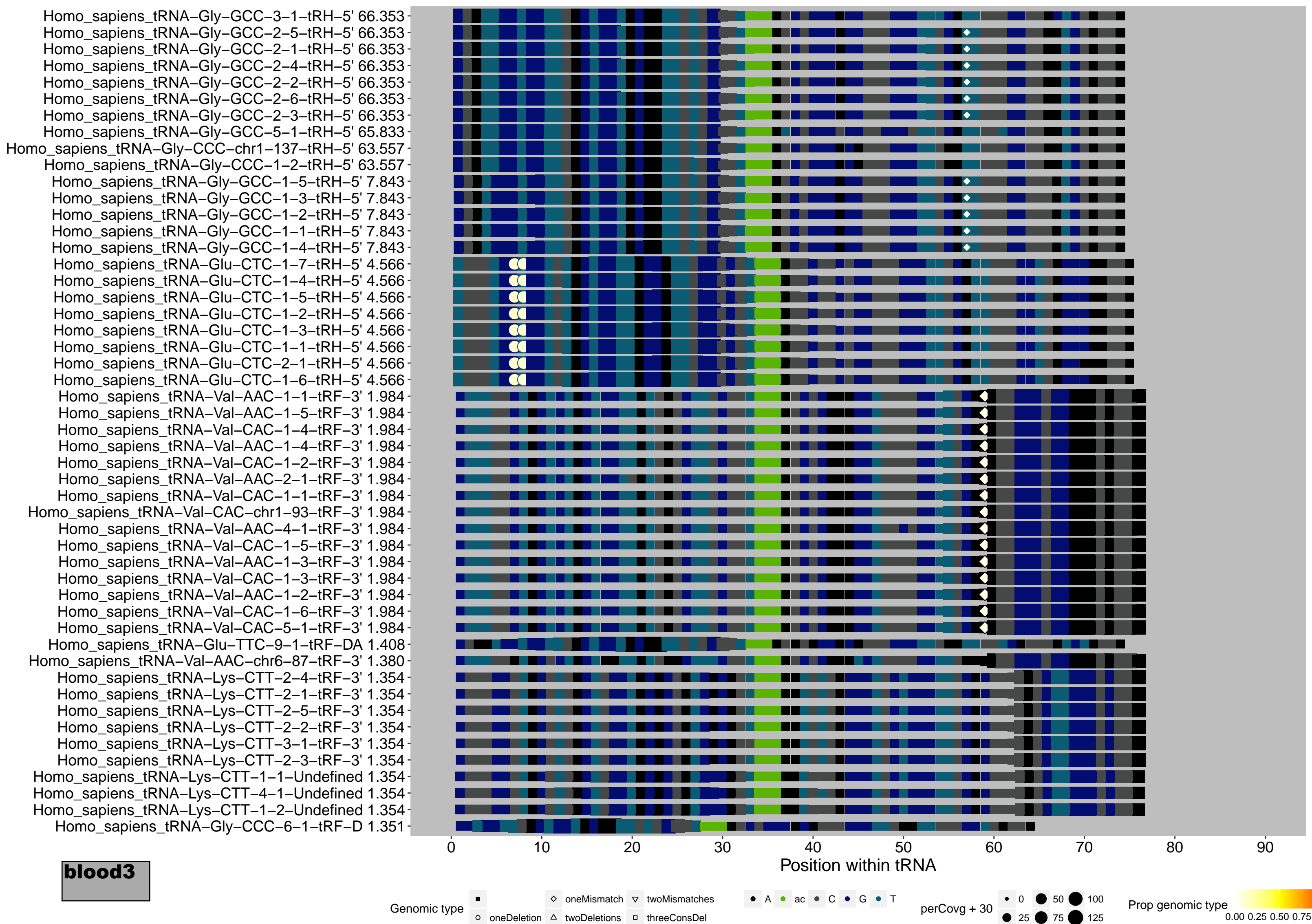

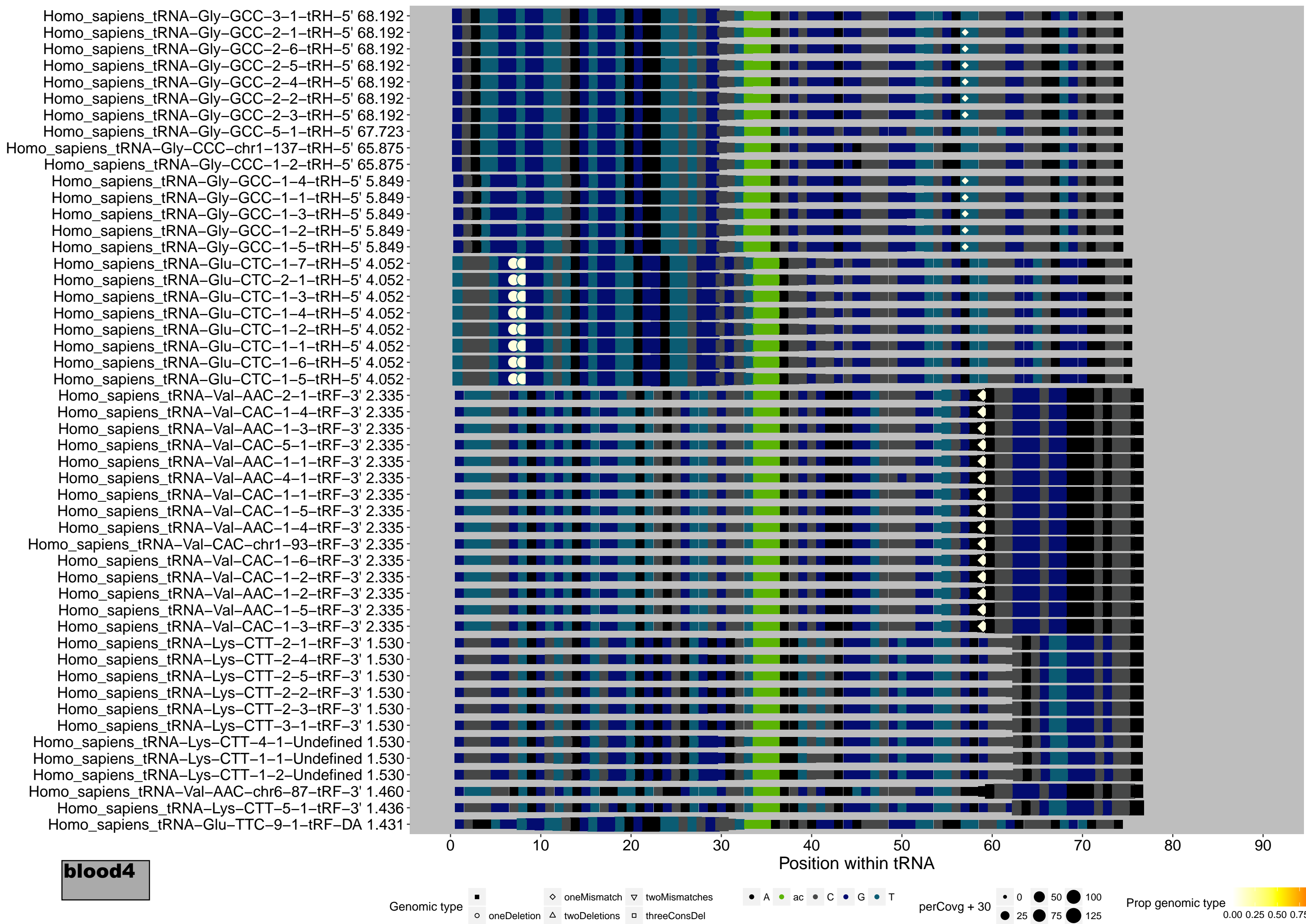

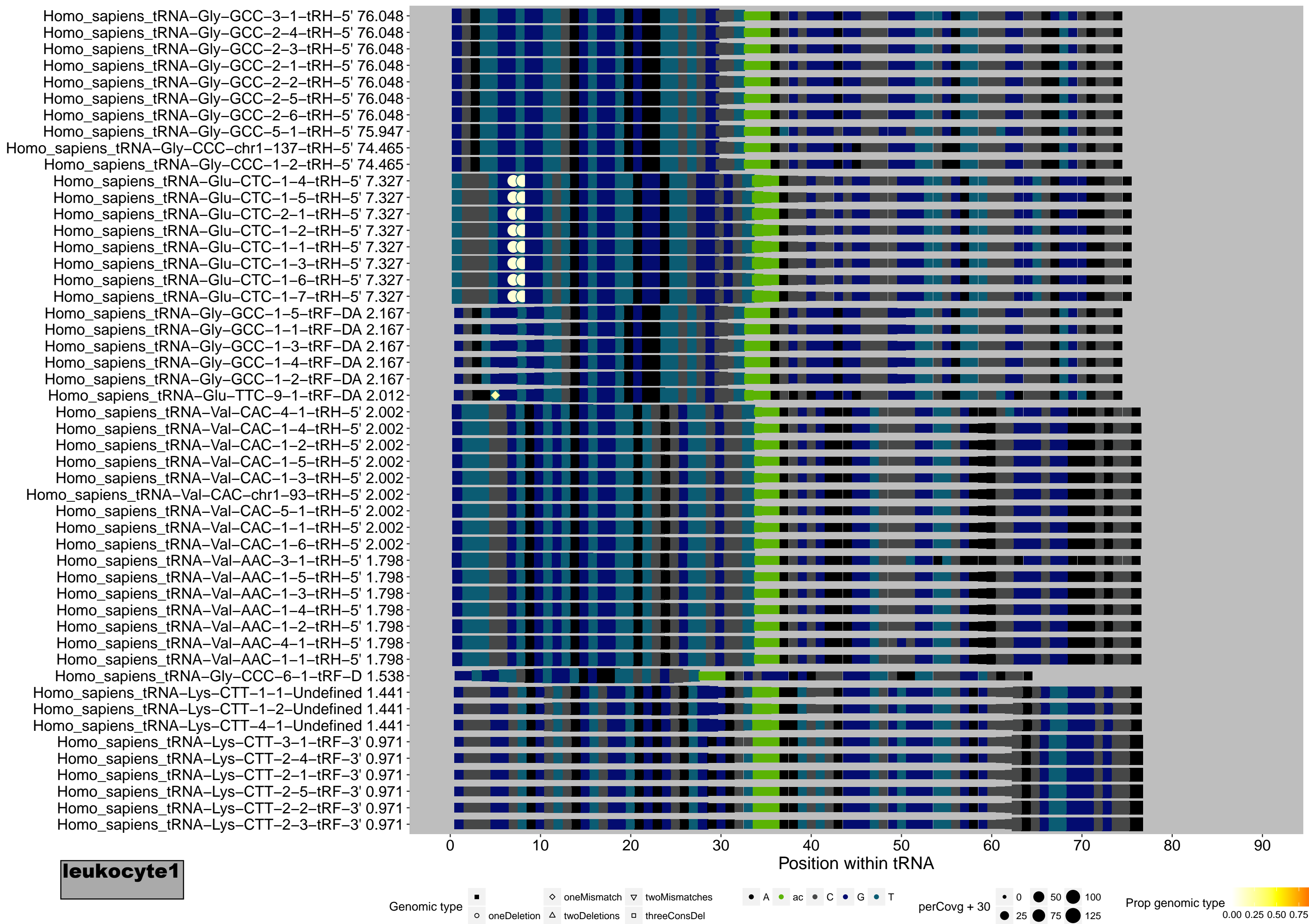

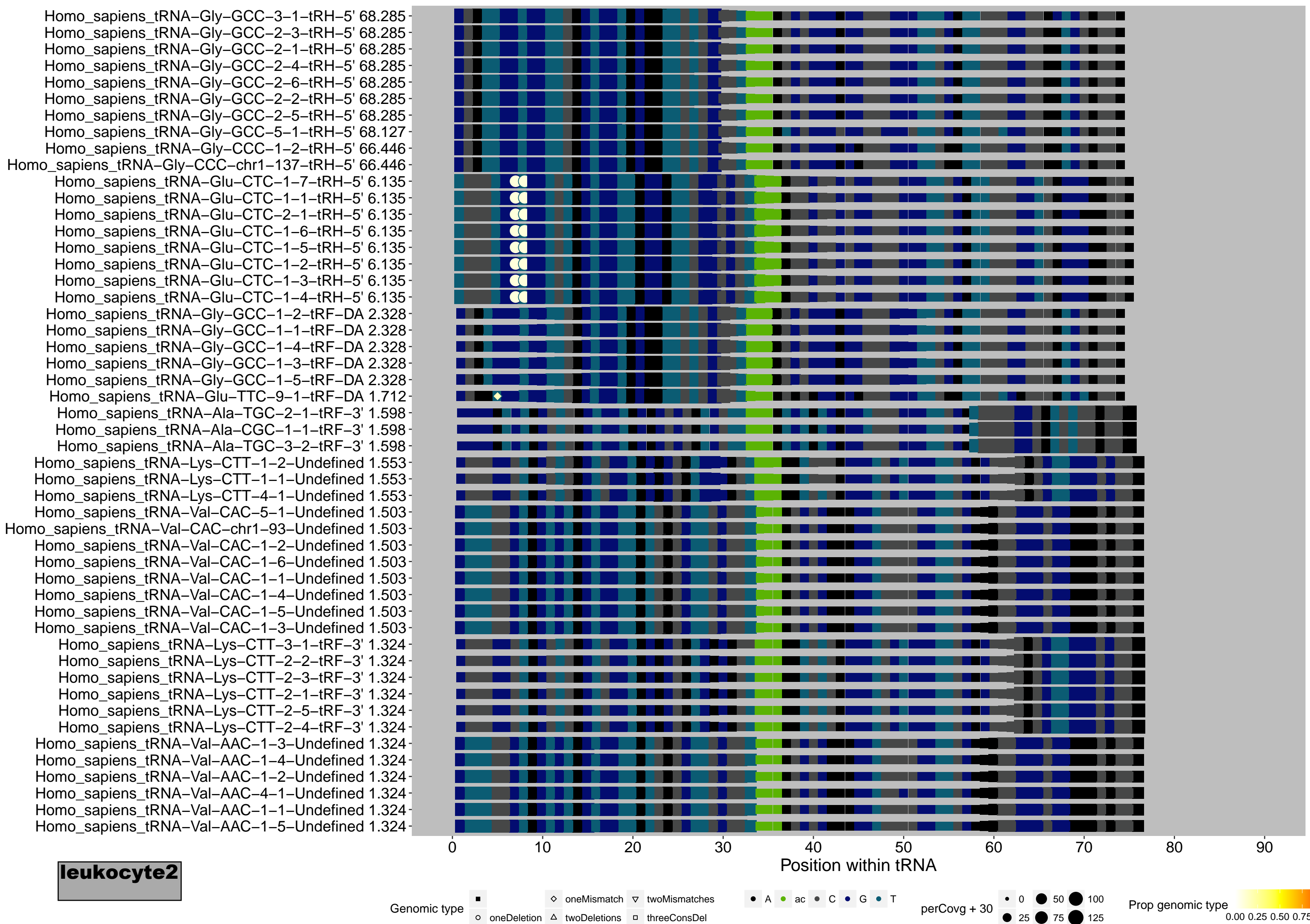

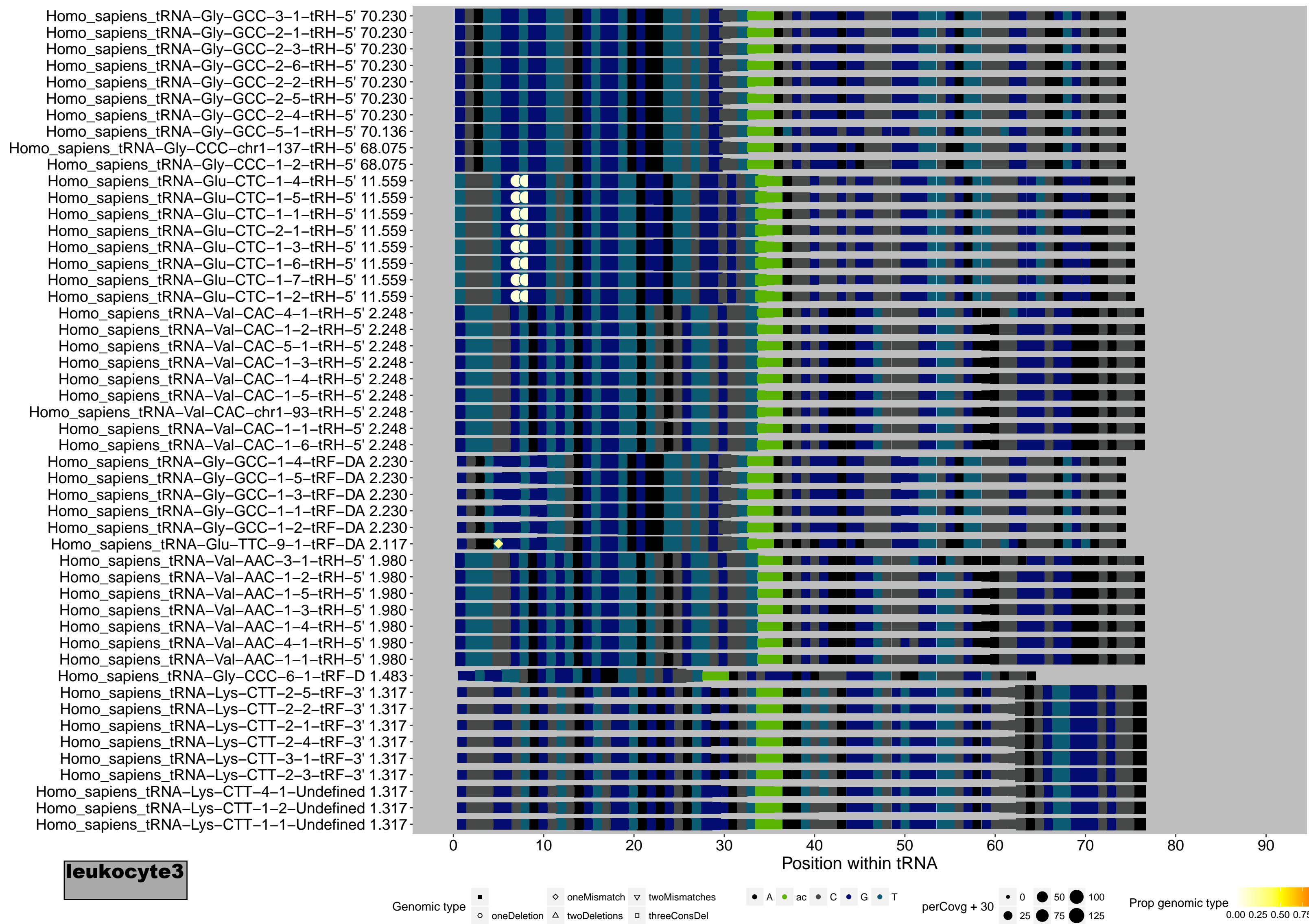

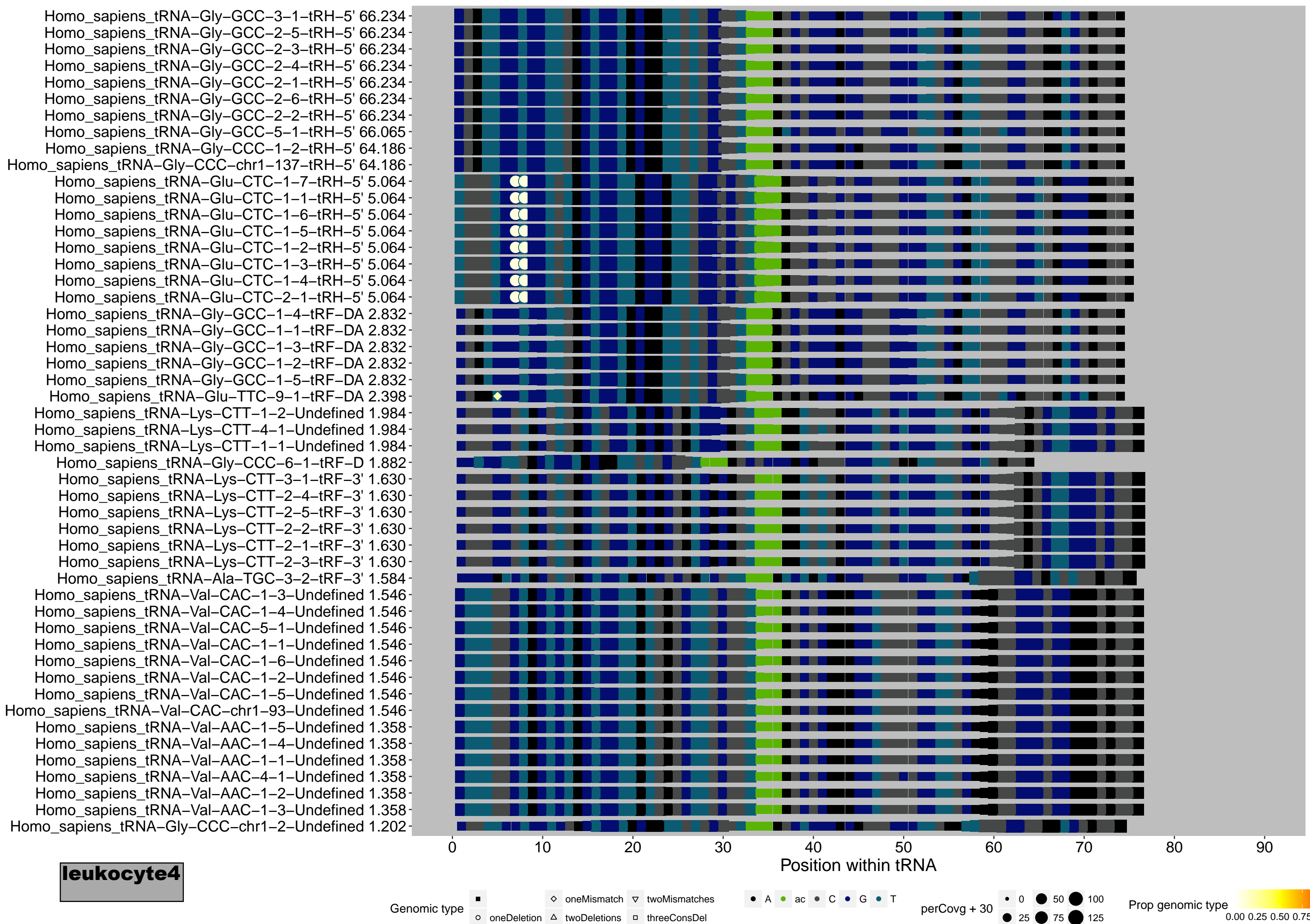

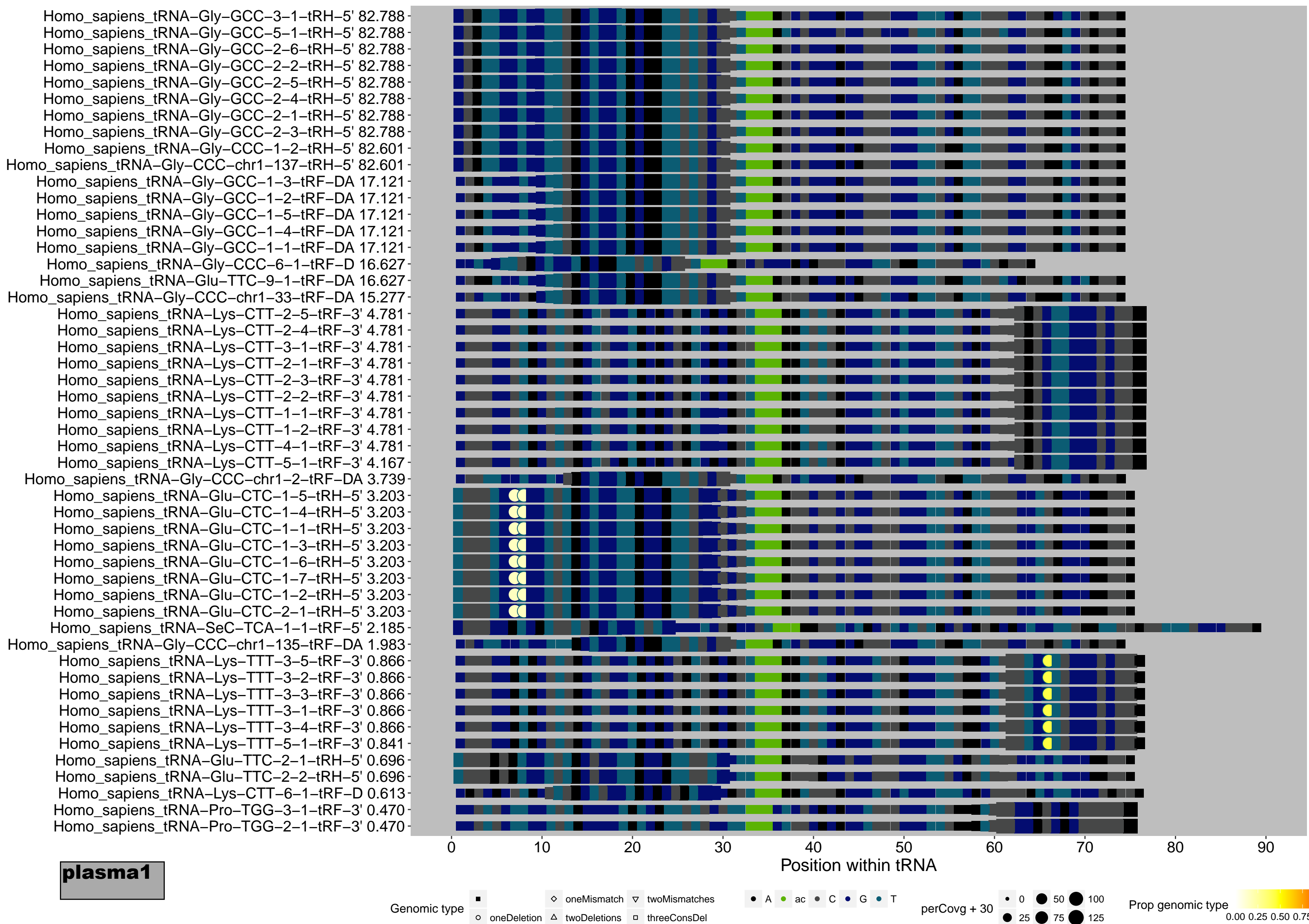

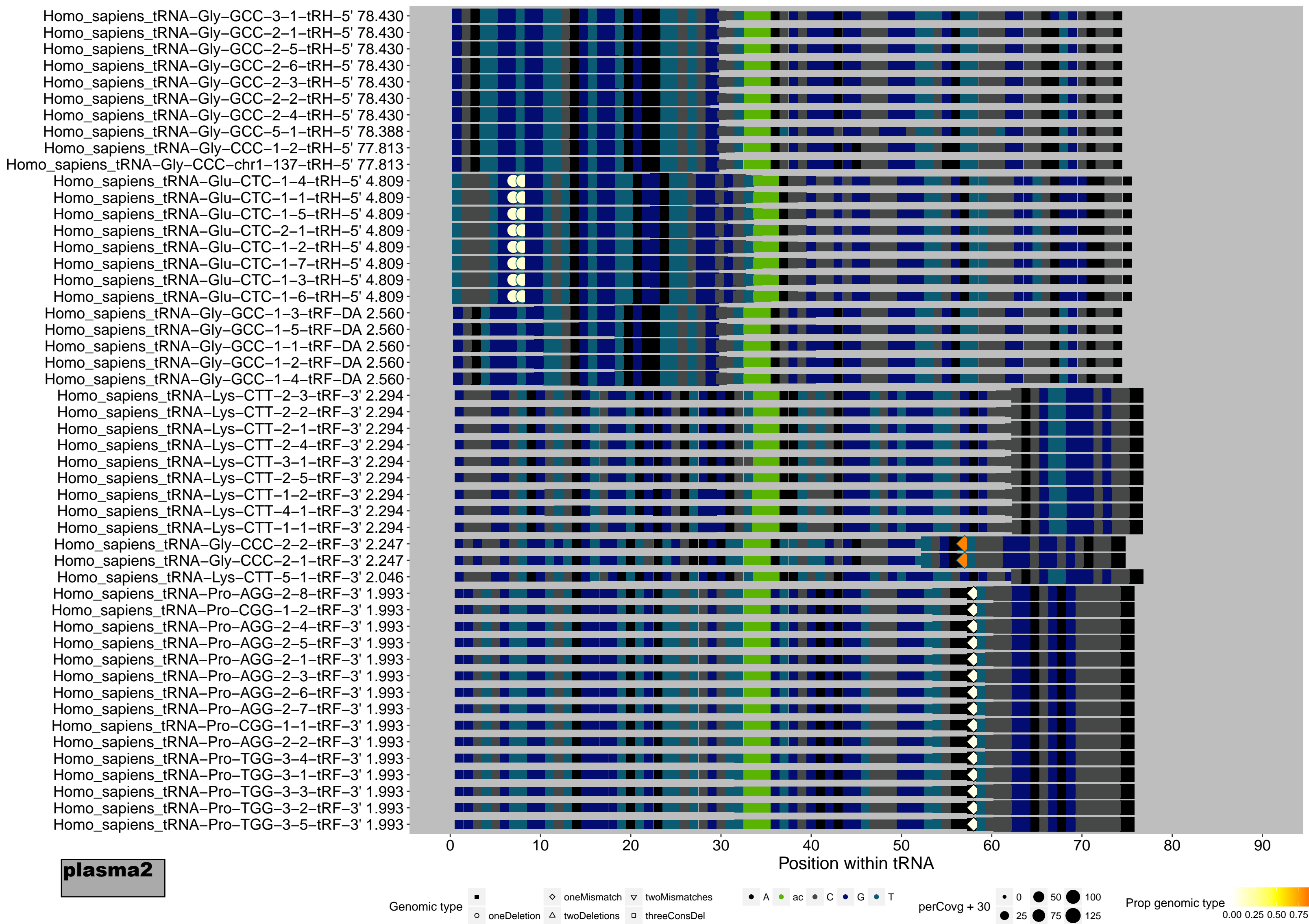

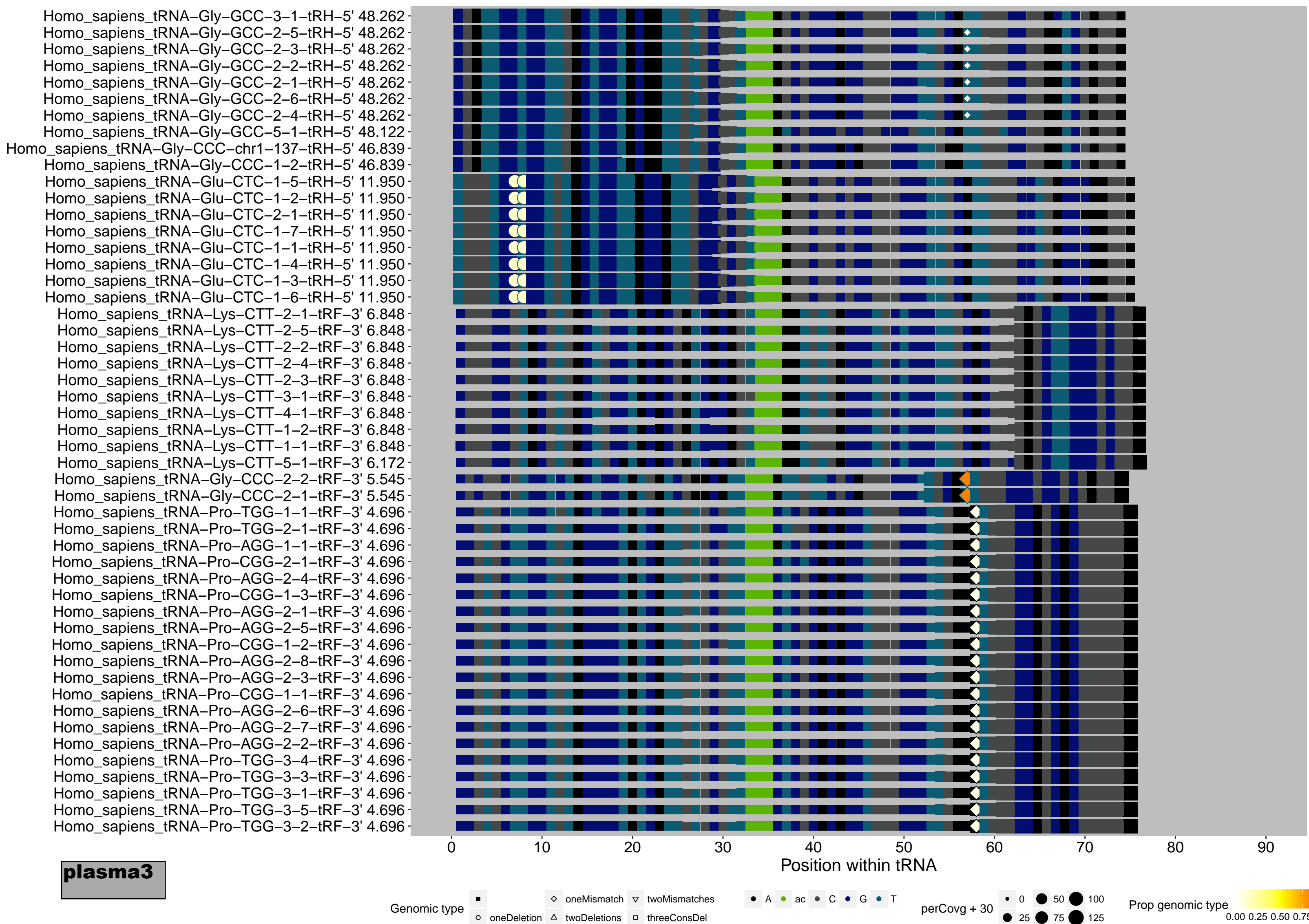

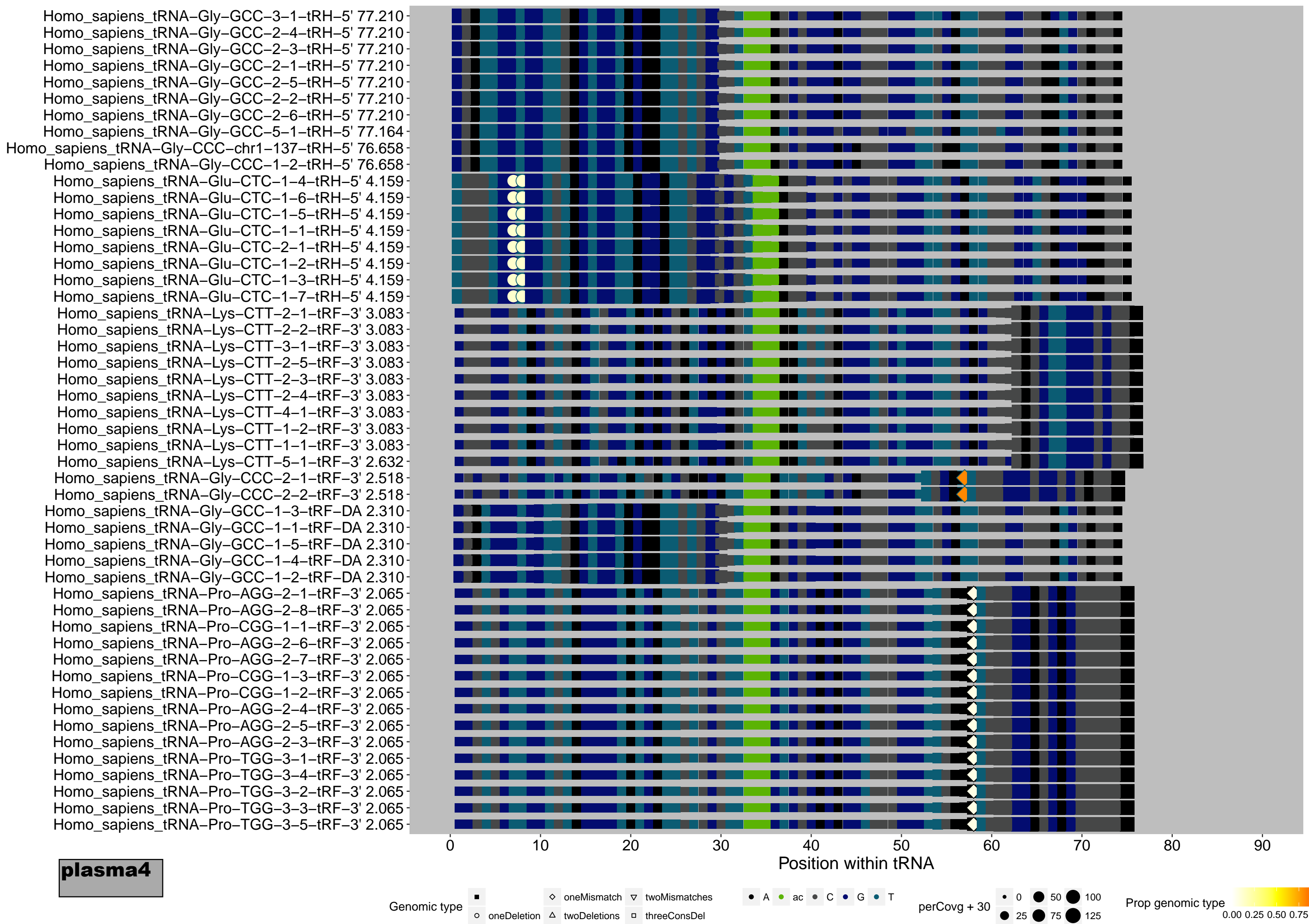

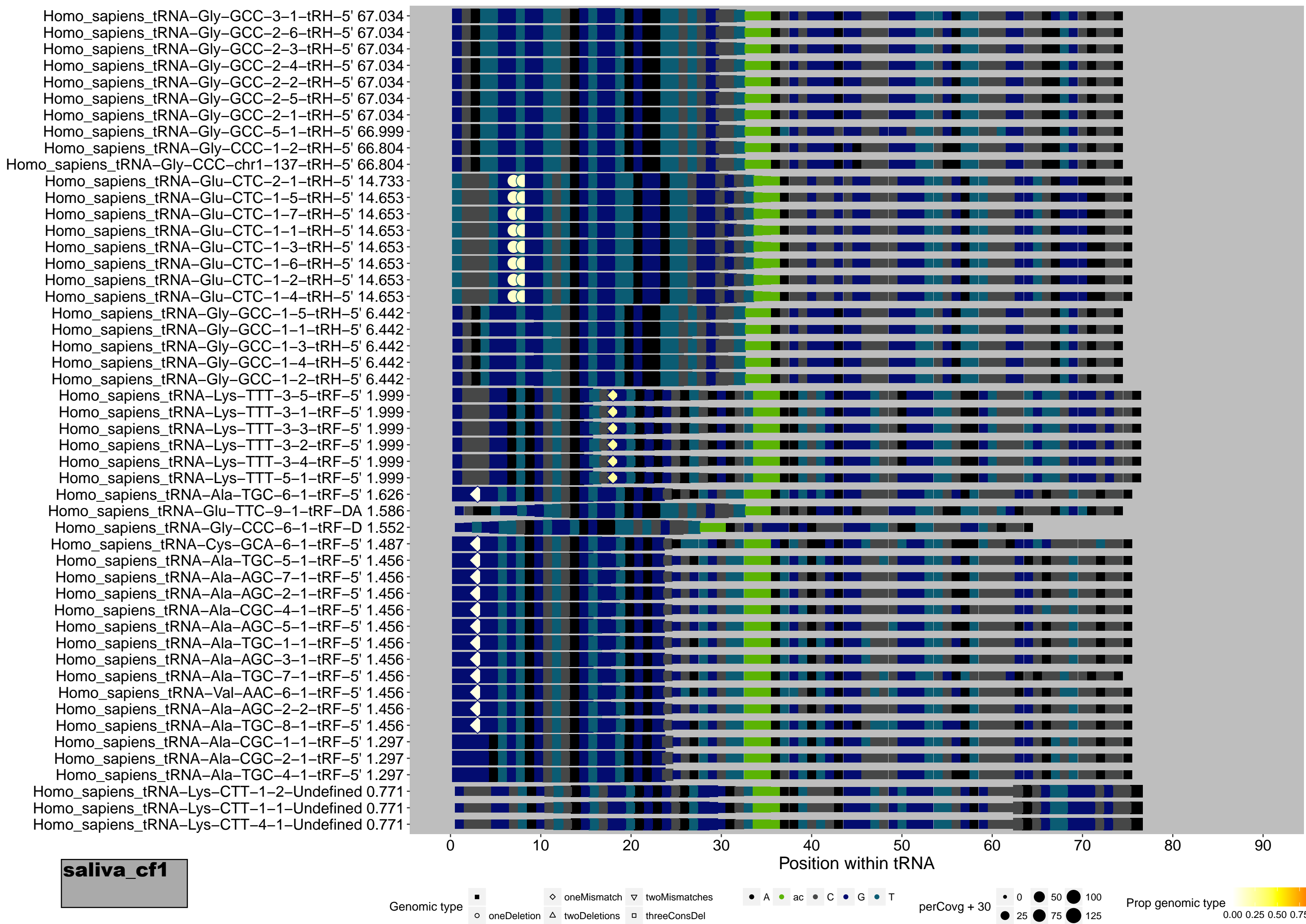

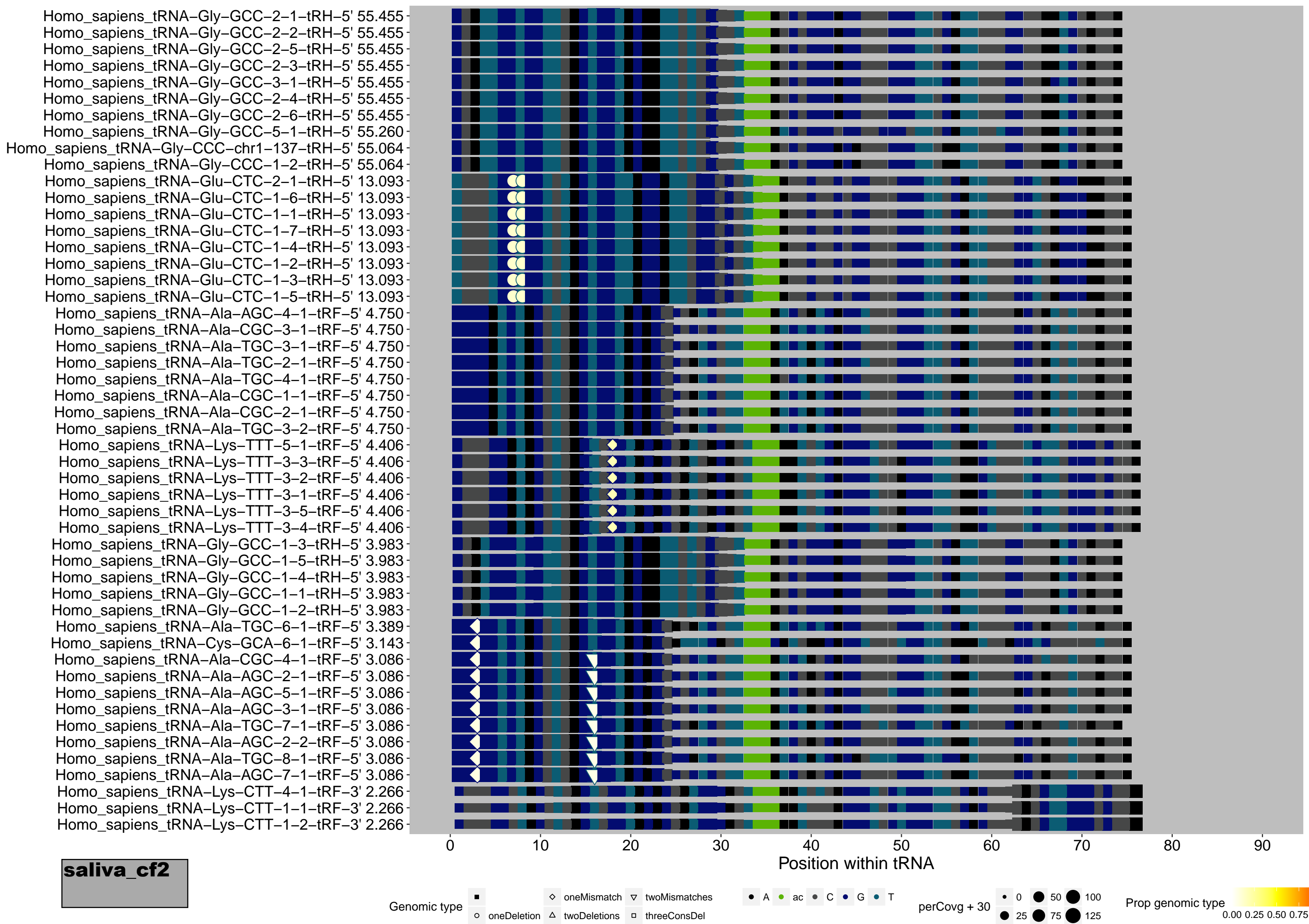

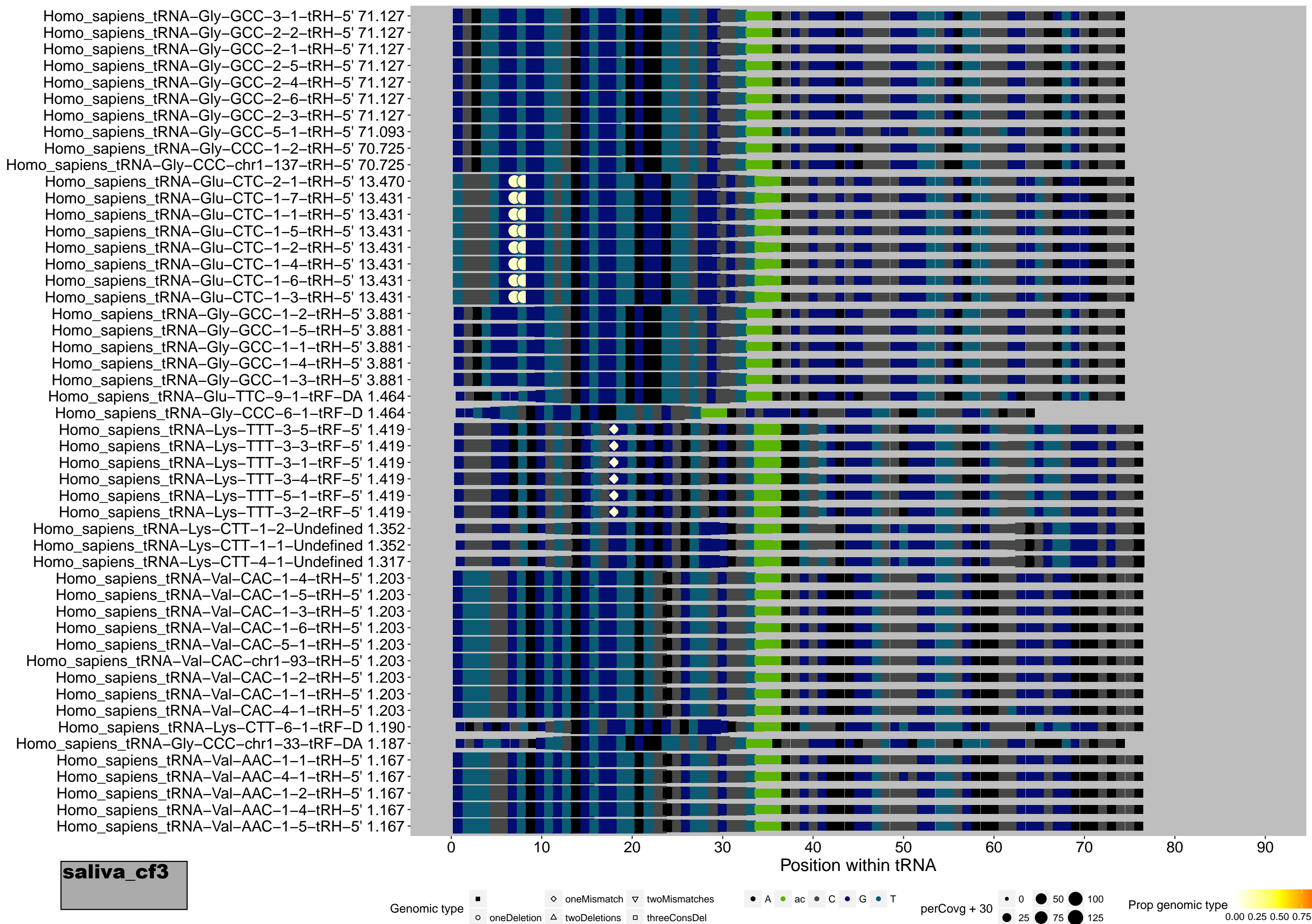

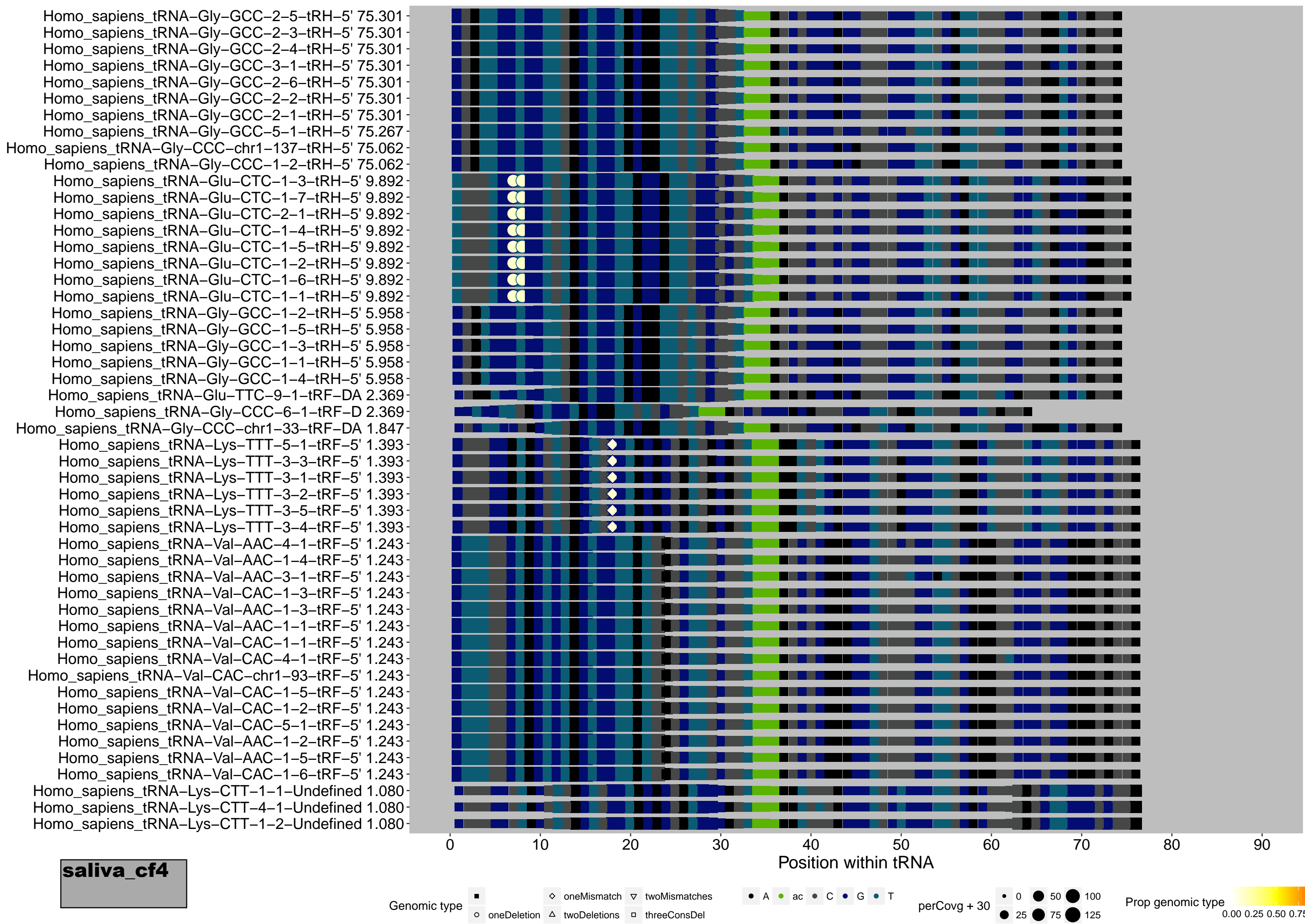

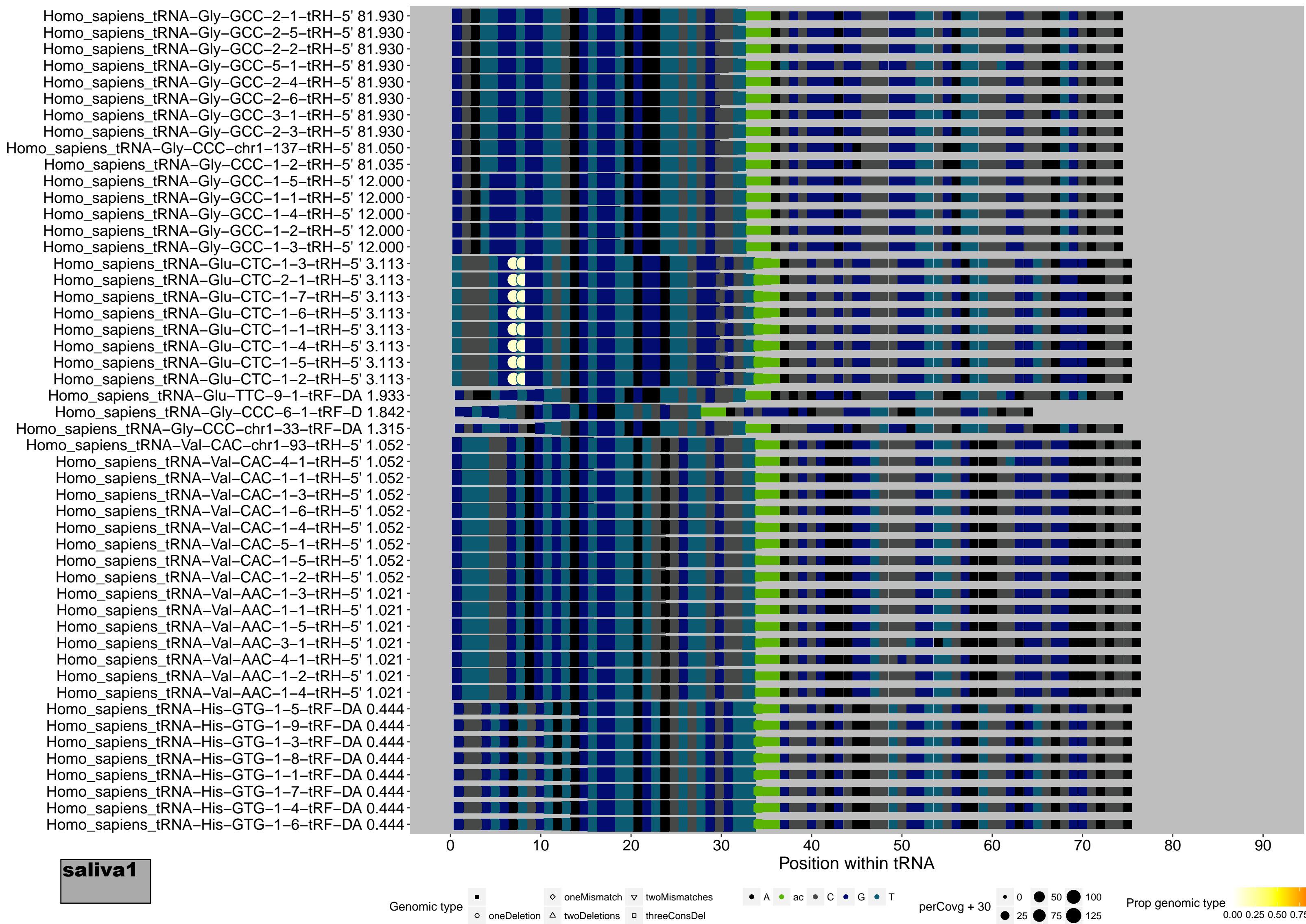

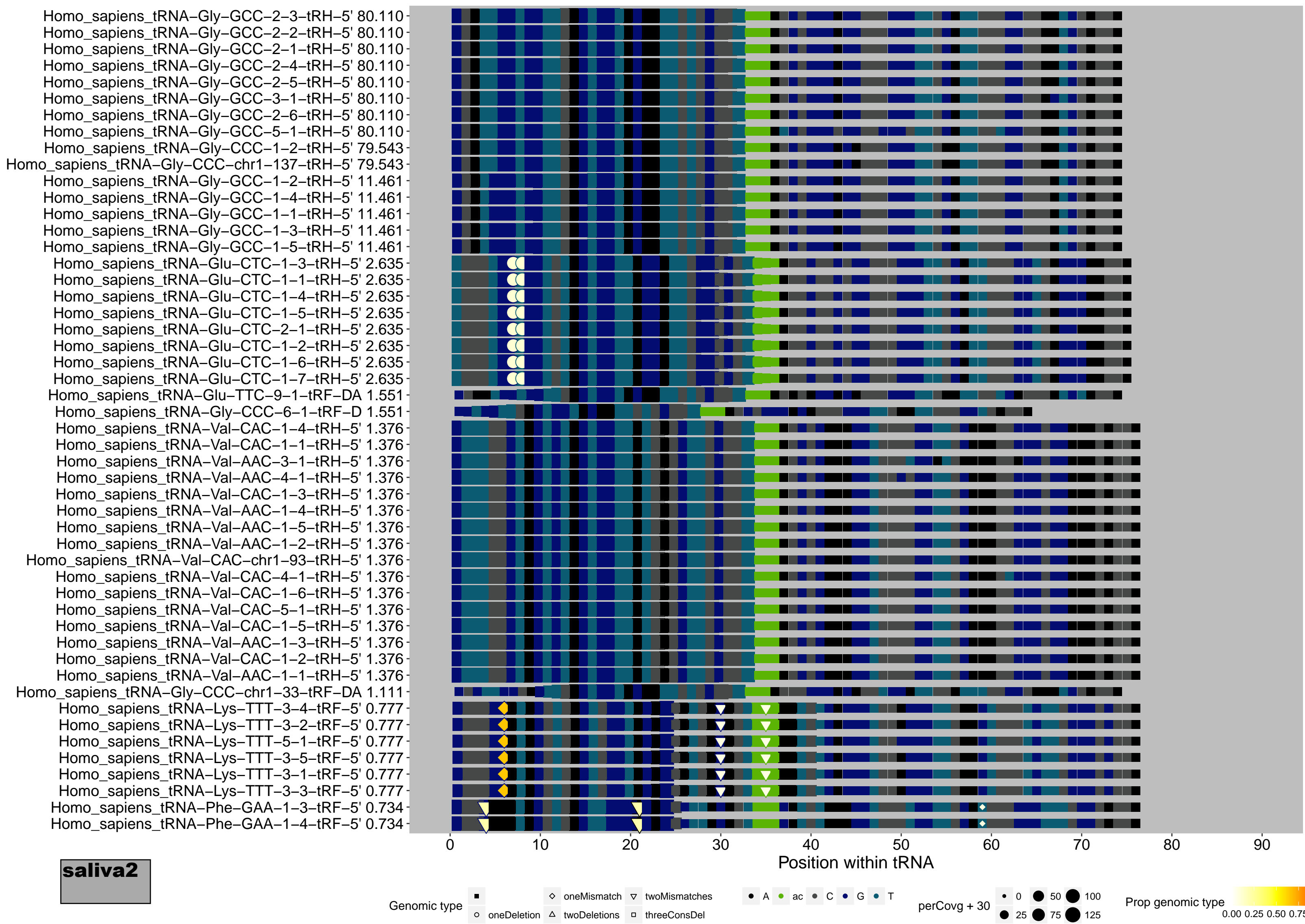

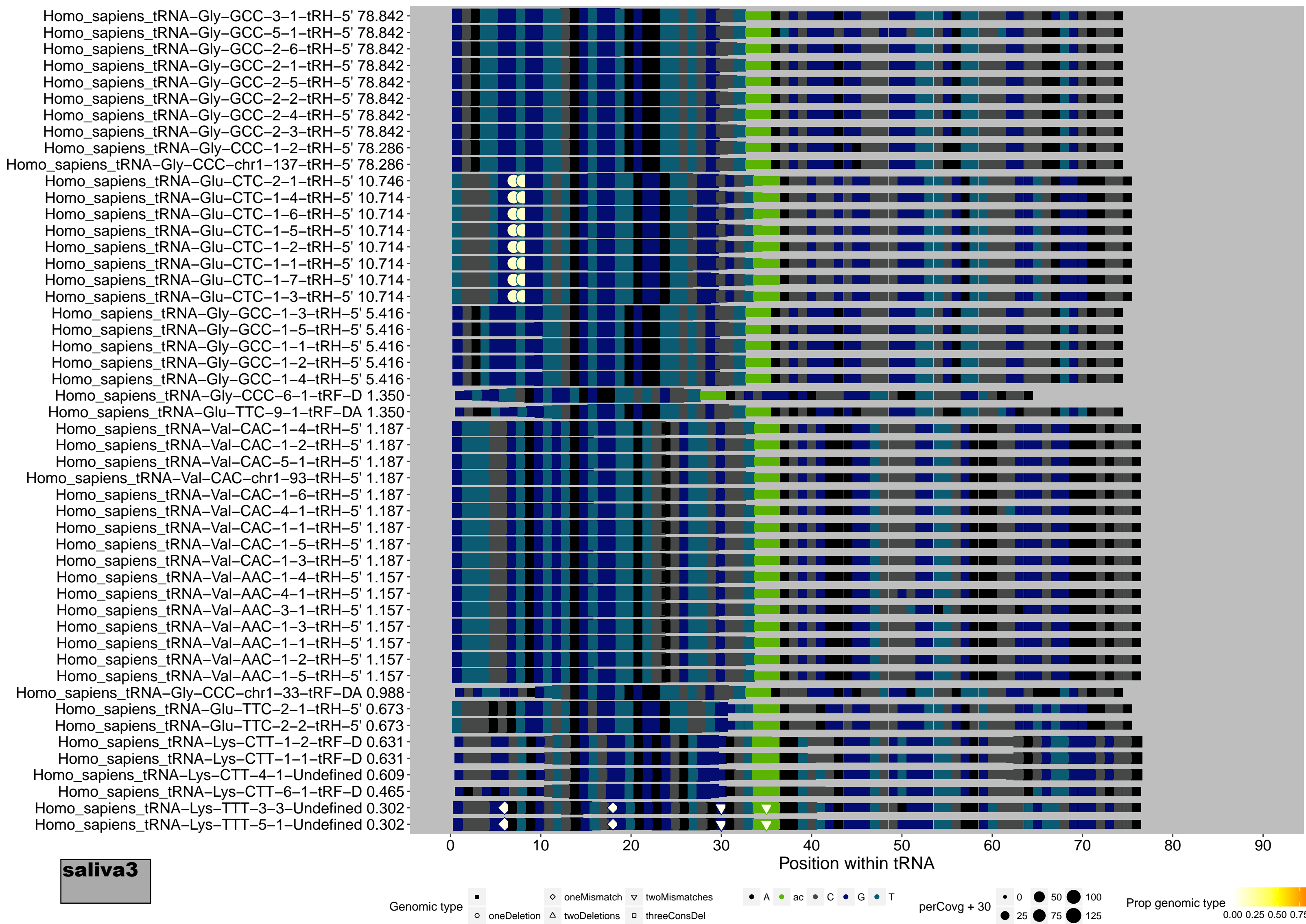

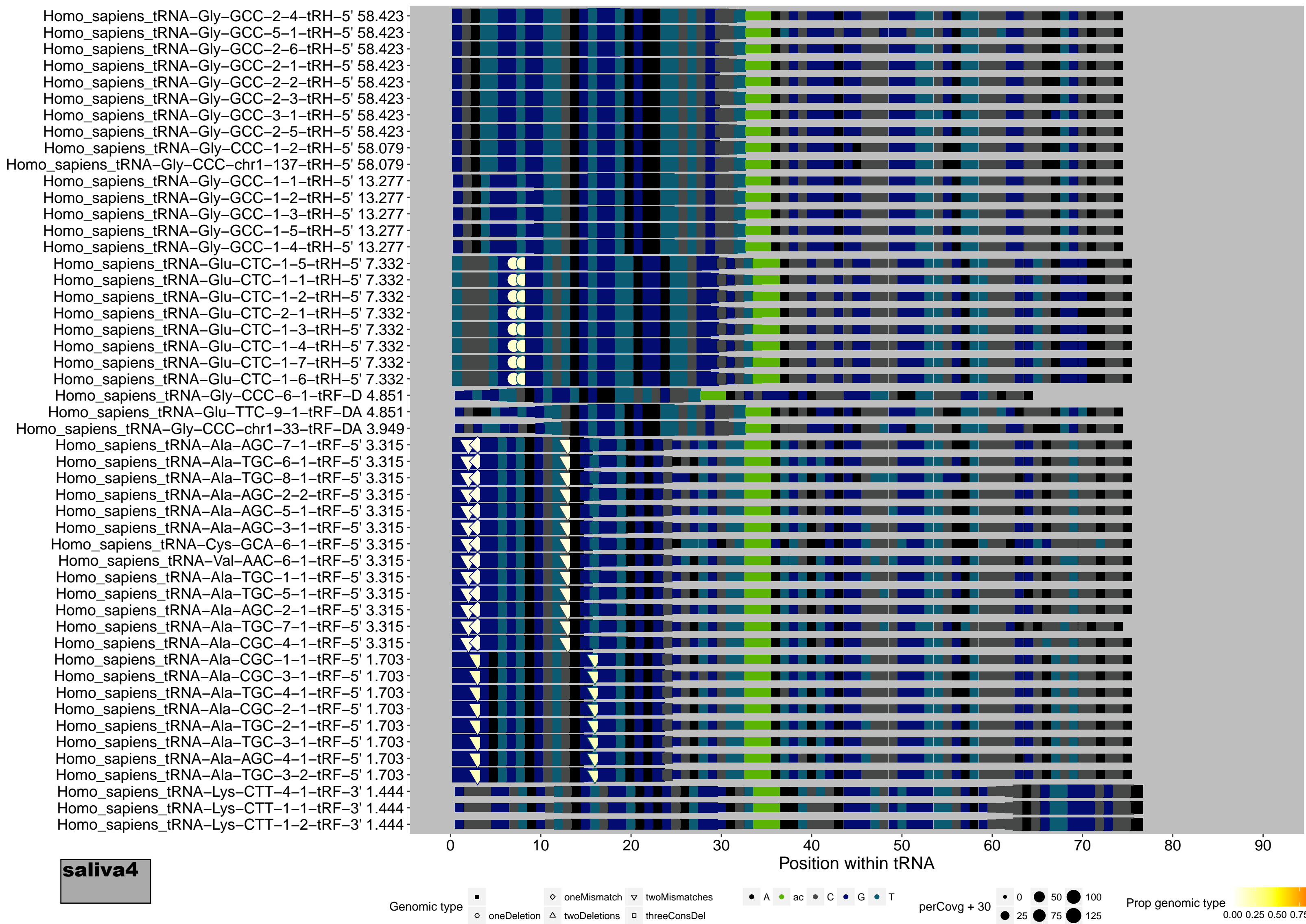

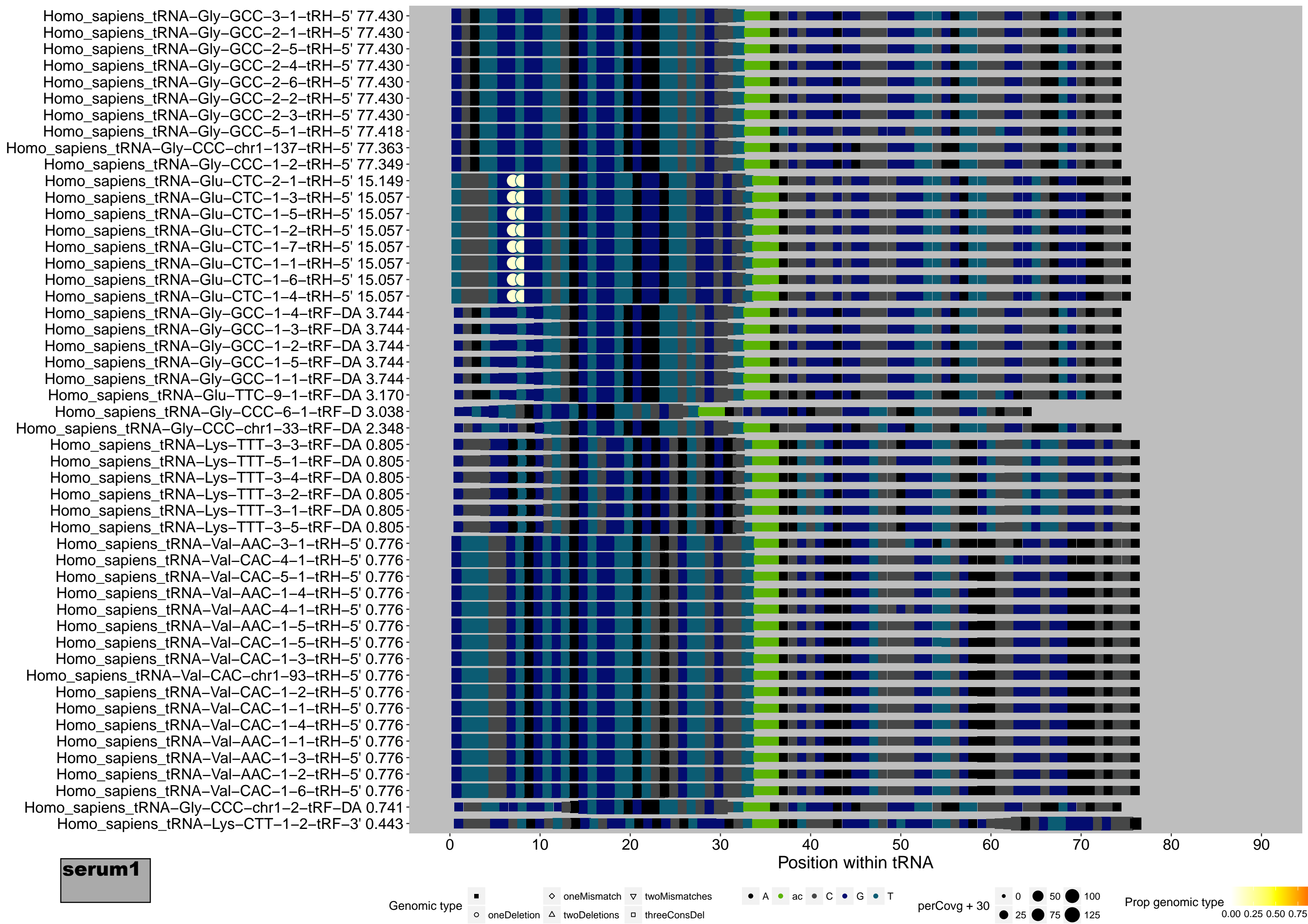

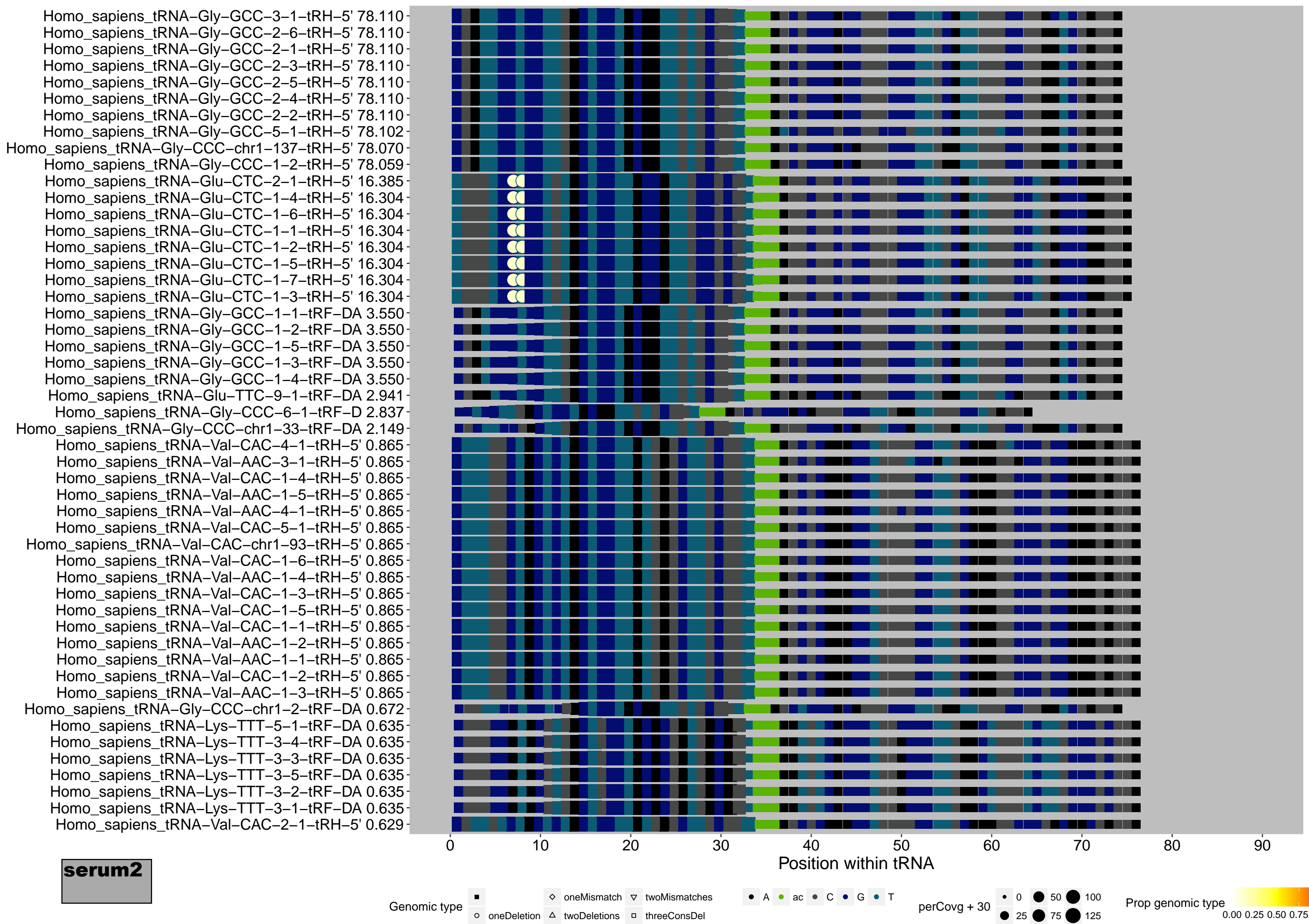

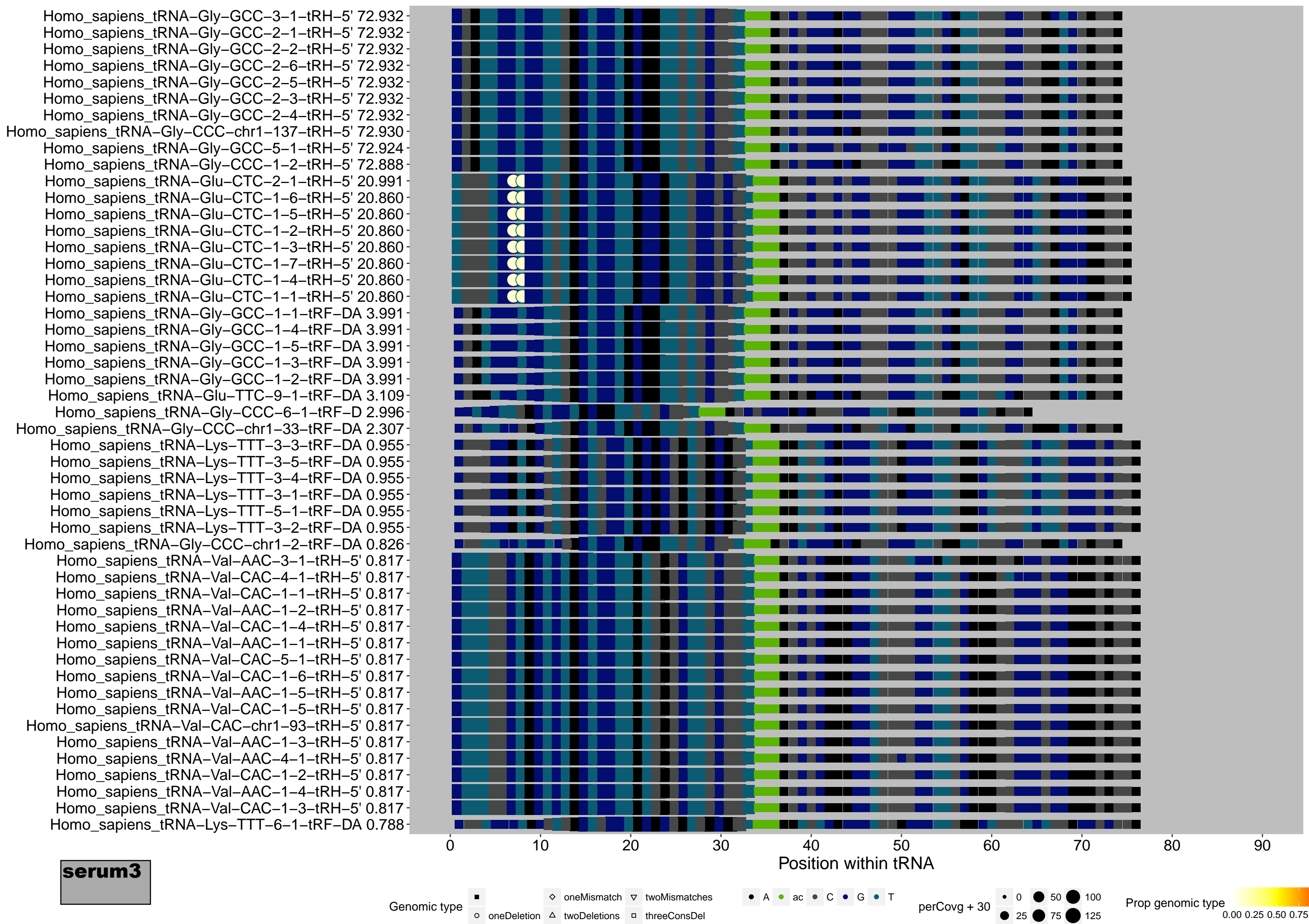

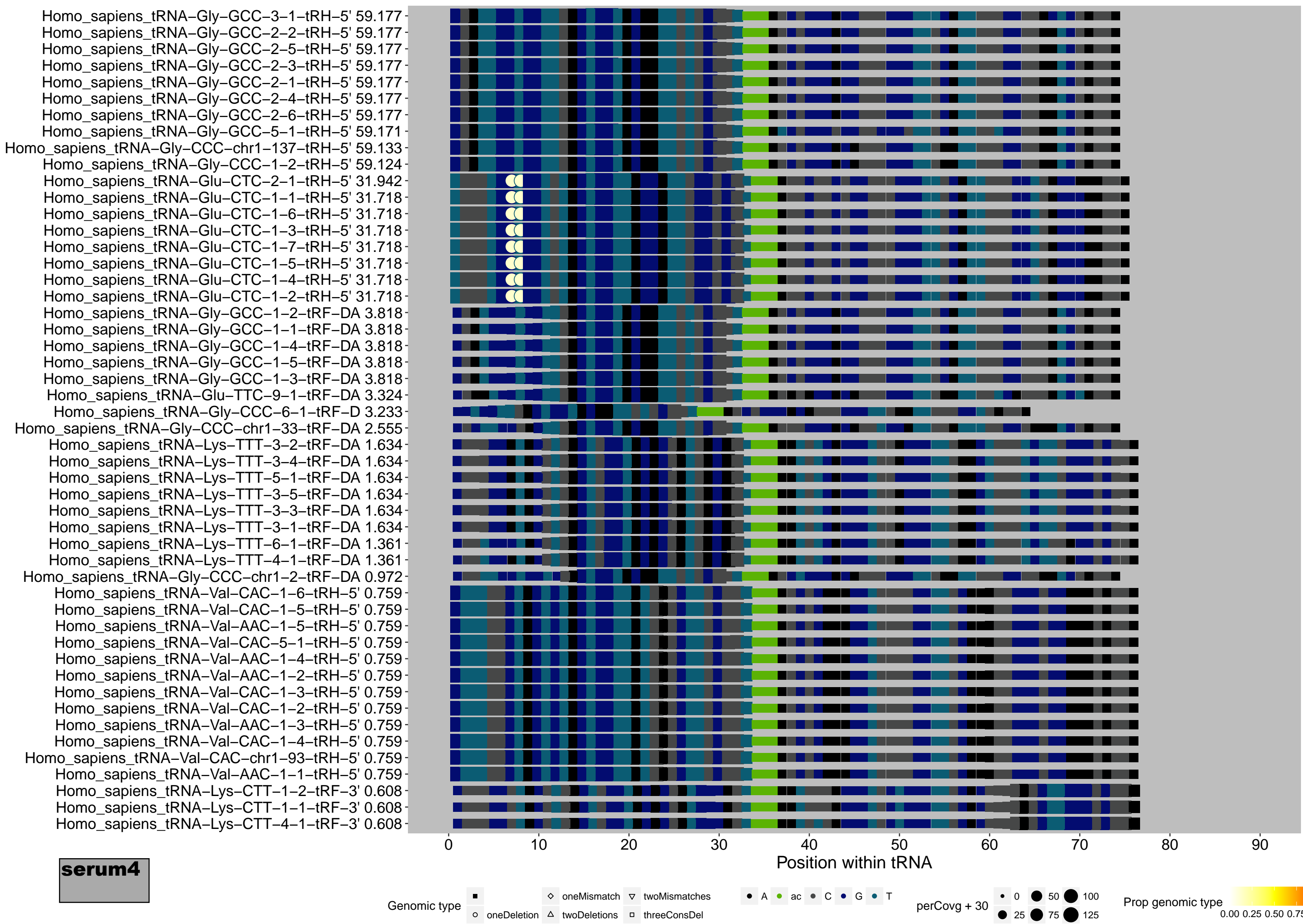

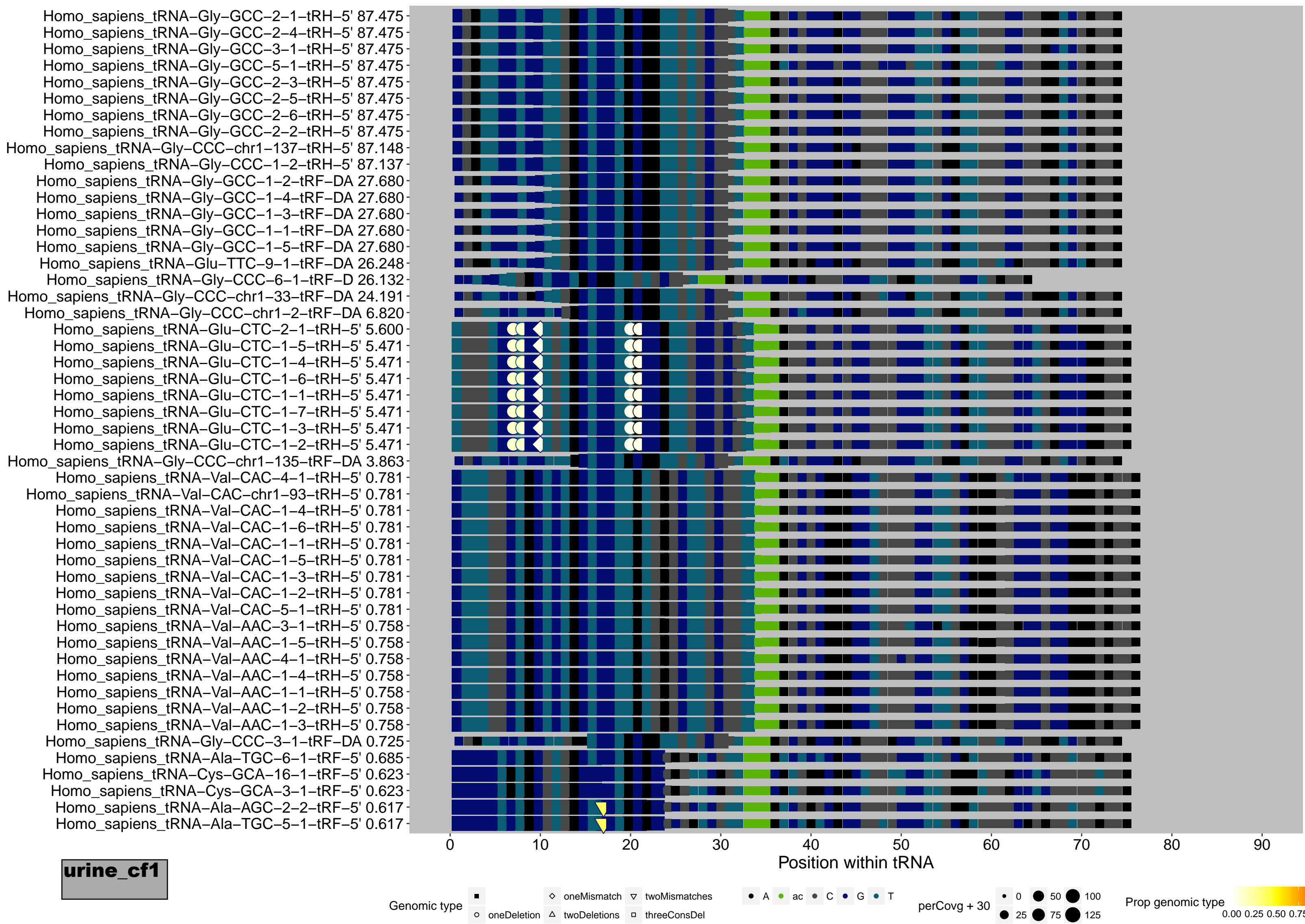



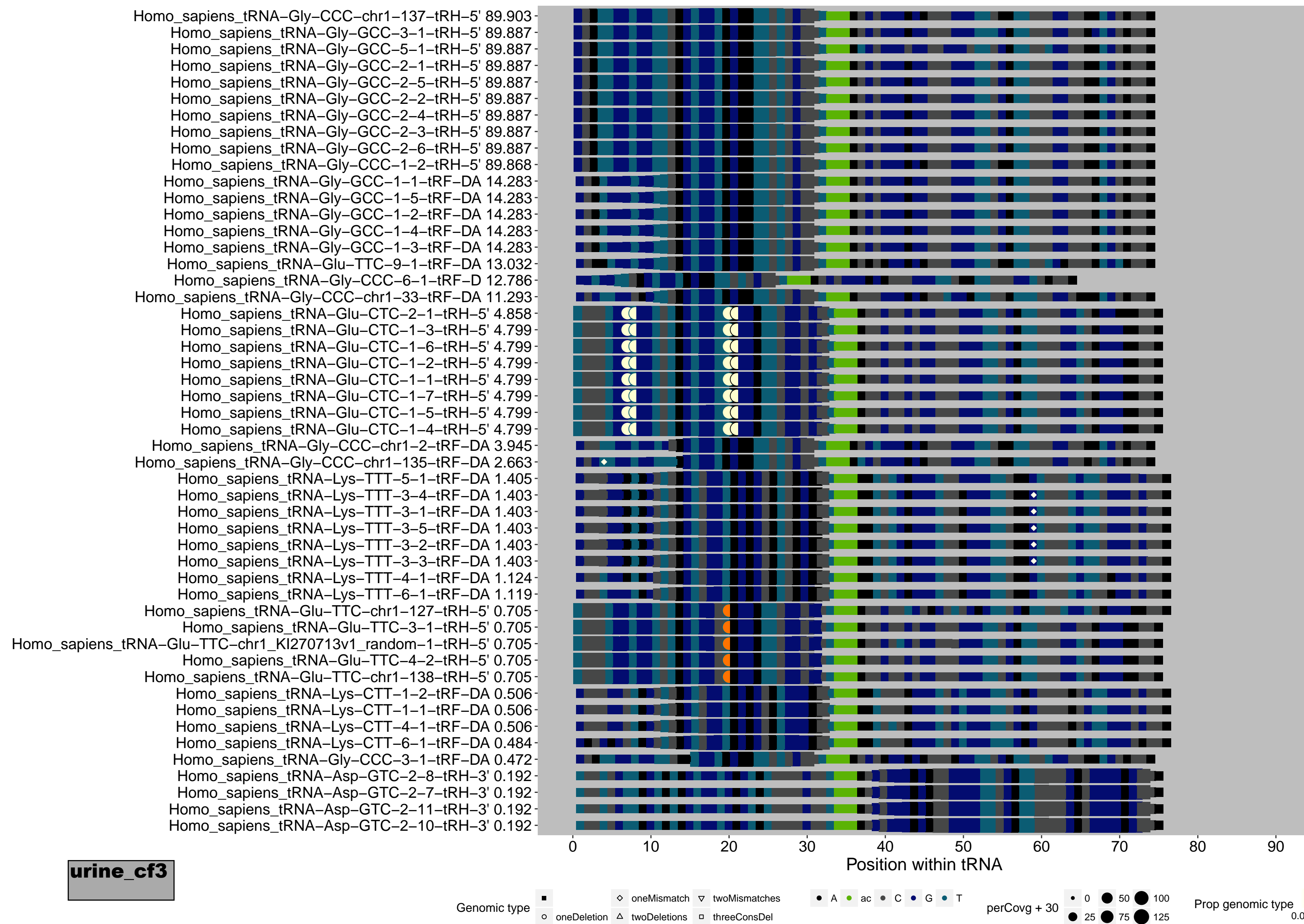

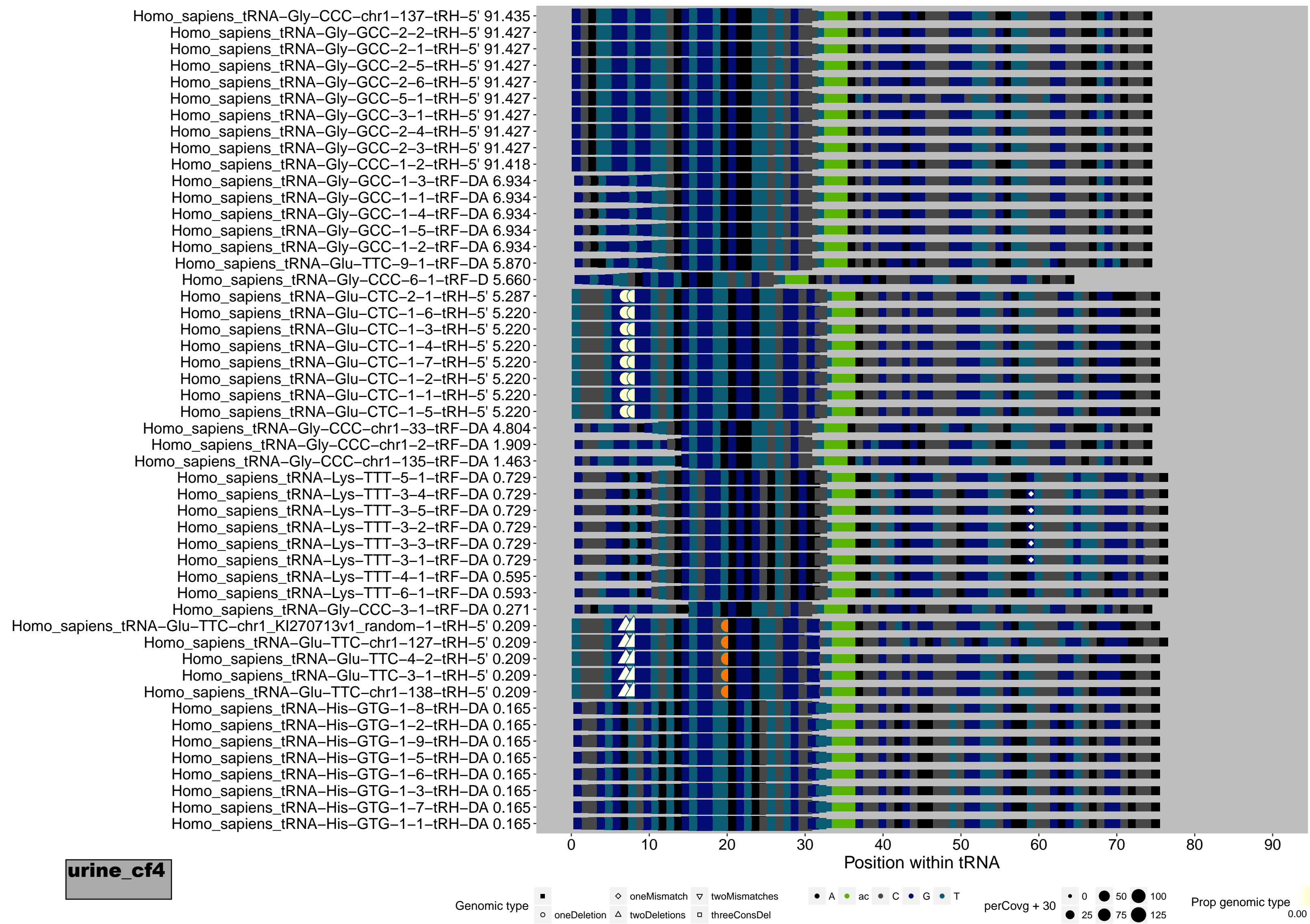

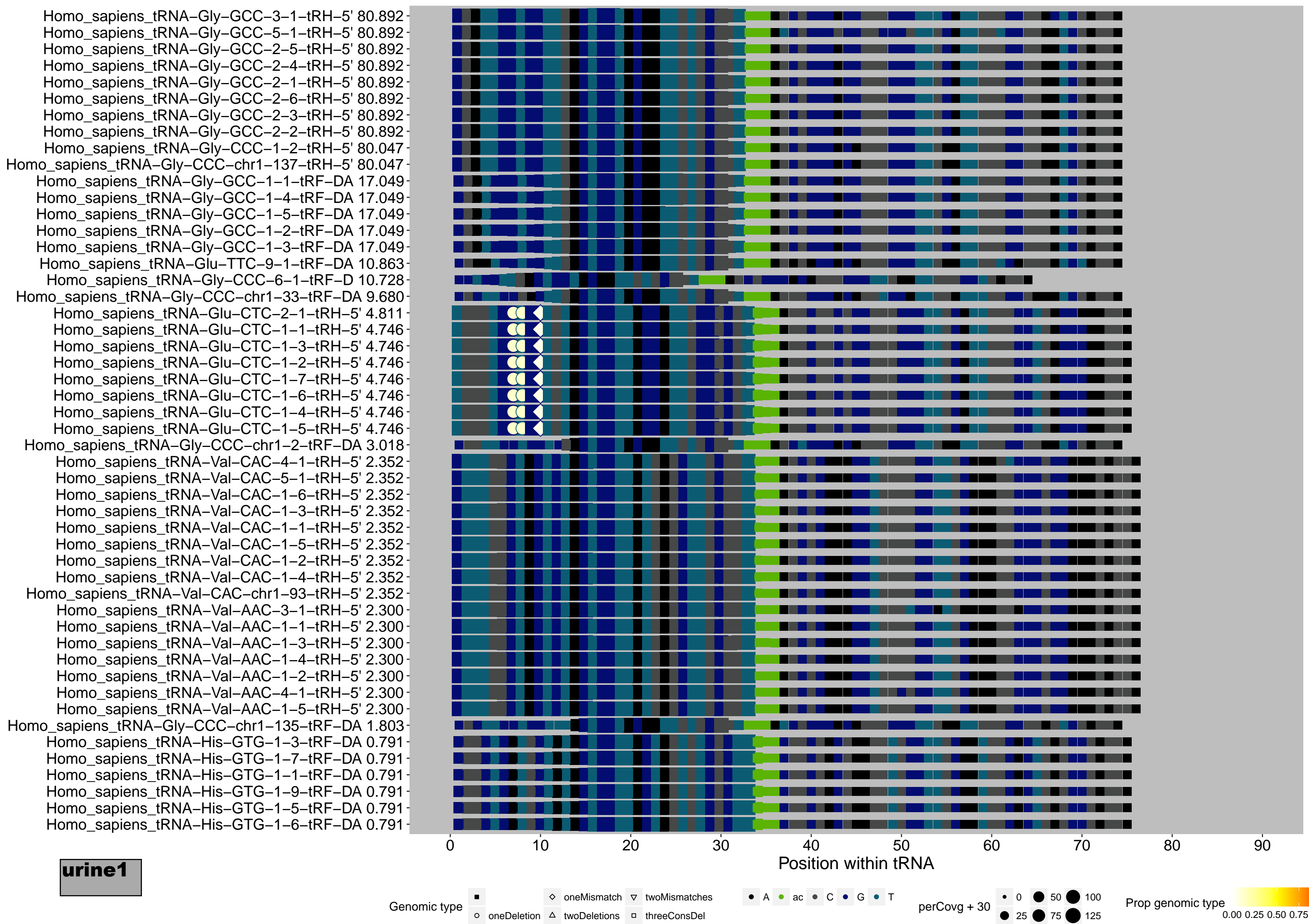

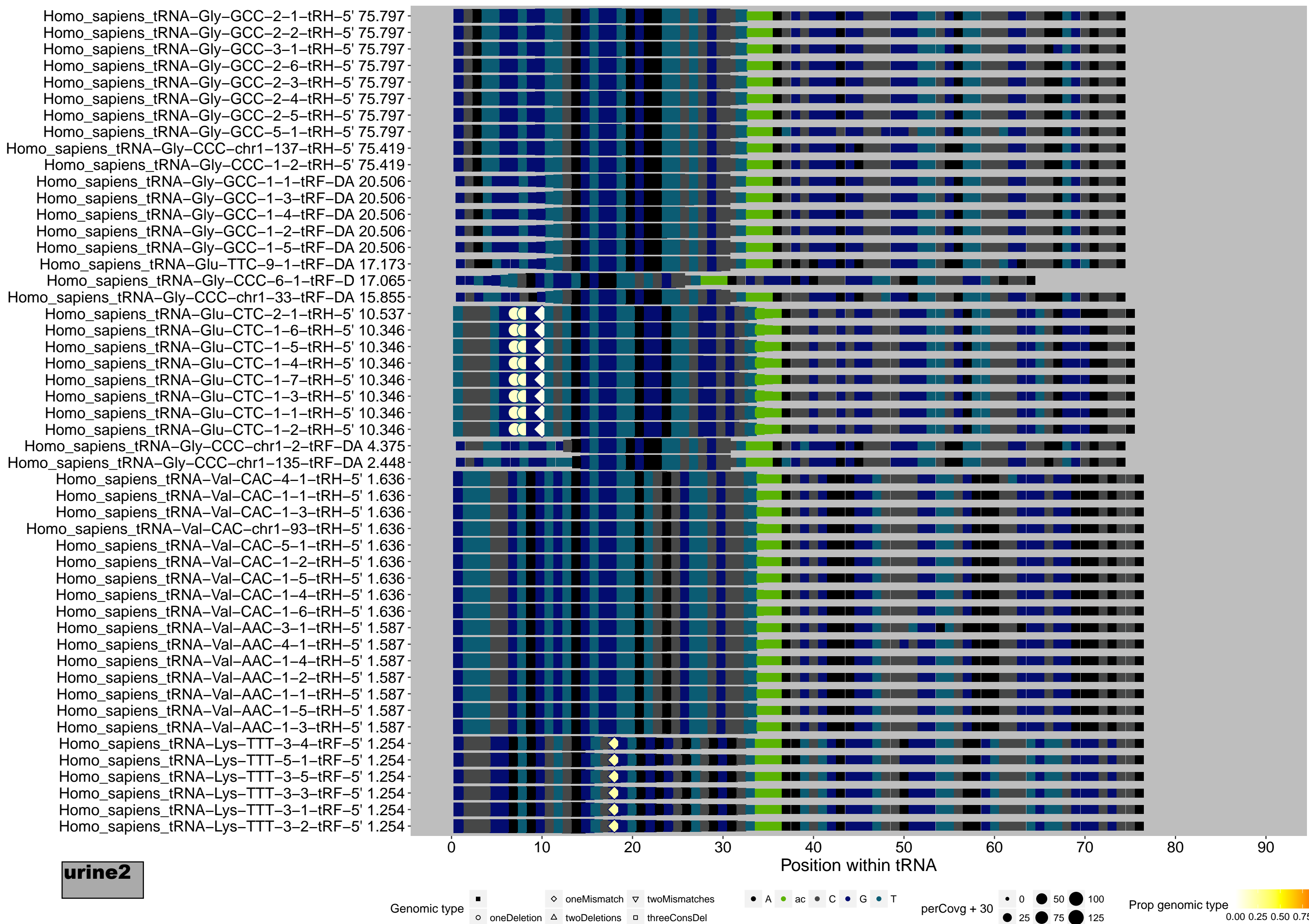

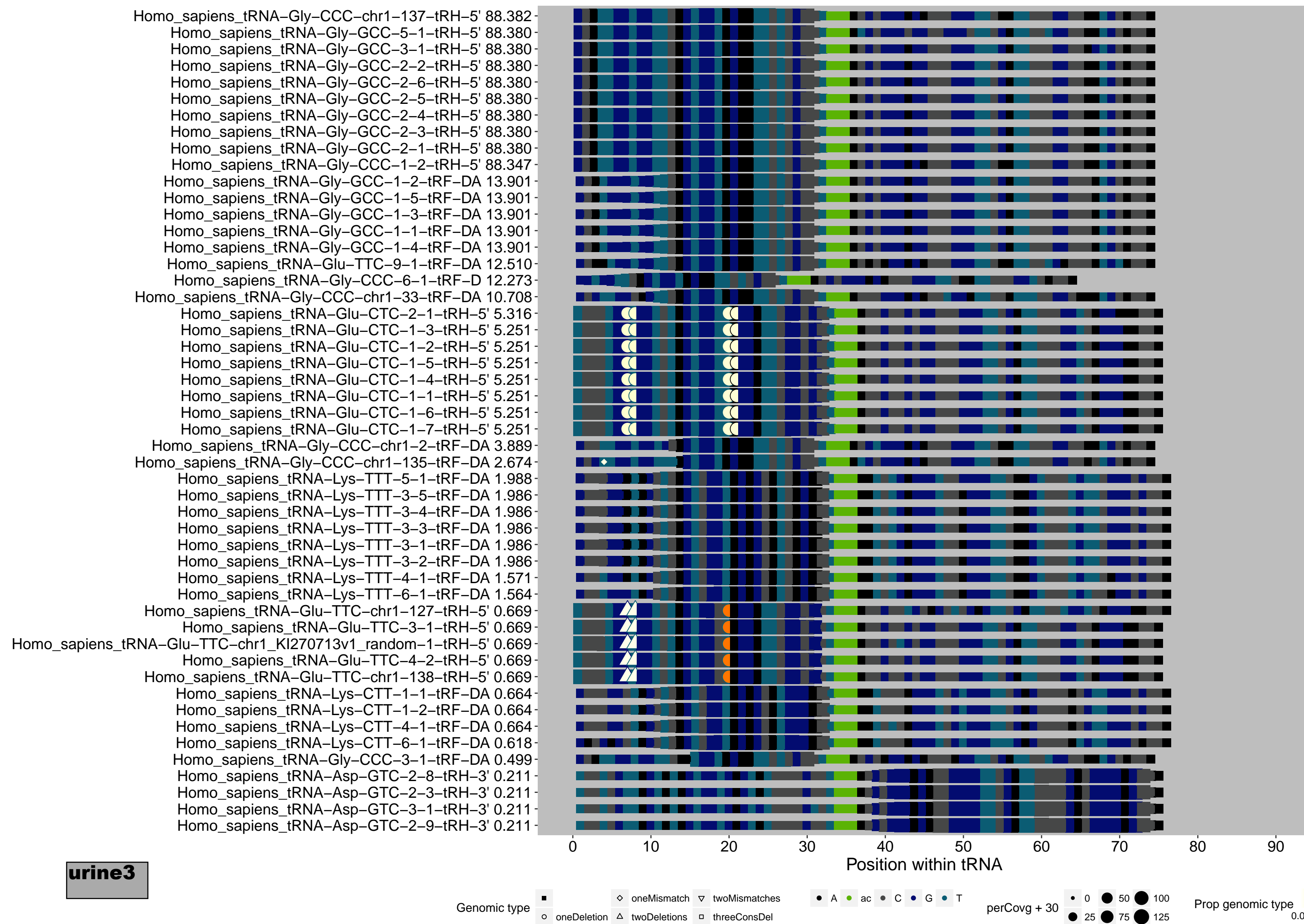

Supplement: Supplementary file 12 — Profiles of the top 50 mature tDR in each fluid. (PDF 4737 kb) [file 12864_2018_4785_MOESM12_ESM.pdf]

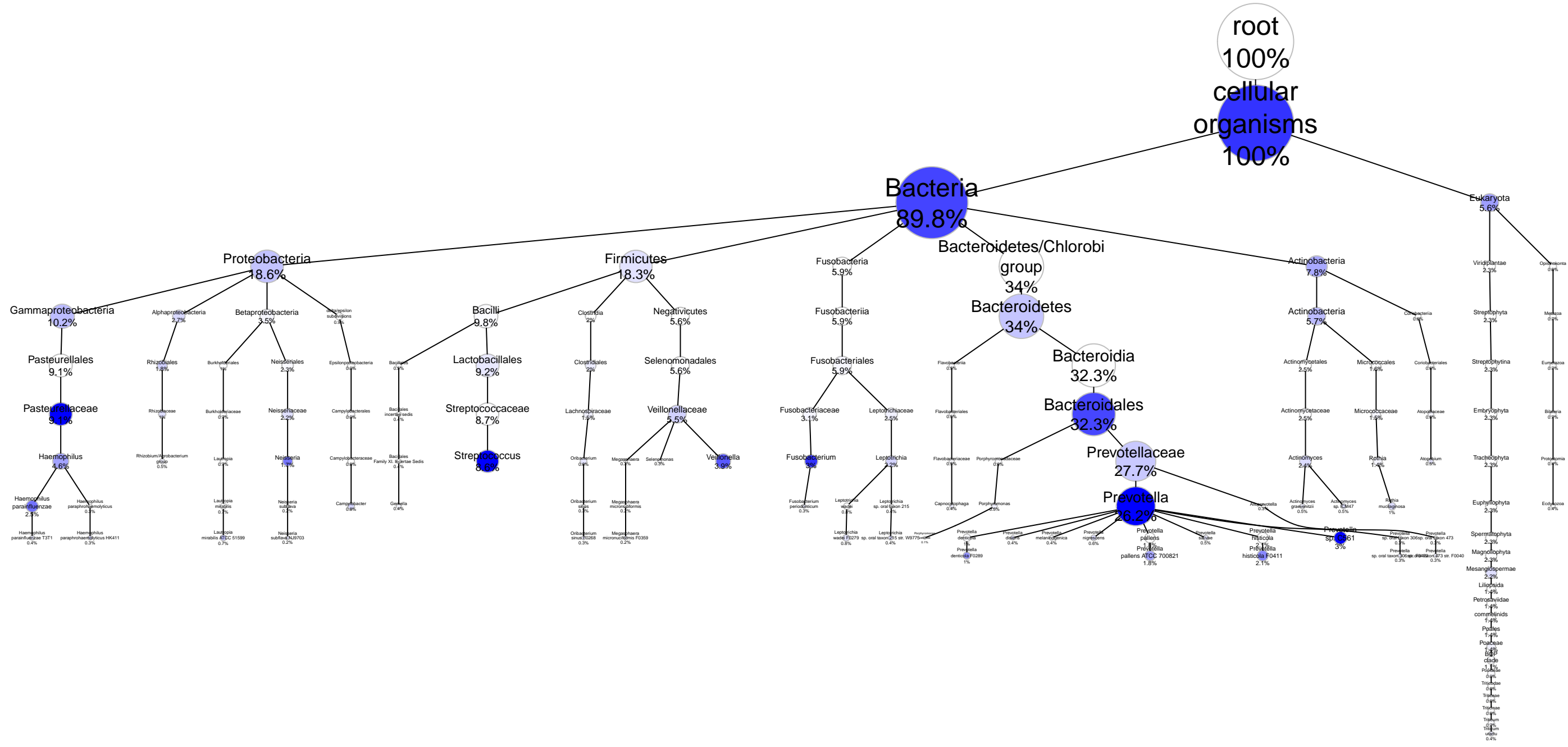

Supplement: Supplementary file 14 — Figure S3. Exogenous mapping of unmapped saliva reads. (PDF 24 kb) [file 12864_2018_4785_MOESM14_ESM.pdf]

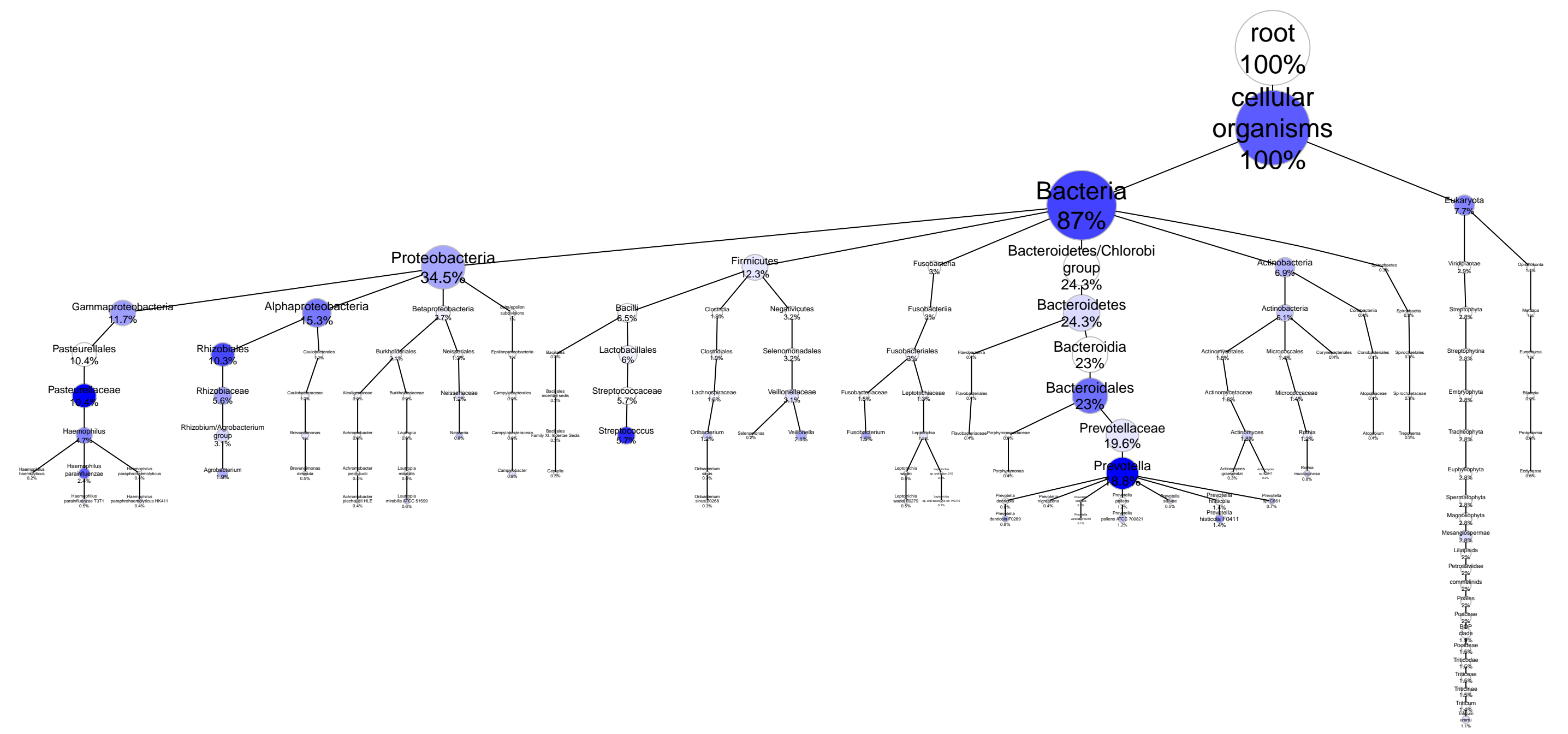

Supplement: Supplementary file 15 — Figure S4. Exogenous mapping of unmapped cell-free saliva reads. (PDF 24 kb) [file 12864_2018_4785_MOESM15_ESM.pdf]
