# Supplementary material for: Diversity and signature of small RNA in different bodily fluids using next generation sequencing
Source: BMC Genomics. 2018 May 29;19:408. doi: 10.1186/s12864-018-4785-8 (PMC5975555; doi:10.1186/s12864-018-4785-8)
Supplement: Supplementary file 6 — Table S4. Common miRNAs between all fluids. (DOCX 13 kb) [file 12864_2018_4785_MOESM6_ESM.docx]

**Additional file 5: Table S4.** Common miRNAs between all fluids.

| hsa-let-7b-5p | hsa-let-7c-5p | hsa-let-7d-5p | hsa-let-7e-5p | hsa-let-7g-5p |
| --- | --- | --- | --- | --- |
| hsa-let-7i-5p | hsa-miR-100-5p | hsa-miR-101-3p | hsa-miR-103a-3p | hsa-miR-106b-3p |
| hsa-miR-107 | hsa-miR-10a-5p | hsa-miR-10b-5p | hsa-miR-122-5p | hsa-miR-1246 |
| hsa-miR-125a-5p | hsa-miR-125b-2-3p | hsa-miR-125b-5p | hsa-miR-126-3p | hsa-miR-1271-5p |
| hsa-miR-127-3p | hsa-miR-128-3p | hsa-miR-1301-3p | hsa-miR-1307-3p | hsa-miR-140-3p |
| hsa-miR-142-5p | hsa-miR-143-3p | hsa-miR-144-3p | hsa-miR-144-5p | hsa-miR-145-3p |
| hsa-miR-146a-5p | hsa-miR-146b-5p | hsa-miR-148a-3p | hsa-miR-148a-5p | hsa-miR-148b-3p |
| hsa-miR-150-5p | hsa-miR-151a-3p | hsa-miR-151a-5p | hsa-miR-151b | hsa-miR-152-3p |
| hsa-miR-15b-5p | hsa-miR-16-5p | hsa-miR-17-5p | hsa-miR-181a-2-3p | hsa-miR-181a-5p |
| hsa-miR-181b-5p | hsa-miR-181d-5p | hsa-miR-182-5p | hsa-miR-183-5p | hsa-miR-185-5p |
| hsa-miR-186-5p | hsa-miR-191-5p | hsa-miR-192-5p | hsa-miR-193a-5p | hsa-miR-194-5p |
| hsa-miR-197-3p | hsa-miR-199a-3p | hsa-miR-199a-5p | hsa-miR-199b-3p | hsa-miR-19b-3p |
| hsa-miR-200a-3p | hsa-miR-200b-3p | hsa-miR-200c-3p | hsa-miR-203a-3p | hsa-miR-205-5p |
| hsa-miR-20a-5p | hsa-miR-2110 | hsa-miR-21-3p | hsa-miR-21-5p | hsa-miR-221-3p |
| hsa-miR-221-5p | hsa-miR-222-3p | hsa-miR-223-3p | hsa-miR-22-3p | hsa-miR-224-5p |
| hsa-miR-23a-3p | hsa-miR-23b-3p | hsa-miR-24-3p | hsa-miR-25-3p | hsa-miR-26a-5p |
| hsa-miR-26b-5p | hsa-miR-27a-3p | hsa-miR-27b-3p | hsa-miR-27b-5p | hsa-miR-28-3p |
| hsa-miR-28-5p | hsa-miR-29a-3p | hsa-miR-30a-3p | hsa-miR-30a-5p | hsa-miR-30b-5p |
| hsa-miR-30c-5p | hsa-miR-30d-5p | hsa-miR-30e-3p | hsa-miR-30e-5p | hsa-miR-320a |
| hsa-miR-320b | hsa-miR-330-3p | hsa-miR-335-5p | hsa-miR-338-5p | hsa-miR-340-5p |
| hsa-miR-342-3p | hsa-miR-345-5p | hsa-miR-361-3p | hsa-miR-361-5p | hsa-miR-363-3p |
| hsa-miR-374a-5p | hsa-miR-374b-5p | hsa-miR-375 | hsa-miR-378a-3p | hsa-miR-409-3p |
| hsa-miR-421 | hsa-miR-423-3p | hsa-miR-423-5p | hsa-miR-425-3p | hsa-miR-425-5p |
| hsa-miR-450b-5p | hsa-miR-451a | hsa-miR-484 | hsa-miR-500a-3p | hsa-miR-501-3p |
| hsa-miR-503-5p | hsa-miR-532-5p | hsa-miR-584-5p | hsa-miR-589-5p | hsa-miR-628-3p |
| hsa-miR-629-5p | hsa-miR-652-3p | hsa-miR-664a-5p | hsa-miR-744-5p | hsa-miR-7-5p |
| hsa-miR-769-5p | hsa-miR-92a-3p | hsa-miR-92b-3p | hsa-miR-93-5p | hsa-miR-941 |
| hsa-miR-96-5p | hsa-miR-98-5p | hsa-miR-99a-5p | hsa-miR-99b-5p |  |
