# Supplementary material for: Diversity and signature of small RNA in different bodily fluids using next generation sequencing
Source: BMC Genomics. 2018 May 29;19:408. doi: 10.1186/s12864-018-4785-8 (PMC5975555; doi:10.1186/s12864-018-4785-8)
Supplement: Supplementary file 7 — Table S5. Unique miRNAs detected in each bodily fluid. (DOCX 14 kb) [file 12864_2018_4785_MOESM7_ESM.docx]

**Additional file 6: Table S5.** Unique miRNAs detected in each bodily fluid.

| **Blood** | **Blood** | **Blood** | **Leukocytes** | **Plasma** | **Plasma** |
| --- | --- | --- | --- | --- | --- |
| hsa-miR-130b-3p | hsa-miR-6815-5p | hsa-miR-210-5p | hsa-miR-873-3p | hsa-miR-3130-3p | hsa-miR-6770-3p |
| hsa-miR-3140-3p | hsa-miR-4755-5p | hsa-miR-3130-5p | hsa-miR-338-3p | hsa-miR-23a-5p | hsa-miR-541-3p |
| hsa-miR-324-5p | hsa-miR-636 | hsa-miR-1227-3p | hsa-miR-1291 | hsa-miR-4665-5p | hsa-miR-539-5p |
| hsa-miR-4685-3p | hsa-miR-4668-5p | hsa-miR-378d | hsa-miR-618 | hsa-miR-3677-3p | hsa-miR-939-5p |
| hsa-miR-550a-3-5p | hsa-miR-548ad-5p/548ae-5p | hsa-miR-6882-5p | hsa-miR-1248 | hsa-miR-758-5p | hsa-miR-6875-5p |
| hsa-miR-6514-5p | hsa-miR-4440 | hsa-miR-3661 | hsa-miR-873-5p | hsa-miR-2355-3p | hsa-miR-556-5p |
| hsa-miR-3942-5p | hsa-miR-4482-3p | hsa-miR-378g | hsa-miR-378a-5p | hsa-miR-3174 | hsa-miR-7848-3p |
| hsa-miR-580-3p | hsa-miR-1224-5p | hsa-miR-4775 | hsa-miR-98-3p | hsa-miR-548n | hsa-miR-6892-5p |
| hsa-miR-3691-5p | hsa-miR-548ay-3p | hsa-miR-6767-5p | hsa-miR-671-5p | hsa-miR-1229-3p | hsa-miR-6852-3p |
| hsa-miR-6734-5p | hsa-miR-363-5p | hsa-miR-7855-5p | hsa-miR-6503-3p | hsa-miR-4687-5p | hsa-miR-431-3p |
| hsa-miR-29b-2-5p | hsa-let-7c | hsa-miR-6741-5p | hsa-miR-664b-3p | hsa-miR-5187-5p | hsa-miR-6732-3p |
| hsa-miR-6866-5p | hsa-miR-4755-3p | hsa-miR-3176 | hsa-miR-590-3p | hsa-miR-6847-5p | hsa-miR-329-5p |
| hsa-miR-3944-5p | hsa-miR-6749-3p | hsa-miR-3164 | hsa-miR-181b-3p | hsa-miR-3161 | **Serum** |
| hsa-miR-942-3p | hsa-miR-4753-5p | hsa-miR-6879-3p | hsa-miR-3651 | hsa-miR-6819-3p | hsa-miR-323a-5p |
| hsa-miR-3688-3p | hsa-miR-3200-5p | hsa-miR-6780a-5p | hsa-miR-4521 | hsa-miR-4449 | **Saliva** |
| hsa-miR-1303 | hsa-miR-3163 | hsa-miR-6836-3p | hsa-miR-504-5p | hsa-miR-548j-5p | hsa-miR-34a-5p |
| hsa-miR-190a-5p | hsa-miR-6884-5p | hsa-miR-3146 | hsa-let-7f-2-3p | hsa-miR-26a-1-3p | **Cell-Free Saliva** |
| hsa-miR-5009-5p | hsa-miR-5706 | hsa-miR-6750-5p | hsa-miR-7977 | hsa-miR-6798-3p | hsa-miR-2115-3p |
| hsa-miR-5010-3p | hsa-miR-3912-3p | hsa-miR-502-5p | hsa-miR-1296-5p | hsa-miR-3198 | hsa-miR-1262 |
| hsa-miR-5581-3p | hsa-miR-18b-5p | hsa-miR-4999-5p | hsa-miR-491-5p | hsa-miR-6837-3p | hsa-miR-1290 |
| hsa-miR-6747-3p | hsa-miR-584-3p | hsa-miR-5003-3p | hsa-miR-6813-3p | hsa-miR-369-5p | **Urine** |
| hsa-miR-3135a | hsa-miR-3913-5p | hsa-miR-5582-3p | hsa-miR-548w | hsa-miR-135a-3p | hsa-miR-489-3p |
| hsa-miR-1304-5p | hsa-miR-3143 | hsa-miR-4646-5p | hsa-miR-92a-1-5p | hsa-miR-3188 | **Cell-Free Urine** |
| hsa-miR-20b-3p | hsa-miR-6777-3p | hsa-miR-1285-5p | hsa-miR-4772-5p | hsa-miR-1914-5p | hsa-miR-194-3p |
| hsa-miR-3934-5p | hsa-miR-3682-3p | hsa-miR-5695 | hsa-miR-616-5p | hsa-miR-3150a-5p | hsa-miR-891a-5p |
| hsa-miR-501-5p | hsa-miR-18b-3p | hsa-miR-1299 | hsa-miR-376c-3p | hsa-miR-6810-3p | hsa-miR-206 |
| hsa-miR-548ac | hsa-miR-3200-3p | hsa-miR-6853-3p | hsa-let-7f-1-3p | hsa-miR-7110-3p |  |
| hsa-miR-550a-3p | hsa-miR-4504 | hsa-miR-548al | hsa-miR-26a-2-3p | hsa-miR-6810-5p |  |
| hsa-miR-5001-3p | hsa-miR-451b | hsa-miR-301a-3p | hsa-let-7g-3p | hsa-miR-1273h-5p |  |
| hsa-miR-4742-3p | hsa-miR-4672 | hsa-miR-4669 | hsa-miR-337-3p | hsa-miR-5587-3p |  |
| hsa-miR-1289 | hsa-miR-548av-3p/548o-3p | hsa-miR-7155-3p |  |  |  |
| hsa-miR-320e |  |  |  |  |  |
