# Supplementary material for: Diversity and signature of small RNA in different bodily fluids using next generation sequencing
Source: BMC Genomics. 2018 May 29;19:408. doi: 10.1186/s12864-018-4785-8 (PMC5975555; doi:10.1186/s12864-018-4785-8)
Supplement: Supplementary file 9 — Table S7. Unique miRNAs detected in the non-invasive bodily fluids. (DOCX 13 kb) [file 12864_2018_4785_MOESM9_ESM.docx]

**Additional file 8: Table S7.** Unique miRNAs detected in the non-invasive bodily fluids.

| **Cell-Free Saliva** | **Cell-Free Saliva** | **Cell-Free Saliva** | **Cell-Free Urine** |
| --- | --- | --- | --- |
| hsa-miR-130b-5p | hsa-miR-1273h-3p | hsa-miR-1262 | hsa-miR-370-3p |
| hsa-miR-3928-3p | hsa-miR-143-5p | hsa-miR-1290 | hsa-miR-30d-3p |
| hsa-miR-26b-3p | hsa-miR-371b-5p | hsa-miR-212-5p | hsa-miR-4662a-5p |
| hsa-miR-550a-3-5p/550a-5p | hsa-miR-2115-3p | hsa-miR-7976 | hsa-miR-1-3p |
| hsa-miR-7706 | hsa-miR-335-3p |  | hsa-miR-500b-3p |
| hsa-miR-671-3p | hsa-let-7b-3p | **Saliva** | hsa-miR-485-5p |
| hsa-miR-4677-3p | hsa-miR-3177-3p | hsa-miR-34a-5p | hsa-miR-99b-3p |
| hsa-miR-25-5p | hsa-miR-103a-2-5p |  | hsa-miR-486-3p |
| hsa-miR-3909 | hsa-miR-454-5p | **Urine** | hsa-miR-194-3p |
| hsa-miR-574-5p | hsa-miR-542-3p | hsa-miR-432-5p | hsa-miR-891a-5p |
| hsa-miR-6842-3p | hsa-miR-582-5p | hsa-miR-411-5p | hsa-miR-206 |
| hsa-miR-328-3p | hsa-miR-2355-5p | hsa-miR-3607-3p |  |
| hsa-miR-664a-3p | hsa-miR-3614-5p | hsa-miR-489-3p |  |
| hsa-miR-942-5p | hsa-miR-183-3p |  |  |
