# Supplementary material for: Diversity and signature of small RNA in different bodily fluids using next generation sequencing
Source: BMC Genomics. 2018 May 29;19:408. doi: 10.1186/s12864-018-4785-8 (PMC5975555; doi:10.1186/s12864-018-4785-8)
Supplement: Supplementary file 13 — Table S9. piRNAs that represent an average of 1% or more of the entire piRNA counts of each bodily fluid. (DOCX 18 kb) [file 12864_2018_4785_MOESM13_ESM.docx]

**Additional file 10: Table S9.** piRNAs that represent an average of 1% or more of the entire piRNA counts of each bodily fluid.

| **Body fluid** | **piRNA** | **GenBank Accession number** | **Chromosomal position** | **Percentage** |
| --- | --- | --- | --- | --- |
| **Blood** | hsa-piR-016658 | DQ592931 | Homo sapiens:6:80508363:80508389:Plus | 91.9±1.9 |
|  | hsa-piR-016659 | DQ592932 | Homo sapiens:14:22388242:22388267:Plus | 3.9±0.9 |
|  | hsa-piR-000805 | DQ571003 | Homo sapiens:1:212438966:212438997:Plus | 1.5±0.3 |
| **Leukocyte** | hsa-piR-016658 | DQ592931 | Homo sapiens:6:80508363:80508389:Plus | 70.6±3.8 |
|  | hsa-piR-000552 | DQ570687 | Homo sapiens:22:38045003:38045030:Minus | 5.3±0.6 |
|  | hsa-piR-018780 | DQ595807 | Homo sapiens:17:72068837:72068864:Plus | 3.9±1.0 |
|  | hsa-piR-017033 | DQ593538 | Homo sapiens:20:17891355:17891384:Minus | 2.7±0.6 |
|  | hsa-piR-012753 | DQ587269 | Homo sapiens:2:232029453:232029478:Minus | 1.5±0.3 |
|  | hsa-piR-020813 | DQ598646 | Homo sapiens:1:31213600:31213629:Minus | 1.5±0.4 |
|  | hsa-piR-000805 | DQ571003 | Homo sapiens:1:212438966:212438997:Plus | 1.1±0.2 |
| **Plasma** | hsa-piR-016658 | DQ592931 | Homo sapiens:6:80508363:80508389:Plus | 78.2±39.6 |
|  | hsa-piR-019825 | DQ597218 | Homo sapiens:1:227740227:227740256:Plus | 8.1±16.2 |
|  | hsa-piR-020365 | DQ597975 | Homo sapiens:11:108979133:108979162:Plus | 4.8±9.6 |
|  | hsa-piR-021639 | DQ599671 | Homo sapiens:6:71627734:71627759:Minus | 1.3±2.5 |
| **Serum** | hsa-piR-016658 | DQ592931 | Homo sapiens:6:80508363:80508389:Plus | 91.0±4.4 |
|  | hsa-piR-016659 | DQ592932 | Homo sapiens:14:22388242:22388267:Plus | 1.4±0.1 |
| **Saliva** | hsa-piR-016658 | DQ592931 | Homo sapiens:6:80508363:80508389:Plus | 12.1±3.0 |
|  | hsa-piR-014620 | DQ590013 | Homo sapiens:5:93930930:93930956:Minus | 10.8±6.4 |
|  | hsa-piR-019521 | DQ596805 | Homo sapiens:11:10487516:10487542:Minus | 7.5±1.2 |
|  | hsa-piR-000552 | DQ570687 | Homo sapiens:22:38045003:38045030:Minus | 5.6±2.6 |
|  | hsa-piR-018780 | DQ595807 | Homo sapiens:17:72068837:72068864:Plus | 4.2±2.9 |
|  | hsa-piR-000805 | DQ571003 | Homo sapiens:1:212438966:212438997:Plus | 3.8±1.3 |
|  | hsa-piR-017033 | DQ593538 | Homo sapiens:20:17891355:17891384:Minus | 3.4±1.9 |
|  | hsa-piR-020450 | DQ598104 | Homo sapiens:9:133350930:133350959:Plus | 3.2±1.8 |
|  | hsa-piR-020828 | DQ598675 | Homo sapiens:2:149355860:149355890:Minus | 3.1±1.2 |
|  | hsa-piR-020009 | DQ597484 | Homo sapiens:7:145325417:145325443:Plus | 2.5±1.4 |
|  | hsa-piR-012753 | DQ587269 | Homo sapiens:2:232029453:232029478:Minus | 2.3±1.6 |
|  | hsa-piR-020008 | DQ597482 | Homo sapiens:11:10487020:10487050:Minus | 2.2±1.3 |
|  | hsa-piR-001170 | DQ571526 | Homo sapiens:11:10488300:10488331:Minus | 2.0±1.9 |
|  | hsa-piR-020813 | DQ598646 | Homo sapiens:1:31213600:31213629:Minus | 2.0±1.6 |
|  | hsa-piR-001101 | DQ571419 | Homo sapiens:1:45016687:45016714:Plus | 1.9±1.0 |
|  | hsa-piR-010894 | DQ584698 | Homo sapiens:8:68660258:68660288:Minus | 1.8±1.3 |
|  | hsa-piR-001169 | DQ571525 | Homo sapiens:17:21945564:21945589:Plus | 1.4±2.3 |
|  | hsa-piR-021532 | DQ599542 | Homo sapiens:16:74190973:74191001:Minus | 1.2±1.0 |
| **Cell-Free Saliva** | hsa-piR-016658 | DQ592931 | Homo sapiens:6:80508363:80508389:Plus | 23.3±7.1 |
|  | hsa-piR-021532 | DQ599542 | Homo sapiens:16:74190973:74191001:Minus | 7.5±3.6 |
|  | hsa-piR-016659 | DQ592932 | Homo sapiens:14:22388242:22388267:Plus | 3.7±2.5 |
|  | hsa-piR-019521 | DQ596805 | Homo sapiens:11:10487516:10487542:Minus | 2.6±1.4 |
|  | hsa-piR-000552 | DQ570687 | Homo sapiens:22:38045003:38045030:Minus | 2.5±2.0 |
|  | hsa-piR-020450 | DQ598104 | Homo sapiens:9:133350930:133350959:Plus | 2.4±1.0 |
|  | hsa-piR-020008 | DQ597482 | Homo sapiens:11:10487020:10487050:Minus | 2.2±1.6 |
|  | hsa-piR-014620 | DQ590013 | Homo sapiens:5:93930930:93930956:Minus | 2.1±1.6 |
|  | hsa-piR-012753 | DQ587269 | Homo sapiens:2:232029453:232029478:Minus | 1.9±2.2 |
|  | hsa-piR-000805 | DQ571003 | Homo sapiens:1:212438966:212438997:Plus | 1.8±0.7 |
|  | hsa-piR-020813 | DQ598646 | Homo sapiens:1:31213600:31213629:Minus | 1.3±1.4 |
|  | hsa-piR-017791 | DQ594556 | Homo sapiens:11:74789084:74789113:Plus | 1.3±2.2 |
|  | hsa-piR-008624 | DQ581708 | Homo sapiens:12:9410818:9410847:Minus | 1.3±0.6 |
|  | hsa-piR-023114 | DQ601614 | Homo sapiens:11:45656844:45656873:Minus | 1.3±1.5 |
|  | hsa-piR-019909 | DQ597338 | Homo sapiens:11:45656845:45656870:Minus | 1.3±1.5 |
|  | hsa-piR-020828 | DQ598675 | Homo sapiens:2:149355860:149355890:Minus | 1.2±0.8 |
|  | hsa-piR-020009 | DQ597484 | Homo sapiens:7:145325417:145325443:Plus | 1.1±0.8 |
|  | hsa-piR-019420 | DQ596670 | Homo sapiens:2:101255914:101255940:Minus | 1±0.9 |
| **Urine** | hsa-piR-019825 | DQ597218 | Homo sapiens:1:227740227:227740256:Plus | 42.3±42.0 |
|  | hsa-piR-016658 | DQ592931 | Homo sapiens:6:80508363:80508389:Plus | 9.3±5.7 |
|  | hsa-piR-021532 | DQ599542 | Homo sapiens:16:74190973:74191001:Minus | 4.4±7.4 |
|  | hsa-piR-004153 | DQ575660 | Homo sapiens:3:156861576:156861607:Plus | 4.2±4.1 |
|  | hsa-piR-014620 | DQ590013 | Homo sapiens:5:93930930:93930956:Minus | 3.0±2.1 |
|  | hsa-piR-002352 | DQ573172 | Homo sapiens:15:49351595:49351625:Minus | 2.3±3.3 |
|  | hsa-piR-019521 | DQ596805 | Homo sapiens:11:10487516:10487542:Minus | 2.2±1.9 |
|  | hsa-piR-005019 | DQ576918 | Homo sapiens:2:27127216:27127243:Plus | 1.9±1.0 |
|  | hsa-piR-020450 | DQ598104 | Homo sapiens:9:133350930:133350959:Plus | 1.8±1.5 |
|  | hsa-piR-000805 | DQ571003 | Homo sapiens:1:212438966:212438997:Plus | 1.5±1.5 |
|  | hsa-piR-019420 | DQ596670 | Homo sapiens:2:101255914:101255940:Minus | 1.4±1.0 |
|  | hsa-piR-003242 | DQ574395 | Homo sapiens:20:61668910:61668939:Minus | 1.2±1.9 |
| **Cell-Free Urine** | hsa-piR-019825 | DQ597218 | Homo sapiens:1:227740227:227740256:Plus | 47.5±40.5 |
|  | hsa-piR-016658 | DQ592931 | Homo sapiens:6:80508363:80508389:Plus | 6.8±2.9 |
|  | hsa-piR-004153 | DQ575660 | Homo sapiens:3:156861576:156861607:Plus | 4.8±4.3 |
|  | hsa-piR-002352 | DQ573172 | Homo sapiens:15:49351595:49351625:Minus | 4.1±6.6 |
|  | hsa-piR-021532 | DQ599542 | Homo sapiens:16:74190973:74191001:Minus | 3.5±5.1 |
|  | hsa-piR-005019 | DQ576918 | Homo sapiens:2:27127216:27127243:Plus | 2.1±1.2 |
|  | hsa-piR-014620 | DQ590013 | Homo sapiens:5:93930930:93930956:Minus | 1.8±1.8 |
|  | hsa-piR-019420 | DQ596670 | Homo sapiens:2:101255914:101255940:Minus | 1.3±1.2 |
|  | hsa-piR-003242 | DQ574395 | Homo sapiens:20:61668910:61668939:Minus | 1.1±1.9 |
